# On–off switch of charge-separated states of pyridine-vinylene-linked porphyrin–C_60_ conjugates detected by EPR†
†Electronic supplementary information (ESI) available: Experimental details and procedures, complete spectroscopic and structural analysis, including Fig. S1–S51, Schemes S1 and S2 and Tables T1–T3. See DOI: 10.1039/c5sc02051d



**DOI:** 10.1039/c5sc02051d

**Published:** 2015-07-09

**Authors:** Sabrina V. Kirner, Danny Arteaga, Christian Henkel, Johannes T. Margraf, Nuria Alegret, Kei Ohkubo, Braulio Insuasty, Alejandro Ortiz, Nazario Martín, Luis Echegoyen, Shunichi Fukuzumi, Timothy Clark, Dirk M. Guldi

**Affiliations:** a Department of Chemistry and Pharmacy and Interdisciplinary Center for Molecular Materials , Friedrich-Alexander-Universität Erlangen-Nürnberg , Egerlandstrasse 3 , 91058 Erlangen , Germany; b Departamento de Química , Facultad de Ciencias Naturales , Universidad del Valle , A.A. 25360 Cali , Colombia; c Department of Chemistry and Pharmacy , Computer Chemistry Center , Friedrich-Alexander-University Erlangen-Nürnberg , Nägelsbachstr. 25 , 91052 Erlangen , Germany; d Departament de Química Física i Inorgànica , Universitat Rovira i Virgili , 43007 , Tarragona , Spain; e Department of Material and Life Science , Graduate School of Engineering , Osaka University , ALCA and SENTAN , Japan Science and Technology Agency (JST) , Suita , Osaka 565-0871 , Japan; f Department of Bioinspired Science , Ewha Womans University , Seoul 120-750 , Korea; g Departamento de Química Orgánica , Facultad de Química , Universidad Complutense 28040 , Madrid , Spain; h Department of Chemistry , University of Texas at El Paso , El Paso , Texas 79968-0519 , USA; i Faculty of Science and Technology , Meijo University and ALCA and SENTAN , Japan Science and Technology Agency (JST) , Tempaku , Nagoya , Aichi 468-8502 , Japan

## Abstract

The design, synthesis, and electronic properties of a new series of D–π–A conjugates consisting of free base (H_2_P) and zinc porphyrins (ZnP) as electron donors and a fullerene (C_60_) as electron acceptor, in which the two electroactive entities are covalently linked through pyridine-vinylene spacers of different lengths, are described. Electronic interactions in the ground state were characterized by electrochemical and absorption measurements, which were further supported with theoretical calculations. Most importantly, charge-transfer bands were observed in the absorption spectra, indicating a strong *push*–*pull* behavior. In the excited states, electronic interactions were detected by selective photoexcitation under steady-state conditions, by time-resolved fluorescence investigations, and by pump probe experiments on the femto-, pico-, and nanosecond time scales. Porphyrin fluorescence is quenched for the different D–π–A conjugates, from which we conclude that the deactivation mechanisms of the excited singlet states are based on photoinduced energy- and/or electron transfer processes between H_2_P/ZnP and C_60_, mediated through the molecular spacers. The fluorescence intensity decreases and the fluorescence lifetimes shorten as the spacer length decreases and as the spacer substitution changes. With the help of transient absorption spectroscopy, the formation of charge-separated states involving oxidized H_2_P/ZnP and reduced C_60_ was confirmed. Lifetimes of the corresponding charge-separated states, which ranged from ∼400 picoseconds to 165 nanoseconds, depend on the spacer length, the spacer substitution, and the solvent polarity. Interestingly, D–π–A conjugates containing the longest linkers did not necessarily exhibit the longest charge-separated state lifetimes. The distances between the electron donors and the acceptors were calculated by molecular modelling. The longest charge-separated state lifetime corresponded to the D–π–A conjugate with the longest electron donor–acceptor distance. Likewise, EPR measurements in frozen media revealed charge separated states in all the D–π–A conjugates investigated. A sharp peak with *g* values ∼2.000 was assigned to reduced C_60_, while a broader, less intense signal (*g* ∼ 2.003) was assigned to oxidized H_2_P/ZnP. On–off switching of the formation and decay of the charge-separated states was detected by EPR at 77 K by repeatedly turning the irradiation source on and off.

## Abstract 2

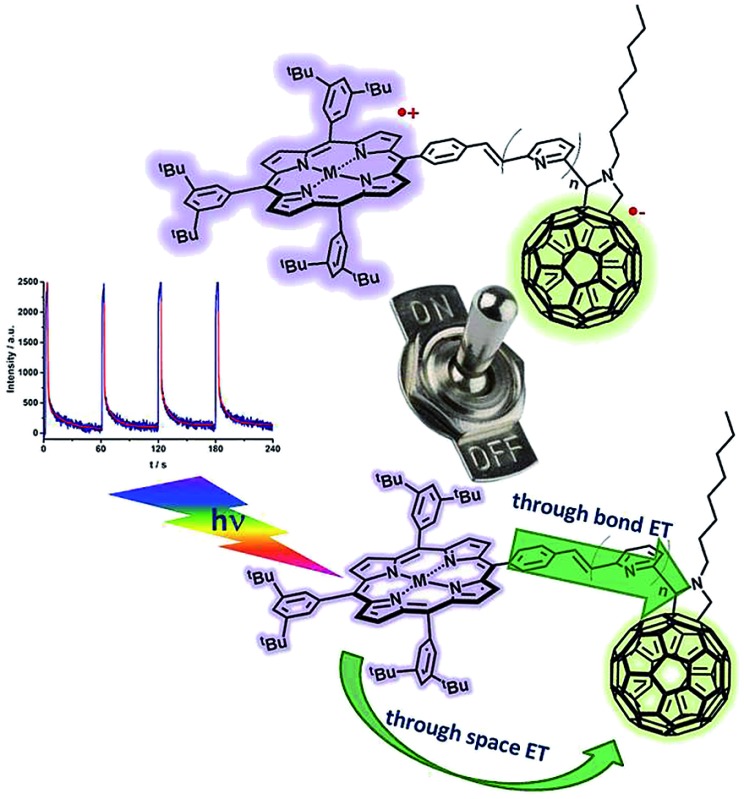
The on–off switch of charge separated states in a new series of pyridine-vinylene linked porphyrin–C_60_ conjugates was detected by EPR at 77 K.

## Introduction

In Nature, photosynthesis is by far the best method to convert solar energy into chemical energy. It involves complex processes based on intramolecular electron/energy transfer reactions between molecular components within photoactive membranes.^1–5^ In recent decades, photosynthesis has served as an inspiration to design and synthesize new artificial photosynthetic arrays that mimic the function of plants.^6^ In this context, organic chemists have prepared many artificial photosynthetic systems that have enabled the study of the fundamental chemistry and the reaction mechanisms involved in the biological processes that are responsible for solar energy conversion in nature.^7–10^

The synthesis of molecular architectures consisting of electron donors and acceptors, covalently linked by π-conjugated molecular spacers (D–π–A) is one of the strategies for probing photoinduced electron transfer processes on a molecular level.^8,11^ The electronic properties of these molecules make them potentially useful in molecular photonics, optoelectronics, nanoscale applications, and in solar energy conversion.^12–18^ Porphyrins represent an important class of molecular building blocks, which in biological architectures are responsible for oxygen-electron transport, light-to-energy conversion, *etc.* ^19^ They have been used frequently as electron donors in D–π–A conjugates, mainly because of their ease of synthesis, versatile electrochemical and photochemical properties, and their presence in the naturally occurring chlorophyll.^20–22^ As a complement, C_60_ is an excellent electron acceptor because it features low-energy triply degenerate LUMOs and is able to accept up to six electrons.^2,23–27^ C_60_ exhibits low reorganization energies upon electron transfer processes, which is essential to obtain ultrafast charge separation and slow charge recombination.^28–31^ Thus, a wide variety of porphyrin arrays – H_2_P or ZnP – have been covalently linked to C_60_ in many different ways and using various wire-like molecular spacers.^32–36^

It has been observed that the linker in D–π–A conjugates has a profound effect on the rates of the intramolecular photoinduced charge-transfer^37^ and on the mechanism by which the charge transfer occurs,^38,39^ which can be *via* “superexchange”-mediated coherent tunnelling between the electronic states of the D/A pair or *via* a “hopping” mechanism through localized electronic states on the linker.^40^

Both charge separation and recombination occur and are defined by the electron-transfer rate constant *k*_ET_ as *Ae* ^–*βR*_D–A_^, where *A* is the Arrhenius constant, *β* the damping factor, and *R*_D–A_ the distance between the electron donors and acceptors. A decreased damping factor therefore means an increase of the distance over which charges can be efficiently transported.^41–44^ *β* depends primarily on the length of the wire-like molecular spacer, conformational rigidity, and the electronic properties of the electron donors and acceptors.^45–48^

Several groups have studied π-conjugated oligomers as wire-like molecular spacers connecting the photoactive termini in H_2_P/C_60_ and ZnP/C_60_ conjugates. π-extended spacers such as *para*-phenylene vinylene (oPPV),^49^ [2,20]*para*cyclophaneoligophenylene-vinylene (*p*Cp-oPPV),^50–52^ oligothiophene (*n*OT),^53^ and oligothienylenevinylene (*n*TV)^54,55^ are ideal connectors for effective charge transfer from the electron donors to the acceptors with maximum rates and relatively small damping factors.^49–55^

**Chart 1:**
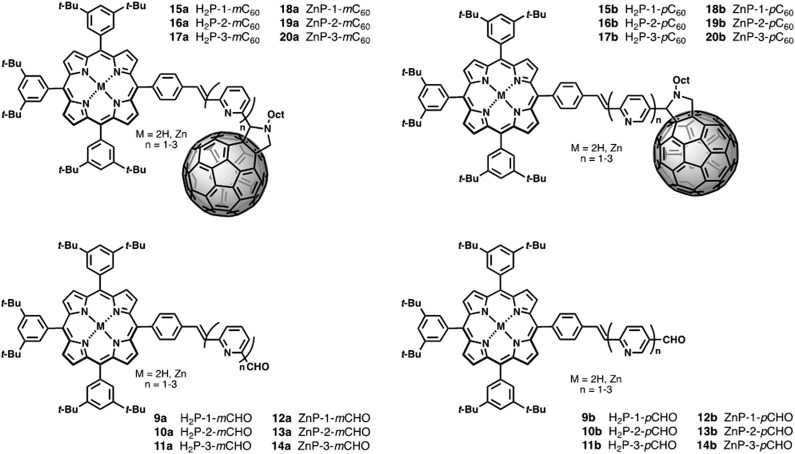
Above: Porphyrin–fullerene conjugates linked by pyridine-vinylene units at *meta*- and *para*-positions **15–20a** (H_2_P-*n-m*C_60_, ZnP-*n-m*C_60_, *n* = 1–3) and **15–20b** (H_2_P-*n-p*C_60_, and ZnP-*n-p*C_60_, *n* = 1–3). Below: Corresponding porphyrin–pyridine-vinylene references **9–14a** (H_2_P-*n-m*CHO, ZnP-*n-m*CHO, *n* = 1–3) and **9–14b** (H_2_P-*n-p*CHO, ZnP-*n-p*CHO, *n* = 1–3).

π-deficient molecular linkers, such as pyridine-vinylene groups have not been investigated so far, so we now report the synthesis and electronic/photophysical properties of a new homologous series of H_2_P/C_60_ and ZnP/C_60_ electron donor–acceptor conjugates bridged covalently by various pyridine-vinylene linkers **15–20a** (H_2_P-*n-m*C_60_, ZnP-*n-m*C_60_, *n* = 1–3) and **15–20b** (H_2_P-*n-p*C_60_, ZnP-*n-p*C_60_, *n* = 1–3) in addition to their precursors **9–14a** (H_2_P-*n-m*CHO, ZnP-*n-m*CHO, *n* = 1–3) and **9–14b** (H_2_P-n-*p*CHO, ZnP-*n-p*CHO, *n* = 1–3). We have used pyridine-vinylene spacers of different lengths and substitution patterns to conduct a systematic evaluation of the influence of both the length and nature of the linker on intramolecular charge-transfer processes that yield long- and short-lived charge-separated states in solvents of different polarities (Chart 1).

## Results

### Synthesis

**Scheme 1:**
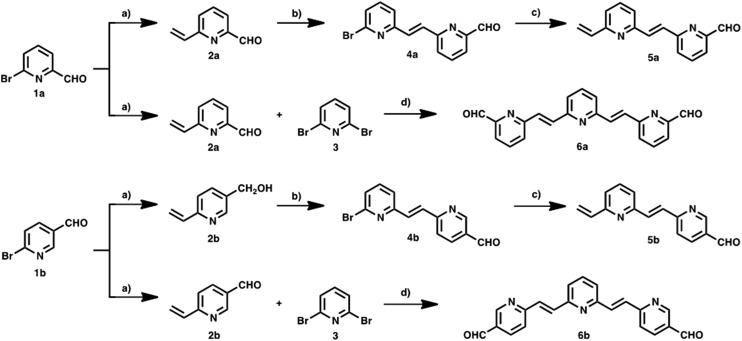
Synthetic route for the preparation of pyridine-vinylene linkers: top **2–6a** and bottom **2–6b**. Reagents and conditions: (a) Pd(PPh_3_)_4_, tributyl(vinyl)tin, toluene, reflux, 20–22 h, yields 82–86%. (b) 2,6-Dibromopyridine **3**, Pd(OAc)_2_, Bu_4_NBr, K_2_CO_3_, DMF, reflux, 24 h, yields 65–68%. (c) Pd(PPh_3_)_4_, tributyl(vinyl)tin, toluene, reflux, 20 h yields 75–80% (d) 2,6-dibromopyridine 3, Pd(OAc)_2_, Bu_4_NBr, K_2_CO_3_, DMF, reflux, 24 h, yields 62–70%.

New photoactive conjugates were prepared using different reactions: cross-coupling reactions, such as the Stille and Heck, condensation reactions, such as the Knoevenagel and Wadsworth–Horner–Emmons, and the 1,3-dipolar cycloaddition reaction. All reactions were conducted under argon and Schlenk conditions. The solvents were first dried by standard procedures such as sodium/benzophenone or CaH_2_ and freshly distilled before use. Pyridine-vinylene spacers of different lengths **2–6a** and **2–6b** were synthesized by successive Stille and Heck cross-coupling reactions starting with 6-bromo-2-pyridine-carboxaldehyde **1a**, 6-bromo-3-pyridinecarboxaldehyde **1b** and 2,6-dibromopyridine **3** as the main building blocks. The synthetic procedures are shown in Scheme 1. Monomers **2a** and **2b** were prepared using Stille reactions starting from the corresponding bromo-pyridinecarboxaldehydes **1a** or **1b** and tributyl(vinyl)tin using Pd(PPh_3_)_4_ as catalyst and anhydrous toluene as solvent. These monomers were treated with 2,6-dibromopyridine **3** under palladium-catalyzed Heck coupling conditions to give intermediates **4a** and **4b**, which were subsequently converted to the desired dimers **5a** and **5b** *via* Stille cross-coupling reactions in anhydrous toluene using Pd(PPh_3_)_4_ as catalyst. Finally, **6a** and **6b** were synthesized starting from the previously obtained monomers **2a,b** by a Pd(OAc)_2_ catalyzed double Heck reaction with 2,6-dibromopyridine **3** in dimethylformamide (DMF). For these reactions it was necessary to use an excess of the monomers (2.5 eq.).

**Scheme 2:**
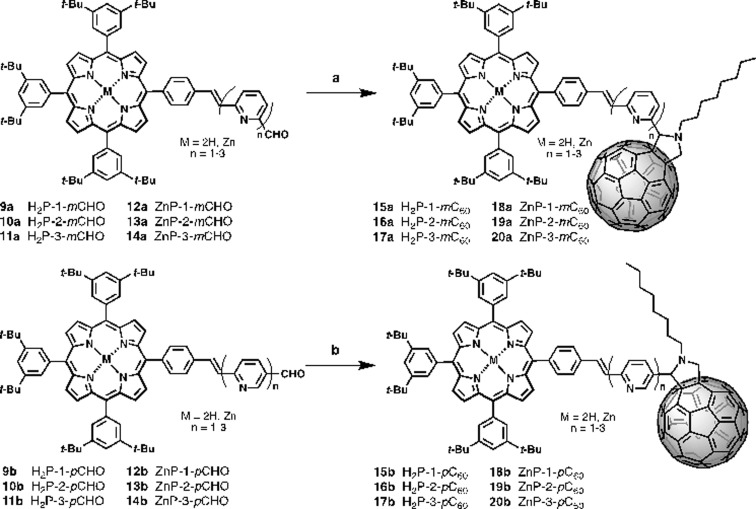
Synthesis of new porphyrin–fullerene conjugates: top **15–20a** (H_2_P-*n-m*C_60_, ZnP-*n-m*C_60_, *n* = 1-3); bottom **15–20b** (H_2_P-*n-p*C_60_, ZnP-*n-p*C_60_, *n* = 1–3). Reagents and conditions: (a) C_60_, N-octylglycine, toluene, reflux, 4–5 h, yields (**15a**, 32%); (**16a**, 35%); (**17a**, 30%); (**18a**, 38%); (**19a**, 34%); (**20a**, 36%); (b) C_60_, N-octylglycine, toluene, reflux, 4–5 h, yields (**15b**, 31%); (**16b**, 38%); (**17b**, 37%); (**18b**, 34%); (**19b**, 45%); (**20b**, 42%).

The synthetic routes for the preparation of **15–20a** and **15–20b** involved consecutive multistep procedures, as shown in Scheme 2. In particular, intermediates **9–10a,b** (H_2_P-*n-m*CHO, H_2_P-*n-p*CHO, *n* = 1–2) and **12–13a,b** (ZnP-*n-m*CHO, ZnP-*n-p*CHO, *n* = 1–2) were obtained by coupling of porphyrin precursors **7a,b** and pyridine-vinylene linkers **2a,b** and **5a,b** using DMF as solvent and Pd(OAc)_2_ as catalyst (see ESI, Scheme S1†). The porphyrin precursors **7a,b** and **8a,b** were prepared according to previous reports.^56–59^ The intermediates **11a,b** (H_2_P-3-*m*CHO, H_2_P-3-*p*CHO) and **14a,b** (ZnP-3-*m*CHO, ZnP-3-*p*CHO) were synthesized using Wadsworth–Horner–Emmons reactions between pyridine-vinylene linkers **6a,b** and **8a,b** using tetrahydrofuran as solvent (see ESI, Scheme S2†). Finally, the 1,3-dipolar cycloaddition reaction^60,61^ of azomethine ylides generated *in situ* in refluxing anhydrous toluene from **9–14a** (H_2_P-*n-m*CHO, ZnP-*n-m*CHO, *n* = 1–3), **9–14b** (H_2_P-*n-p*CHO, ZnP-*n-p*CHO), and *N*-octylglycine to C_60_ afforded the desired compounds **15–20a** (H_2_P-*n-m*C_60_, ZnP-*n-m*C_60_, *n* = 1–3) and **15–20b** (H_2_P-*n-p*C_60_, ZnP-*n-p*C_60_, *n* = 1–3) in 30–42% yields (see the ESI† for details).

The structures of all the new conjugates were confirmed unambiguously by analytical measurements and spectroscopic techniques including ^1^H-NMR, ^13^C-NMR, FT-IR, and MALDI-TOF mass spectrometry. The ^1^H NMR spectra of **15–20a** (H_2_P-*n-m*C_60_, ZnP-*n-m*C_60_, *n* = 1–3) and **15–20b** (H_2_P-*n-p*C_60_, ZnP-*n-p*C_60_, *n* = 1–3) exhibit the characteristic ^1^H-NMR pattern for 2-substituted pyrrolidines with aromatic proton signals due to the porphyrins and pyridine-vinylene spacers. The ^13^C-NMR spectral data are also in good agreement with the formulated structures. The presence of all the structures was corroborated by MALDI-TOF mass spectrometry, which showed the molecular-ion peak [M + H]^+^ and the fragment after the loss of C_60_ [M – C_60_]^+^ (see ESI†).

### Ground-state interactions

#### Electrochemistry

**Fig. 1:**
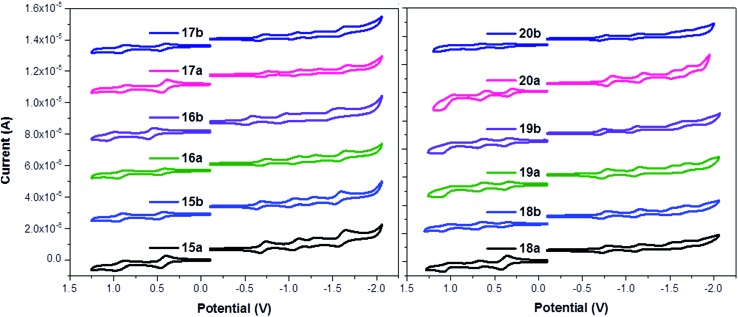
Left: CVs for porphyrin–fullerene conjugates **15–17a** (H_2_P-*n-m*C_60_, *n* = 1–3) and **15–17b** (H_2_P-*n-m*C_60_, *n* = 1–3) in DCM solutions (0.1 M TBAPF_6_) at room temperature. Right: CVs for porphyrin–fullerene conjugates **18–20a** (ZnP-*n-p*C_60_, *n* = 1–3) and **18–20b** (ZnP-*n-p*C_60_, *n* = 1–3) in DCM solutions (0.1 M TBAPF_6_) at room temperature. Oxidative scans between 0 and 1.25 V; reductive scans between 0 and –2.10 V.

The redox properties of the new porphyrin–fullerene conjugates were studied to probe the electronic coupling between the electron donor and the acceptor in the ground state through each molecular pyridine-vinylene linker. The electrochemistry of D–π–A conjugates **15–20a** (H_2_P-*n-m*C_60_, ZnP-*n-m*C_60_) and **15–20b** (H_2_P-*n-p*C_60_, ZnP-*n-p*C_60_) was studied by cyclic and differential pulse voltammetry (Fig. 1 and S1†) at room temperature in dry dichloromethane (DCM) solutions containing tetra-*n*-butylammonium hexafluorophosphate (TBAPF_6_ 0.1 M) as supporting electrolyte. A glassy carbon electrode was used as the working electrode, an Ag-wire as reference and a Pt-wire as the counter-electrode. The redox potentials are referenced to the internal ferrocene/ferrocenium couple (Fc/Fc^+^). The corresponding redox potentials are summarized in Table S1† along with those for C_60_ used as reference for comparison. All the systems show oxidation waves between +0.271 and +0.692 V corresponding to the oxidation processes of the H_2_P and ZnP, and reversible waves between –1.125 and –2.259 V due to reduction processes of both the fullerene and porphyrin fragments (Fig. 1).

Fig. 1 shows the cyclic voltammograms of the porphyrin–fullerene conjugates **15–20a** (H_2_P-*n-m*C_60_, ZnP-*n-m*C_60_, *n* = 1–3) and **15–20b** (H_2_P-*n-p*C_60_, ZnP-*n-p*C_60_, *n* = 1–3). In the reductive scan, each conjugate shows the reduction profile of four or five one-electron reversible reduction waves, respectively, corresponding to C_60_ and porphyrin centred processes. The first, second, and fourth reductions can be assigned to the fulleropyrrolidine-centred processes by comparison with C_60_. The third and fifth reduction waves are centred on the porphyrin subunit with Zn **18–20a** (ZnP-*n-m*C_60_, *n* = 1–3), **18–20b** (ZnP-*n-p*C_60_, *n* = 1–3) and without Zn **15–17a** (H_2_P-*n-m*C_60_, *n* = 1–3), **15–17b** (H_2_P-*n-p*C_60_, *n* = 1–3). The reduction potentials for the new electroactive conjugates are cathodically shifted relative to the values for pristine C_60_, as expected for a monofunctionalized C_60_.^28,62^

The oxidative scans show the first and second one-electron reversible oxidation waves corresponding to H_2_P and ZnP centred processes. H_2_P derivatives **15–17a** (H_2_P-*n-m*C_60_, *n* = 1–3), **15–17b** (H_2_P-*n-p*C_60_, *n* = 1–3) exhibit one oxidation wave, while the ZnP derivatives **18–20a** (ZnP-*n-m*C_60_, *n* = 1–3) and **18–20b** (ZnP-*n-p*C_60_, *n* = 1–3) feature two oxidation waves at potentials similar to those observed for the reference tetraphenylporphyrin (TPP).^50,52^

The electron donor ability of H_2_P and ZnP within electroactive systems was confirmed by the remarkably low value of their first oxidation potential, between +0.27 and +0.48 V, similar to those found for other compounds. For the reduction processes (shown in Table S1†) the first reduction potential is shifted cathodically by 110–148 mV compared to the first reduction potential of C_60_, which shows the strong *push–pull* nature of the electroactive species, allowing the prediction of the formation of a charge-separated state by photoexcitation. The reduction potentials of each series show that the shifts for the different molecular linker lengths are not significant. However, the isomeric 1,3 or 1,4-disubstituted systems (*meta* or *para*), do exhibit differences of ∼20 to 27 mV in the cathodic shifts. The 1,4-disubstituted systems **15–20b** (H_2_P-*n-p*C_60_, ZnP-*n-p*C_60_) display first reduction potentials that are less negative than their 1,3-disubstituted counterparts **15–20a** (H_2_P-*n-m*C_60_, ZnP-*n-m*C_60_).

#### Absorption spectroscopy

**Fig. 2:**
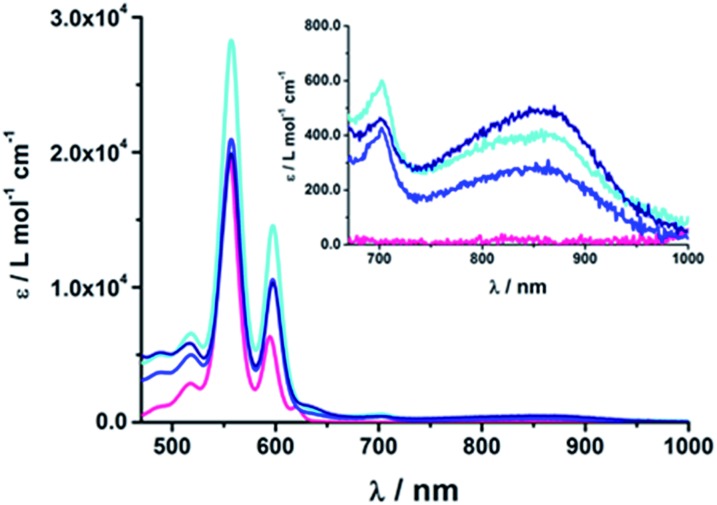
Absorption spectra of (**18b**) ZnP-1-*p*C_60_ (cyan); (**19b**) ZnP-2-*p*C_60_ (blue); (**20b**) ZnP-3-*p*C_60_ (navy) and ZnTPP (pink) as the reference in THF at room temperature.

The optical UV-Vis absorption spectra of the electron donor–acceptor conjugates exhibited the contributions and features of their components, pyridine-vinylene linkers, C_60_, H_2_P, and ZnP, as shown in Fig. S2 and S3.† The absorption spectra of the H_2_P-based conjugates **15–17a** and **15–17b** exhibit strong maxima at around 421 nm in addition to four weaker absorption bands in the range from 500 to 700 nm, corresponding to the H_2_P Soret- and Q-band absorptions, respectively. Compared to the H_2_TPP reference, the Soret bands are red shifted by about 5 nm, while the Q bands exhibit redshifts between 1 and 4 nm. In contrast, ZnP-containing conjugates **18–20a** and **18–20b** exhibit absorptions at 427 nm compared to 423 nm for ZnTPP and only two Q bands at 557 and 597 nm, 2–3 nm red shifted compared to ZnTPP. Furthermore, a rather broad, though weak, absorption evolves between 300 and 400 nm, which is assigned to C_60_. The characteristic absorption of the mono-functionalized C_60_ that usually appears at 430 nm overlaps with the stronger absorptions of the porphyrin fragments. These shifts and the fact that the extinction coefficients are comparable but not identical to those observed for the references, suggests significant electronic interactions between the individual components in the ground state. In this context, we focused on the low-energy region of the absorption spectra, as shown in Fig. 2. In this instance, features whose origins are neither linked to C_60_ nor to pyridine-vinylene or H_2_P/ZnP are discernable. A weak maximum is seen at around 860 nm that is assigned to a charge-transfer transition.^63–66^ Within the ZnP-containing conjugates, **20b** (ZnP-3-*p*C_60_) exhibits the strongest charge-transfer band and **18b** (ZnP-1-*p*C_60_) and **19b** (ZnP-2-*p*C_60_) show weaker interactions, with electronic coupling elements of 400 and ∼100 cm^–1^, respectively. Weaker and energetically shifted interactions for H_2_P-containing conjugates with electronic coupling elements of 20–40 cm^–1^ preclude a meaningful analysis.

At first glance, comparing the absorptions of the *meta* and *para* linked pyridine–C_60_ conjugates revealed no particular differences. The more pyridine-vinylene groups are present in the linker, the higher the extinction coefficient between 300 and 400 nm becomes. Thus, the increased absorption in this wavelength range is attributed to the linker.

### Excited-state interactions

To gain further insights into the excited-state interactions, both steady state and time-resolved emission spectroscopy (time correlated single photon counting, TCSPC) and time-resolved absorption spectroscopy (transient absorption) were employed.

#### Steady-state fluorescence

**Fig. 3:**
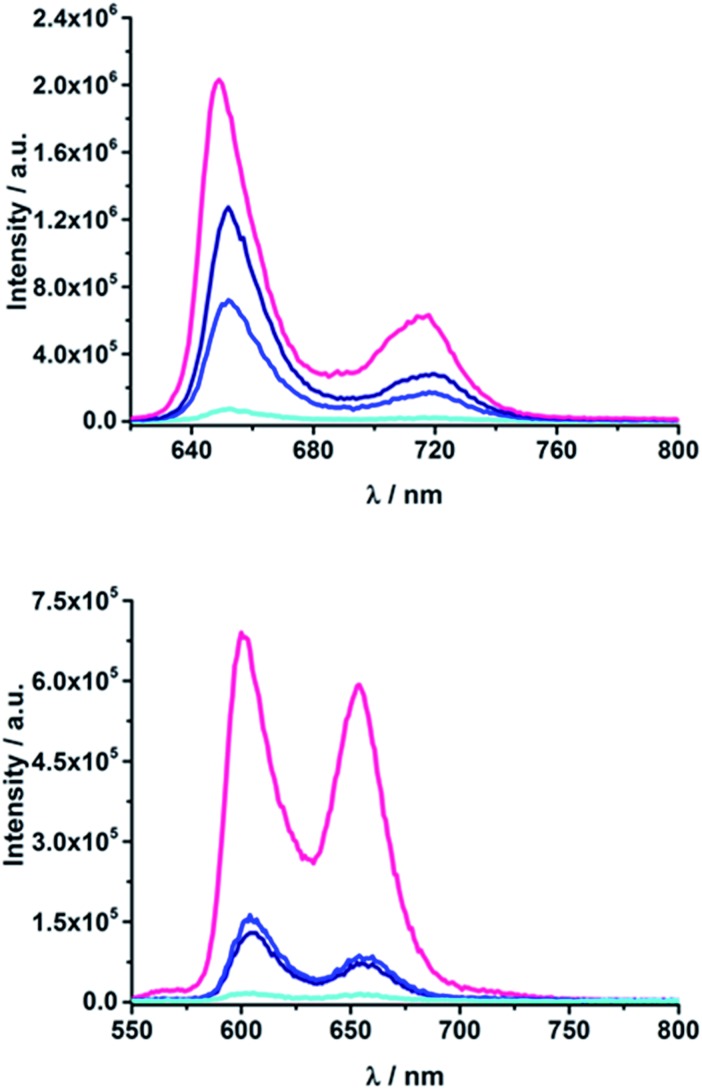
Above: Fluorescence spectra of (**15a**) H_2_P-1-*m*C_60_ (cyan); (**16a**) H_2_P-2-*m*C_60_ (blue); (**17a**) H_2_P-3-*m*C_60_ (navy) and H_2_TPP (pink) (excitation at 420 nm; OD = 0.04) in THF at room temperature. Below: Fluorescence spectra of (**18a**) ZnP-1-*m*C_60_ (cyan); (**19a**) ZnP-2-*m*C_60_ (blue); (**20a**) ZnP-3-*m*C_60_ (navy) and ZnTPP (pink) (excitation at 420 nm; OD = 0.05) in THF at room temperature.

Upon excitation of H_2_TTP at 420 nm, characteristic fluorescence maxima at 650 and 720 nm are discernable, while ZnTTP exhibits maxima at 605 and 655 nm, as shown in Fig. 3 and S4 in the ESI.† The values for the fluorescence maxima of the electron donor–acceptor conjugates are listed in Table 1. Notably, the fluorescence maxima of the different electron donor–acceptor conjugates and their references that lack C_60_ evolve at the same wavelength. Compared to the references H_2_TPP and ZnTPP, they are shifted 3–4 nm to the red. Furthermore, red shifts were found when emission spectra of electron donor–acceptor conjugates were obtained in solvents with higher polarity, such as benzonitrile. These red shifts of the bands have been assigned for other D–π–A systems to an intramolecular electron transfer process in the excited state between the porphyrin and C_60_.^67^ References **9–14a,b** exhibit higher fluorescence quantum yields than H_2_TPP and ZnTPP – 0.15 *versus* 0.10 and 0.065 *versus* 0.040.^68,69^ Thus, it can be assumed that at 420 nm the pyridine-vinylene linker is also excited and transfers energy to the porphyrin. More importantly, for the electron donor–acceptor conjugates, the fluorescence intensity decreases as the linker length decreases. This observation is quantified by the fluorescence quantum yields (*Φ*_F_) shown in Table 1. Consequently, **18a** (ZnP-1-*m*C_60_) and **18b** (ZnP-1-*p*C_60_) show the lowest fluorescence quantum yields. With values of 4.6 × 10^–4^ and 3.7 × 10^–4^ in THF, their ZnP fluorescence is almost completely quenched. **15a** (H_2_P-1-*m*C_60_) and **15b** (H_2_P-1-*p*C_60_) exhibit the lowest fluorescence quantum yields among the H_2_P-containing systems with values of 0.003 and 0.007 in THF. In contrast, **17b** (H_2_P-3-*p*C_60_) and **20b** (ZnP-3-*p*C_60_) feature values of 0.10 and 0.04, which are similar to those seen for H_2_TPP and ZnTPP. Compared to **11b**, (H_2_P-3-*p*CHO) and **14b** (ZnP-3-*p*CHO), their fluorescence is quenched by about 30%. Additionally, *Φ*_F_ depends on the solvent polarity. In the more polar solvents, the fluorescence is more efficiently quenched than in the less polar solvents. When comparing the *meta*-substituted conjugates to the *para* substituted ones, the latter exhibit slightly higher quantum yields that the former. Since fluorescence quenching is only observed for the electron donor–acceptor conjugates and not for the references that lack C_60_, the quenching is attributed to an energy and/or electron transfer involving the porphyrin and C_60_.

**Table 1:** Room temperature fluorescence features.

Compound	*λ*_max_ (nm)			*Φ*_F_			*τ* (ns)		
	Tol	THF	PhCN	Tol	THF	PhCN	Tol	THF	PhCN
H_2_TPP	650	649	651		0.10			10.0	
ZnTPP	605	601	607		0.040			2.0	
**15a**	653	652	655	0.008	0.003	0.002	0.5	0.22	0.16
**15b**	653	652	655	0.014	0.007	0.005	0.8	0.27	0.26
**16a**	653	652	655	0.036	0.029	0.026	2.3	1.9	1.7
**16b**	653	652	655	0.061	0.051	0.047	3.4	3.0	3.0
**17a**	653	652	655	0.064	0.048	0.040	5.6	5.8	6.0
**17b**	653	652	655	0.112	0.099	0.095	7.3	7.1	7.1
**18a**	602	601	610	2 × 10^–4^	4.6 × 10^–4^	1 × 10^–4^	<0.15	<0.15	<0.15
**18b**	608	604	610	2.6 × 10^–4^	3.7 × 10^–4^	4.2 × 10^–4^	<0.15	<0.15	<0.15
**19a**	610	604	610	0.009	0.008	0.005	0.4	0.3	0.8
**19b**	611	604	611	0.014	0.014	0.012	0.6	0.5	0.5
**20a**	610	604	610	0.025	0.006	0.013	0.8	0.3	0.7
**20b**	610	604	612	0.038	0.043	0.034	1.2	1.2	1.2

#### Time-resolved emission

TCSPC experiments confirmed the trend observed from the steady-state emission measurements. On one hand, the fluorescence lifetimes correlate with the length of the linker; longer linkers result in longer lifetimes. On the other hand, the regiochemistry of the linker plays a decisive role, with the *para*-substituted systems showing slightly longer lifetimes than the *meta*-substituted ones. Furthermore, the solvent polarity influences the fluorescence lifetimes; less polar solvents result in longer lifetimes. Consequently, the longest lifetime of 7.3 ns was found for **17b** (H_2_P-3-*p*C_60_) in toluene, while **18a** (ZnP-1-*m*C_60_) and **18b** (ZnP-1-*p*C_60_) exhibit lifetimes shorter than the time resolution of our TCSPC setup – Table 1. References **9–14a,b** exhibited lifetimes of 10 and 2 ns, respectively, comparable to the values for H_2_TPP and ZnTPP.^70,71^ These results confirm the conclusions drawn from the steady-state fluorescence experiments indicating that an energy and/or electron transfer takes place between H_2_P/ZnP and C_60_.

#### Transient absorption spectroscopy

Transient absorption measurements were conducted in solvents of different polarity (toluene, THF, and PhCN) using two different setups in order to investigate the formation and deactivation processes of excited states upon photoexcitation of the conjugates and the corresponding reference compounds. To investigate processes in the ps/ns time scale (up to 7.5 ns), the sample was excited with a 150 fs laser pulse at either 387 nm (200 nJ; *c* = 10^–5^ M) or 420 nm (200 nJ; *c* = 10^–6^ M) using the Helios spectrometer. To follow processes on the ns/μs/ms time scale, the EOS spectrometer was employed, exciting at 387 nm (1 μJ, *c* = 10^–5^ M) with time scales up to 400 μs. Transient absorptions were also investigated by exciting at either 355 nm (6 ns laser pulse, 10 mJ, *c* = 10^–5^ M) or 420 nm (3 ns laser pulse, 5 mJ, *c* = 10^–6^ M) using time scales up to 1 ms.

**Fig. 4:**
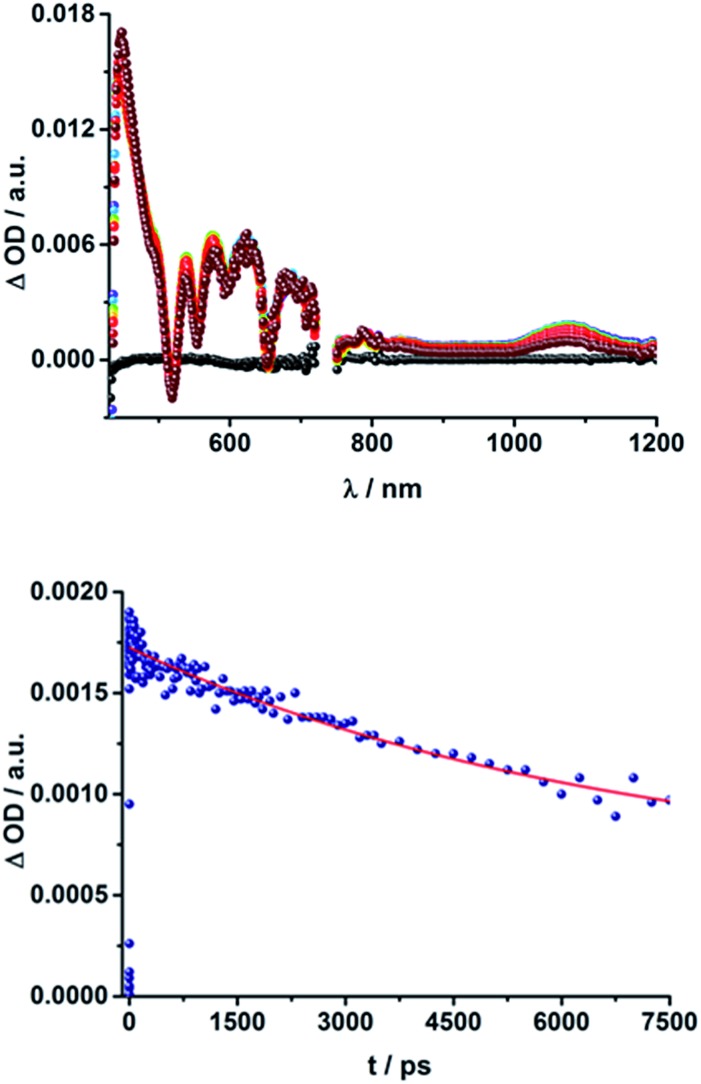
Above: Differential absorption spectra (visible and near-infrared) observed upon femtosecond flash photolysis (420 nm, 150 nJ) of **9a** (H_2_P-1-*m*CHO) in THF with time delays between 0 ps (black) and 7.5 ns (wine) at room temperature. Below: Time-absorption profile of the spectra above at 1070 nm, monitoring the deactivation of the porphyrin singlet excited state.

Upon excitation at 387 and 420 nm, the differential absorption spectra of **9–11a** (H_2_P-*n-m*CHO, *n* = 1–3) and **9–11b** (H_2_P-*n-p*CHO, *n* = 1–3) are dominated by features of the H_2_P singlet excited state,^71,72^ as shown in Fig. 4, left. This state is formed immediately upon excitation and exhibits maxima at 450, 540, 575, 630, and 670 nm in addition to a broad transient absorption between 1000 and 1150 nm. Additionally observed ground-state bleaching at 420, 520, 550, 590, and 650 nm correspond to the Soret- and Q-band absorptions of H_2_P, respectively. The porphyrin's singlet excited state is stable during the time scale of our fs transient-absorption setup (7.5 ns, see Fig. 4). The differential absorption spectra of all H_2_P based references look nearly identical and also resemble the transient absorption spectra of H_2_TTP closely. Thus, it can be assumed that the pyridine-vinylene linker does not show transients upon photoirradiation. The ZnP-pyridine-vinylene reference compounds give similar results, as shown in Fig. S5.† As for ZnTPP, upon 420 nm excitation the ^1^*ZnP^70,72^ state dominates the visible region of the differential absorption spectrum with maxima at 460, 580, and 630 nm and minima at 560 and 600 nm (ground-state bleaching). The singlet excited porphyrin then undergoes intersystem crossing within 2 ns to give the ^3^*ZnP, which is stable within the 7.5 ns time scale of the system and shows maxima at 480 and 840 nm.

**Fig. 5:**
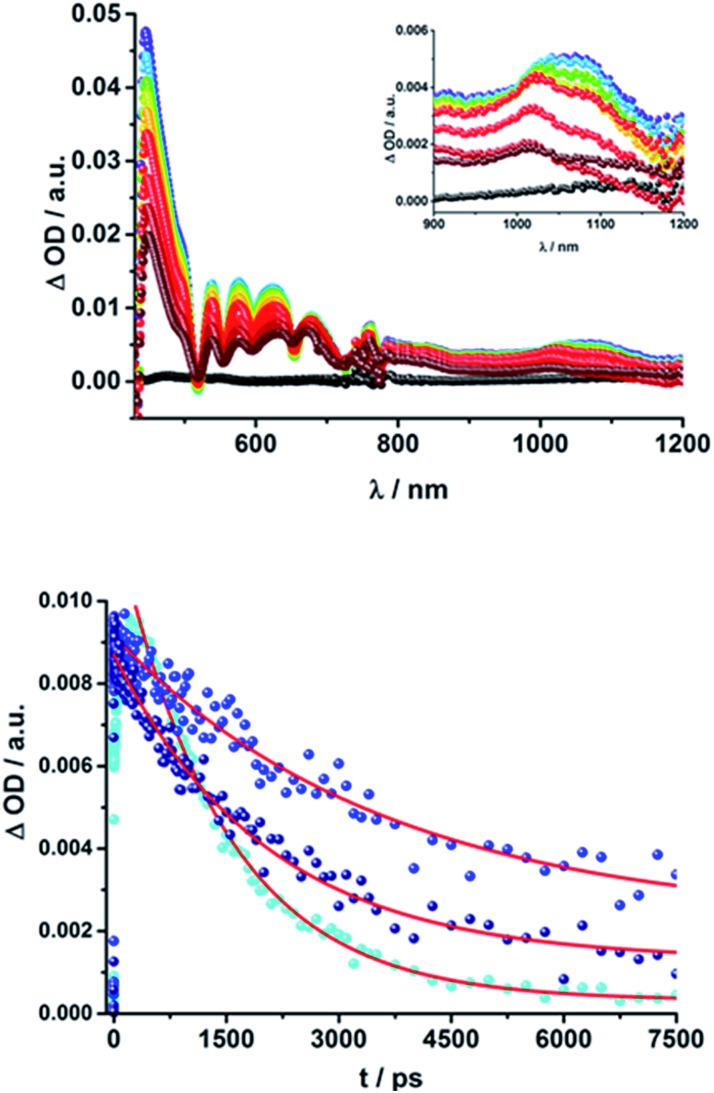
Above: Differential absorption spectra (visible and near-infrared) observed upon femtosecond flash photolysis (387 nm, 200 nJ) of **16a** (H_2_P-2-*m*C_60_) in THF with time delays between 0 ps (black) and 7.5 ns (wine) at room temperature. Below: Time-absorption profiles of the 1010 nm decay for (**15a**) H_2_P-1-*m*C_60_ (cyan); (**16a**) H_2_P-2-*m*C_60_ (blue) and (**17a**) H_2_P-3-*m*C_60_ (navy) upon femtosecond flash photolysis (387 nm, 200 nJ) in THF at room temperature, monitoring the charge recombination.

When exciting the H_2_P–C_60_ electron donor–acceptor conjugates with a fs-laser pulse at 387 and 420 nm, respectively, the most prominent features of the transient absorption spectra are in the visible region, as seen for the references, those belonging to the H_2_P singlet excited state, (maxima at ∼450, 540, 575, 630, and 670 nm) and ground-state bleaching at ∼420, 520, 550, 590, and 650 nm; a representative example is shown Fig. 5 and S6 in the ESI.† The features of the ^1^*H_2_P, with a broad maximum between 1000 and 1150 nm, can be observed in the NIR region. However, in contrast to the references, the singlet excited state of the porphyrin is shorter lived in the presence of C_60_. **15a** (H_2_P-1-*m*C_60_) and **15b** (H_2_P-1-*p*C_60_) exhibit the shortest singlet state lifetimes (hundreds of picoseconds in THF). With increasing length of the linker, the decay of the singlet excited state of the porphyrin becomes slower. In **17b** (H_2_P-3-*p*C_60_) the ^1^*H_2_P lifetime even exceeds the time scale of our fs setup, as observed for the reference systems that lack C_60_ (compare Fig. S6†).

These results also confirm those from the steady-state and time-resolved emission experiments. Furthermore, additional features corresponding to the C_60_ singlet excited state are observed upon 387 nm excitation, as a 920 nm absorption maximum. The transient characteristics, which correlate with the triplet excited state of C_60_ at 690 nm are, however, masked by the more intense H_2_P transients and thus barely visible. Finally, a distinct peak arises in the NIR region of the differential absorption spectra of **15a** (H_2_P-1-*m*C_60_) and **15b** (H_2_P-1-*p*C_60_) (Fig. S6†) with a maximum at ∼1010 nm. This feature is assigned to the singly reduced fullerene's fingerprint, which is well known from the literature.^24,73^ Although this signal coincides with the singlet features of H_2_P, it can be clearly distinguished, since they exhibit comparably short-lived singlets. For **16a** (H_2_P-2-*m*C_60_) (Fig. 5), **17a** (H_2_P-3-*m*C_60_) and **16b** (H_2_P-2-*p*C_60_) (Fig. S6†) the fullerene anion fingerprint cannot be clearly identified in the differential absorption spectra because of the increased signal of the ^1^*H_2_P. However, upon closer examination of the NIR region (Fig. 5, above, and Fig. S6†) an individual peak can be discerned at ∼1010 nm. In contrast, for **17b** (H_2_P-3-*p*C_60_), the singly-reduced C_60_ cannot be identified unambiguously, since it is masked by the rather long lived ^1^*H_2_P. Therefore, we cannot rule out the formation of a CSS for the latter.

**Fig. 6:**
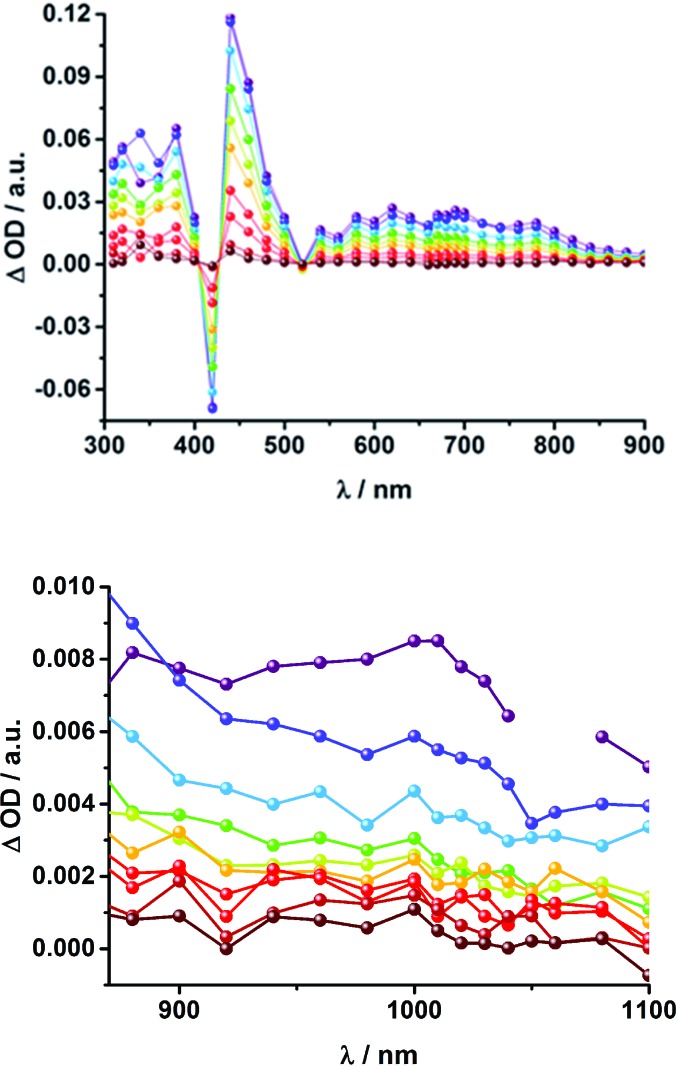
Above: Differential absorption spectra (visible) observed upon nanosecond flash photolysis (355 nm, 10 mJ) of **16a** (H_2_P-2-*m*C_60_) in THF with time delays between 150 ns (purple) and 2.0 μs (wine) at room temperature under aerobic conditions. Below: Differential absorption spectra (near-infrared) observed upon nanosecond flash photolysis (355 nm, 10 mJ) of **16a** (H_2_P-2-*m*C_60_) in THF with time delays between 60 ns (purple) and 2.0 μs (wine) at room temperature under aerobic conditions.

**Fig. 7:**
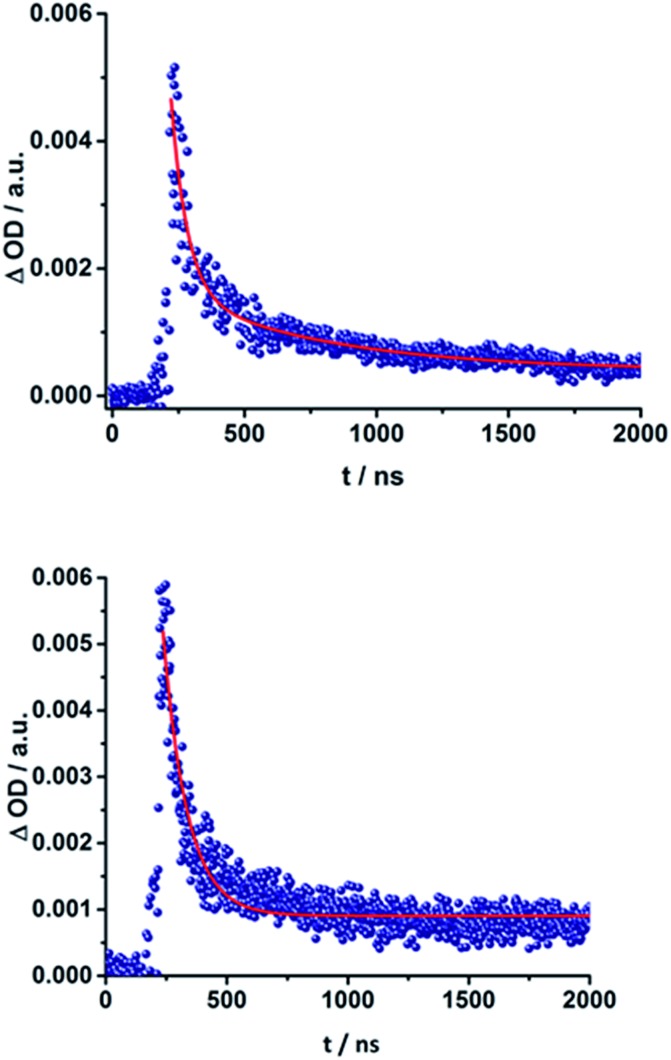
Above: Time-absorption profile of the 1010 nm decay – Fig. 6 – of **16a** (H_2_P-2-*m*C_60_) upon nanosecond flash photolysis (355 nm, 10 mJ) in THF under aerobic conditions, monitoring the charge recombination process. Below: Time-absorption profile of the 1010 nm decay of **16b** (H_2_P-2-*p*C_60_) upon nanosecond flash photolysis (355 nm, 10 mJ) in THF under aerobic conditions, monitoring the charge recombination.

The presence of the C_60_ anion signature in the transient absorption spectra proves that charge transfer takes place in the conjugates. The associated radical cation transient absorption again overlaps with the porphyrin signatures. However, it can be probed by analyzing the decay kinetics. Even though the triads show the same transients initially, the transient absorption spectra show considerably different lifetimes of their excited states. As discussed above, the singlet excited-state lifetimes increase with the length of the linker. Furthermore, the latter also decrease with solvent polarity, as shown in Table 1. Not only the lifetimes of the singlet excited state vary with the length of the linker and with the solvent polarity; those of the radical ion pair state also do. From a multi-wavelength analysis of the decays of the C_60_ radical anion and of the H_2_P radical cation, a lifetime of 1.4 ns was determined for **15a** (H_2_P-1-*m*C_60_) in THF. The lifetime of the radical ion pair state changed on varying the solvent. In toluene, for example, a lifetime of 2.0 ns was found for **15a** (H_2_P-1-*m*C_60_), while in PhCN the lifetime was only 662 ps, as shown in Table S2 of the ESI.† Even more distinct differences in the radical ion pair lifetime are observed, when different linker lengths are considered, as shown in Fig. 5 (below) for kinetic measurements. Ongoing from one pyridine-vinylene group in **15a** (H_2_P-1-*m*C_60_) to two in **16a** (H_2_P-2-*m*C_60_), the lifetime increases in THF. Because the 1010 nm decay is not complete for **16a** (H_2_P-2-*m*C_60_) within the timescale of 7.5 ns, we turned to EOS fs-measurements and nanosecond transient absorption spectroscopy. Upon excitation of **16a** (H_2_P-2-*m*C_60_) with ns laser pulses at either 355 or 420 nm under different conditions, the features of the H_2_P radical anion are discernable, but are superimposed with those of the C_60_ triplet excited state. The fingerprint of the C_60_ radical anion is clearly visible in the near-infrared region (Fig. 6, below). Its decay at 1010 nm, for example, (Fig. 7) fits by a single exponential function to afford a radical ion pair state lifetime of 35 ns for **16a** (H_2_P-2-*m*C_60_) in THF. Interestingly, the radical ion pair lifetime in **17a** (H_2_P-3-*m*C_60_) is 2.2 ns in THF, appreciably shorter than for **16a** (H_2_P-2-*m*C_60_) (Table 2). This behaviour will be discussed further later.^74^

**Table 2:** Charge separation (CS) and charge recombination (CR) of porphyrin–fullerene conjugates in THF at 298 K.

		CR	
	CS	fs-setup	ns-setup
**15a**	75 ± 3 ps	1.4 ± 0.1 ns	
**15b**	106 ± 2 ps	1.3 ± 0.1 ns	
**16a**	<1 ps	50 ± 3 ns	35 ± 9 ns
**16b**	<1 ps	58 ± 1 ns	65 ± 21 ns
**17a**	<1 ps	2.2 ± 0.1 ns	
**18a**	10 ± 0 ps	414 ± 2 ps	
**18b**	<1 ps	373 ± 1 ps	
**19a**	<1 ps	116 ± 19 ns	98 ± 24 ns
**19b**	<1 ps	79 ± 10 ns	165 ± 41 ns
**20a**	<1 ps	1.6 ± 0.1 ns	
**20b**	<1 ps	1.4 ± 0.1 ns	

**Fig. 8:**
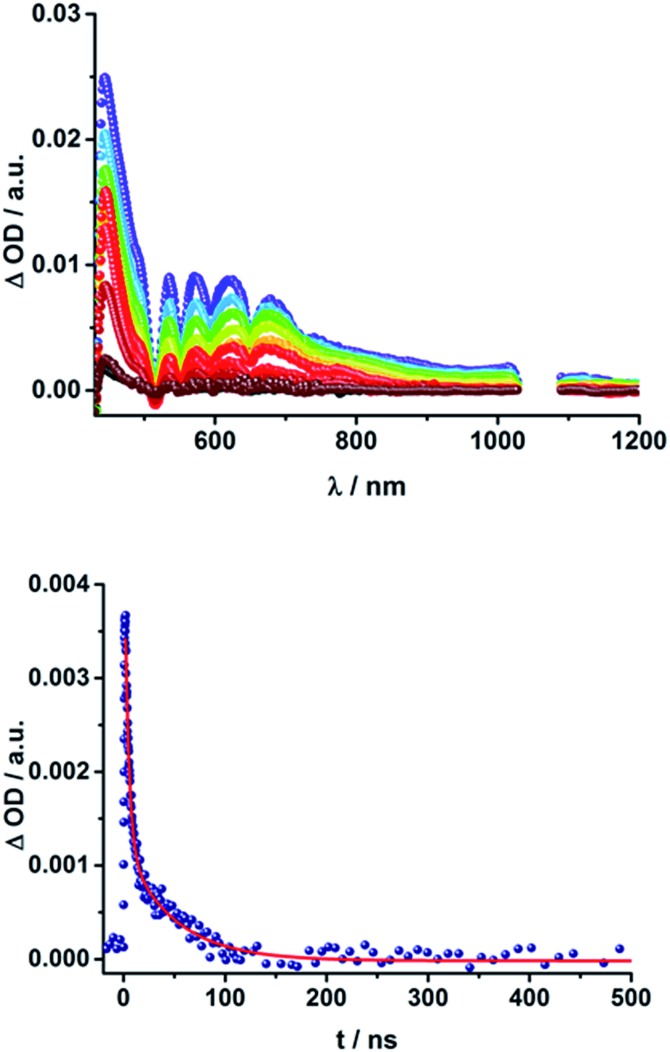
Above: Differential absorption spectra (visible and near-infrared) observed upon femtosecond flash photolysis (387 nm, 1 μJ) of **16a** (H_2_P-2-*m*C_60_) in THF with time delays between 0 ns (black) and 1.0 μs (wine) at room temperature under aerobic conditions. Below: Time-absorption profile of the spectra above at 1010 nm, monitoring the charge recombination.

When probing **16a** (H_2_P-2-*m*C_60_) in THF with the EOS fs-setup (Fig. 8, above), the visible region is again dominated by the porphyrin's triplet excited-state features, while the C_60_ radical anion 1010 nm fingerprint is discernible in the near infrared region. The 1010 nm decay (Fig. 8, below) is best fit by a biexponential function, affording a short-lived component of 4.3 ns attributable to an H_2_P-centered singlet excited state and a longer lived one of 50 ns assigned to a C_60˙_^–^ centered charge separation. By comparison with the ns transient absorption measurements, where the laser power is four orders of magnitude higher (1 μJ compared to 10 mJ), we conclude that charge recombination is independent of the applied laser power.

The same trend is observed for the radical ion pair state lifetimes for the *para* substituted conjugates, as shown in Table 2. While for **15b** (H_2_P-1-*p*C_60_) the radical ion pair state decays with a lifetime of 1.3 ns in THF, that of **16b** (H_2_P-2-*p*C_60_) does not decay within the time scale of our femtosecond setup. A lifetime of 65 ± 21 ns was determined for **16b** (H_2_P-2-*p*C_60_) in THF in complementary nanosecond experiments, as shown in Fig. 7. It was reassuring that EOS fs-measurements yielded a lifetime of 58 ns (Table 2).^75^ The formation of the charge-separated states was investigated in order to obtain further insight into the charge-transfer dynamics of the electron donor–acceptor conjugates. The time needed to form the charge separated state upon 387 nm excitation was determined from the rise of the signal corresponding to the fullerene anion, (Table 2). Clear trends can be observed for **15a** (H_2_P-1-*m*C_60_) and **15b** (H_2_P-1-*p*C_60_). The more polar the solvent, the faster the charge separation process occurs. Furthermore, the charge-separated state is formed more rapidly in **15a** (H_2_P-1-*m*C_60_) than for **15b** (H_2_P-1-*p*C_60_). For the systems with longer linkers, the electron is transferred to the fullerene in less than 1 ps, so that no further conclusions can be drawn from these results.

**Fig. 9:**
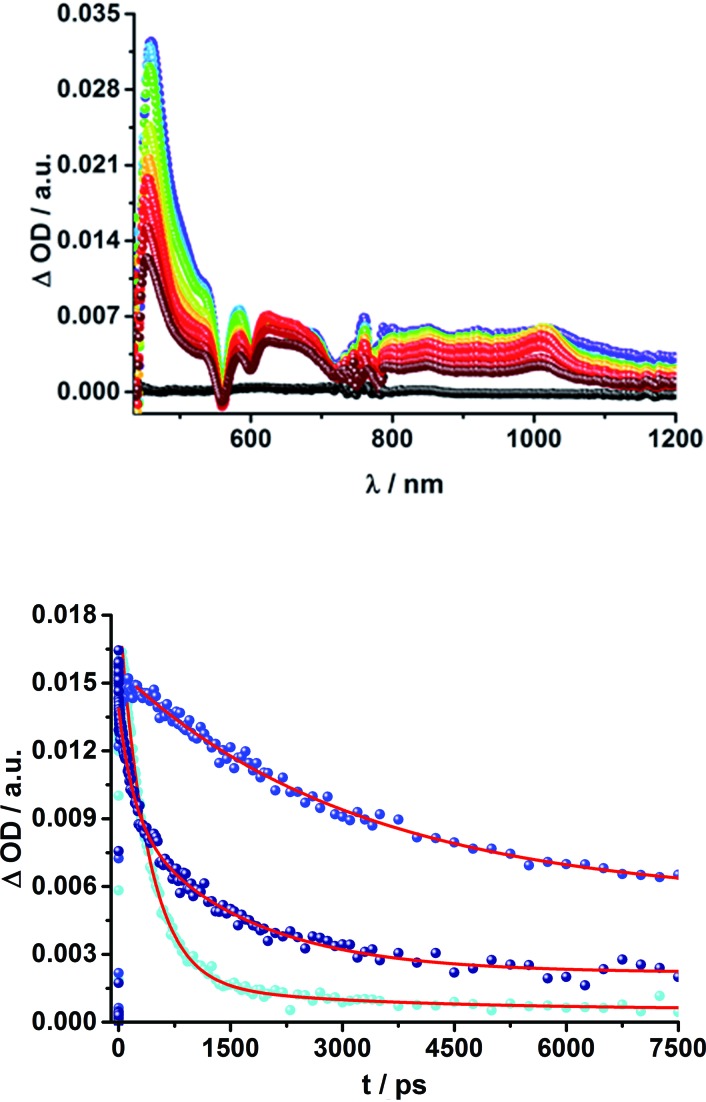
Above: Differential absorption spectra (visible and near-infrared) observed upon femtosecond flash photolysis (387 nm, 200 nJ) of **19a** (ZnP-2-*m*C_60_) in THF with time delays between 0 ps (black) and 7.5 ns (wine) at room temperature. Below: Time-absorption profiles of the 1010 nm decay for (**18a**) ZnP-1-*m*C_60_ (cyan); (**19a**) ZnP-2-*m*C_60_ (blue) and (**20a**) ZnP-3-*m*C_60_ (navy) upon femtosecond flash photolysis (387 nm, 200 nJ) in THF at room temperature, monitoring the charge recombination.

The C_60_ radical anion absorption at 1010 nm can be identified even more clearly for the ZnP–C_60_ D–A conjugates, since ZnP does not have transients in this region of the spectrum, as shown in Fig. 9 and S7 of the ESI.† Nevertheless, the visible region is again dominated by porphyrin features. To be more precise, maxima at 460, 580, and 620 nm and minima at 420, 560, and 600 nm evolve immediately after the 387 nm laser pulse. These correspond to the ZnP singlet excited state and ground state bleaching, respectively. While for **18a** (ZnP-1-*m*C_60_) ^1^*ZnP decays within 400 ps and only weak triplet signatures are discernible, the singlet lifetimes and the intensity of the ^3^*ZnP peaks (850 nm) increase with increasing length of the linker up to ∼1 ns for **20b** (ZnP-3-*p*C_60_). Additionally, at 920 nm a transient arises that can be assigned to ^1^*C_60_. The same trend as found for the H_2_P systems was observed for the charge-separated state lifetimes. The shortest CSS lifetime of the ZnP compounds was found for **18b** (ZnP-1-*p*C_60_) with ∼150 ps in PhCN, while **19a** (ZnP-2-*m*C_60_) and **19b** (ZnP-2-*p*C_60_) in THF and toluene do not decay within the 7.5 ns time scale of our fs-setup (Fig. 9, below).

Lifetimes of 98 ns for **19a** (ZnP-2-*m*C_60_) and 165 ns for **19b** (ZnP-2-*p*C_60_) in THF were determined in complementary ns experiments. In EOS experiments, however, slightly different lifetimes, 116 ns for **19a** (ZnP-2-*m*C_60_) and 79 ns for **19b** (ZnP-2-*p*C_60_), were determined. As described for the H_2_P-conjugates, the CSS lifetime decreases with increasing solvent polarity and the longest lifetimes were determined for the compounds with two pyridine-vinylene groups as linkers rather than of those with the longer linker. All CSS lifetimes determined from multi-wavelength fits either from fs or ns transient absorption experiments are summarized in Tables 2 and S2.†

Analysis of the charge-separation kinetics of the ZnP D–π–A conjugates did not yield a clear trend. For **18a** (ZnP-1-*m*C_60_) in THF and **18b** (ZnP-1-*p*C_60_) in toluene, CS takes place within ∼10 ps, while the CSS is formed within 2 ps for both in PhCN. CS is too fast to be monitored with our setup in all other ZnP compounds.

#### Electron spin resonance

**Fig. 10:**
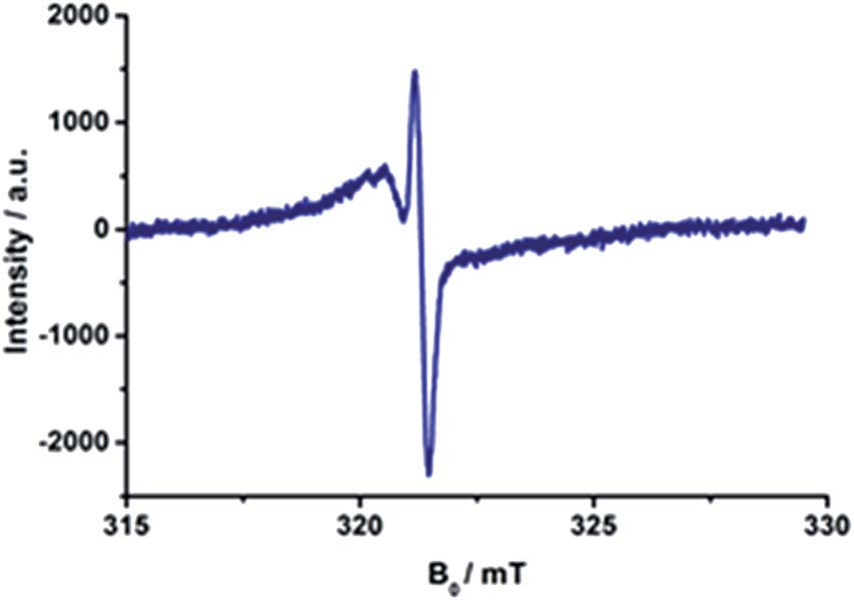
EPR signals observed under photoirradiation of **15a** (H_2_P-1-*m*C_60_) in benzonitrile at 77 K.

As a complement to the room temperature (298 K) measurements, charge separation was also probed at low temperature (77 K) by means of EPR measurements. Fig. 10 shows a typical example, where the two signals due to C_60_˙^–^ and H_2_P˙^+^ are discernable upon photoirradiation, at *g* = 2.0002 and 2.0026, respectively, related to the triplet charge-separated state. The rather sharp signal at *g* values smaller than that of the free spin value is diagnostic for the presence of pristine C_60_.^^82^^ Interestingly, the fine structure of the triplet charge-separated state is also observable at *g* = 4. Its amplitude is, however, rather weak due to the forbidden nature of the “Δ*M*_s_ = 2” transitions (see Fig. S7 in the ESI†). Similar triplet EPR signals were observed for the charge-separated states of the remaining porphyrin–C_60_ conjugates (Fig. S11 and S12 in the ESI†).

**Fig. 11:**
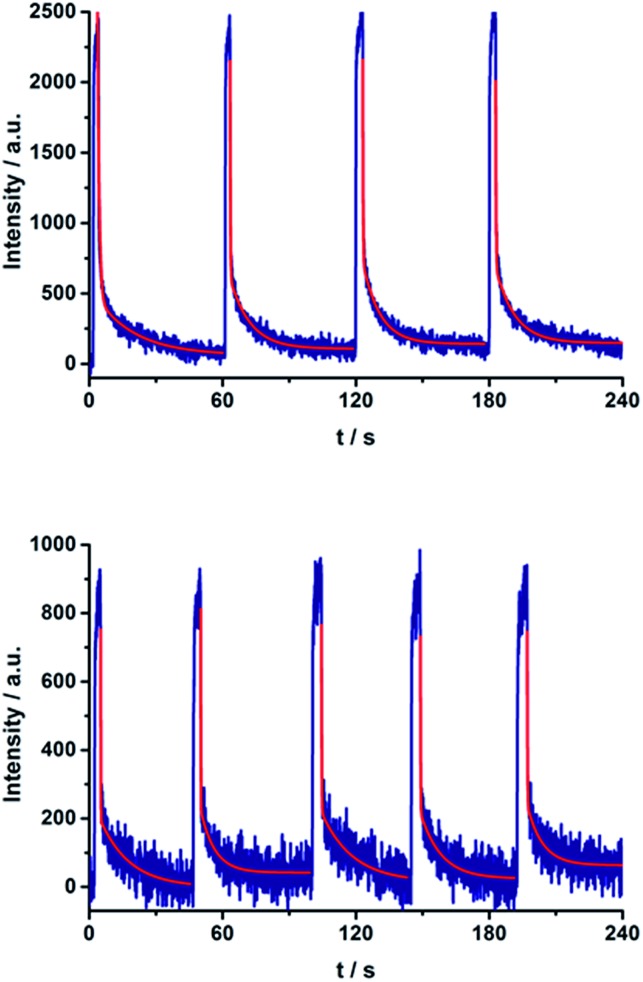
On–off switch of the EPR signal due to charge separation of **17a** (top) and **20a** (bottom) in PhCN at 77 K by turning on and off the irradiation from a high-pressure mercury lamp.

Repeated on–off switching of the charge-separated state formation was realized by turning on and off the irradiation source many times, see Fig. 11. The corresponding lifetimes at 77 K are long enough to be detected during the on–off cycling for both series of porphyrin–C_60_ conjugates (Fig. S13 and S14 in the ESI†). Table 3 lists the CS lifetimes determined from the EPR experiments at 77 K. Due to the time resolution of the available equipment, only lifetimes >200 ms could be detected.

**Table 3:** Charge recombination (CR) of porphyrin–fullerene conjugates in THF and PhCN at 77 K.

Compound	THF	PhCN
	CR	CR
**15a**	<200 ms	<200 ms
**15b**	<200 ms	<200 ms
**16a**	440 ± 60 ms	<200 ms
**16b**	420 ± 40 ms	220 ± 40 ms
**17a**	510 ± 130 ms	<200 ms
**17b**	<200 ms	240 ± 20 ms
**18a**	420 ± 40 ms	<200 ms
**18b**	260 ± 60 ms	<200 ms
**19a**	<200 ms	<200 ms
**19b**	<200 ms	240 ± 30 ms
**20a**	<200 ms	<200 ms
**20b**	270 ± 30 ms	260 ± 30 ms

### Molecular modelling

We turned to molecular modelling to investigate the fact that the D–A conjugates with the longest linker do not exhibit the longest lived CSS. Conformational analysis of the free base molecules was performed with the Conformer program^76^ to determine average electron donor–acceptor distances. 10 000 conformations were determined for each molecule *via* a Metropolis Monte-Carlo algorithm. Each conformation was optimized with the COMPASS force field.^77^

**Fig. 12:**
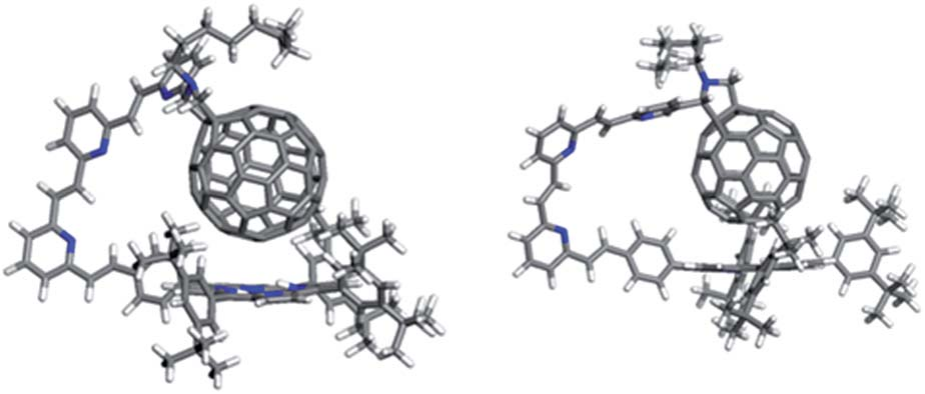
Lowest-energy conformations of (**17a**) H_2_P-3-*m*C_60_ (left) and (**17b**) H_2_P-3-*p*C_60_ (right).

The D–A distance for the lowest-energy conformer and the mean distances (including standard deviations) for all conformers within 20 kcal mol^–1^ of the lowest-energy conformation are given in Table 4. For both the *meta*- and *para*- series, the longest molecules do not follow the trend of increased D–A-distance with increased linker length. This is because the longest linkers allow the formation of a porphyrin–C_60_ van-der-Waals dimer (Fig. 12), which is the lowest energy conformer. *Meta* isomers, usually display shorter D–A distances and a higher standard deviation (*i.e.* higher conformational freedom) than their *para*-equivalents.

**Table 4:** Optimum and mean D–A distances.

	Optimum D–A distance [Å]	Mean D–A distance [Å]	*σ* [±Å]
**15a**	9.98	12.27	1.38
**15b**	18.96	18.77	0.24
**16a**	11.16	11.89	1.83
**16b**	19.87	20.57	1.59
**17a**	6.25	8.23	3.53
**17b**	6.13	6.32	0.13

To assess whether these results also apply to the metalated system, we compared the dimerisation energy of unsubstituted H_2_P and Zn porphyrins with C_60_ using dispersion-corrected density functional theory (DFT) (PBE+TS/DND).^78–80^ This reveals that the dimer is stabilized by 2.2 kcal mol^–1^ through metalation. Since the dimer is already the most stable conformation for the free base molecules, the overall picture of the conformational analysis should not change for the metalated case.

Furthermore, the energies of frontier molecular orbitals were calculated for DFT-optimized structures obtained with the MO6 functional.^80,81^ The frontier orbital energies for all computed structures are summarized in Table S3.† In general, the orbital energies are quite constant, with energy differences on the order of several meV. Energy differences of the HOMO orbitals are observed in the presence of the metal atom, which lead to 40 ± 1 meV stabilization. The larger variations observed for the LUMOs are the result of the linkage between the pyridine and the pyrrolidine: *meta*-conformations have LUMO energies around 70 ± 5 meV higher than the corresponding *para*-ones. Analyses of the orbital shapes showed clear electron donor–acceptor interactions. The LUMO is well distributed around the fullerene cage for all the cases (Fig. S9†). The HOMO is mainly localized on the porphyrin and shows a good overlap with the π-orbitals of the benzyl group, which provides electronic coupling with the aromatic chain. The orbitals of the metal atoms showed a high contribution to the electronic distribution of the HOMO around the entire porphyrin, increasing the electronic distribution around it (see Fig. S9 in the ESI†).

## Discussion

Table 2 summarizes the radical ion pair state lifetimes for all electron donor–acceptor conjugates in toluene, THF, and PhCN. In brief, several factors influence the charge transfer of the porphyrin–fullerene D–A conjugates. On one hand, the polarity of the solvent affects the CSS lifetimes. In less polar solvents such as toluene, the longest lifetimes are observed. This leads to the assumption that the charge recombination occurs in the inverted region of the Marcus parabola. On the other hand, the length of the linker plays a key role in the electron transfer dynamics. The systems with just one pyridine-vinylene group exhibit the fastest charge recombination. **16a** (H_2_P-2-*m*C_60_) and **16b** (H_2_P-2-*p*C_60_) feature the longest lived radical ion pairs within the free base porphyrin series, while for the ZnP series **19a** (ZnP-2-*m*C_60_) and **19b** (ZnP-2-*p*C_60_) reveal the longest CSS lifetimes. Astonishingly, the triads with the longest linkers do not show the longest CSS lifetimes. Calculations suggest that the flexible linkers lead to shorter through-space distances between the free base porphyrin and the fullerene. The reduced D–A distances and the shorter lifetimes observed for the radical ion pair for **17a** (H_2_P-3-*m*C_60_), **17b** (H_2_P-3-*p*C_60_), **20a** (ZnP-3-*m*C_60_) and **20b** (ZnP-3-*p*C_60_) lead to the conclusion that for this system electron transfer occurs through space rather than through the linker. The compounds with C_60_ attached to the pyridine in a *para*-positions yield longer-lived CSS than those with C_60_ in a *meta* position. Finally, it should be noted that the longest CS states are formed for **19b** (ZnP-2-*p*C_60_).

## Conclusions

We have designed and synthesized a new series of H_2_P/C_60_ and ZnP/C_60_ electron donor–acceptor conjugates, in which the electron donating H_2_P/ZnP and the electron accepting C_60_ are linked through a pyrrolidine ring covalently attached to pyridine-vinylene spacers of different lengths. Electrochemical experiments and molecular modelling at the DFT level revealed a strong *push–pull* nature between the electroactive constituents. Significant interactions were observed in absorption measurements as 1–4 nm red shifts of the H_2_P/ZnP absorptions. In addition, in the low-energy region of the spectra, charge transfer bands were identified that show considerably stronger interactions for the ZnP conjugates (100–400 cm^–1^) than for the H_2_P conjugates (20–40 cm^–1^). Among the ZnP conjugates, **20b** (ZnP-3-*p*C_60_) exhibits the strongest coupling between ZnP and C_60_. Fluorescence assays showed that the H_2_P/ZnP features depend on the length and the substitution pattern of the pyridine-vinylene spacers. H_2_P systems generally show stronger fluorescence than ZnP ones. Conjugates with just one pyridine-vinylene unit, that is, **15a**, **15b**, **18a**, and **18b**, display the weakest fluorescence and shortest fluorescence lifetimes, while fluorescence quenching is barely detected for the conjugates with three pyridine-vinylene units, **17a**, **17b**, **20a**, and **20b**. Importantly, the fluorescence is more intense in the less polar solvent environments, suggesting charge rather than energy transfer. To confirm this, charge transfer was verified using pump–probe experiments. Differential absorption spectra reveal features of oxidized H_2_P/ZnP and reduced C_60_ in the visible and in the near-infrared regions, respectively. Kinetic analyses yielded charge-separated state lifetimes between 1.3 ns (**15b**) and 65 ns (**16b**) for the H_2_P conjugates and between 373 ps (**18b**) and 165 ns (**19b**) for the ZnP conjugates in THF. In toluene, the H_2_P/ZnP conjugates generally exhibit longer charge-separated state lifetimes than in THF and benzonitrile. This solvent dependence suggests that charge recombination occurs in the Marcus inverted region. Calculations revealed that conjugates with two pyridine-vinylene units and a *para* substitution exhibit the longest distances between D and A, ∼21 Å. These findings are perfectly compatible with the charge-separated state lifetimes for **16b** and **19b**, which were the longest within the series investigated. We infer from a detailed examination of the lowest-energy conformation of the conjugates with the longest spacers that the flexible linkers enable electron donor and acceptor to approach through-space, thus decreasing the effective distance to ∼6–8 Å.

Complementary EPR measurements in frozen PhCN and THF confirm the formation of charge-separated states. A sharp peak corresponding to reduced C_60_ (*g* ∼ 2.000) was identified and a broader, less intense signal (*g* ∼ 2.003) was assigned to oxidized H_2_P/ZnP. Additionally, formation of the charge-separated states was switched on and off repeatedly by turning the irradiation on and off.
