# Supplementary material for: On–off switch of charge-separated states of pyridine-vinylene-linked porphyrin–C60 conjugates detected by EPR
Source: Chem Sci. 2015 Jul 9;6(10):5994–6007. doi: 10.1039/c5sc02051d (PMC5669314; doi:10.1039/c5sc02051d)
Supplement: Supplementary file 1 [file SC-006-C5SC02051D-s001.pdf]

Electronic Supplementary Material (ESI) for Chemical Science.
This journal is © The Royal Society of Chemistry 2015

Electronic Supporting Information

On-Off Switch of Charge-Separated States of Pyridine-Vinylene-Linked Porphyrin-C_{60} Conjugates Detected by EPR

Sabrina V. Kirner,[a,†] Danny Arteaga,[b,†] Christian Henkel,[a] Johannes T. Margraf,[a,c] Nuria Alegret,[d] Kei Ohkubo,[e] Braulio Insuasty,[b] Alejandro Ortiz,*[,b] Nazario Martín,[f] Luis Echegoyen,*[,g] Shunichi Fukuzumi,*[,e] Timothy Clark,[c] and Dirk M. Guldi.*[,a]

[a] Department of Chemistry and Pharmacy and Interdisciplinary Center for Molecular Materials, Friedrich-Alexander-Universität Erlangen-Nürnberg, Egerlandstrasse 3, 91058 Erlangen, Germany

[b] Departamento de Química, Facultad de Ciencias Naturales, Universidad del Valle, A.A. 25360 Cali, Colombia

[c] Department of Chemistry and Pharmacy, Computer Chemistry Center, Friedrich-Alexander-University Erlangen-Nürnberg, Nägelsbachstr. 25, 91052 Erlangen, Germany

[d] Departament de Química Física i Inorgànica, Universitat Rovira i Virgili, 43007, Tarragona (Spain)

[e] Department of Material and Life Science, Graduate School of Engineering, Osaka University, ALCA, Japan Science and Technology Agency (JST), Suita, Osaka 565-0871, Japan

[f] Departamento de Química Orgánica, Facultad de Química, Universidad Complutense 28040, Madrid (Spain)

[g] Department of Chemistry, University of Texas at El Paso, El Paso, Texas 79968-0519, United States

[†]S. V. Kirner and D. Arteaga contributed equally to this work.

Contents

1. Supplementary Figures ii

1.1. Electrochemical experiments.......... ii

1.2. Absorption Spectroscopy iii

1.3. Emission Spectroscopy.......... iv

1.4. Transient Absorption Spectroscopy v

1.5. Molecular Modeling viii

1.6. EPR Spectroscopy ix

2. Experimental Section xii

3. Synthetic Details.......... xii

3.1. Procedure for the preparation of the pyridine-vinylene linkers **2-6a** and **2-6b**.......... xii

3.2. General procedure for the preparation of the electroactive dyads derivatives **9-10a,b** (H_2P-n-*m*CHO, H_2P-n-*p*CHO) y **12-13a,b** (ZnP-n-*m*CHO, ZnP-n-*p*CHO).......... xiv

3.3. General procedure for the preparation of the electroactive dyads derivatives **11a,b** (H_2P-3-*m*CHO, H_2P-3-*p*CHO) and **14a,b** (ZnP-3-*m*CHO, ZnP-3-*p*CHO).......... xiv

3.4 General procedure for the preparation and spectroscopic data for the new porphyrin-fullerene conjugates **15-20a** (H_2P-n-*m*C_{60}, ZnP-n-*m*C_{60}, n=1-3) and **15-20b** (H_2P-n-*p*C_{60}, ZnP-n-*p*C_{60}, n=1-3). xv

4. Characterization.......... xvii

4.1 ^{1}H-NMR spectra and spectroscopic data for the compounds **9-14a,b** xvii

4.2. ^{1}H-NMR and ^{13}C-NMR spectra for new porphyrin-fullerene conjugates **15-20a** (H_2P-n-*m*C_{60}, ZnP-n-*m*C_{60}, n=1-3) and **15-20b** (H_2P-n-*p*C_{60}, ZnP-n-pC_{60}, n=1-3). xxiii

4.3.- MALDI-TOF spectra for the new D-π-A electroactive conjugates **15-20a** and **15-20b** xxxii

1. Supplementary Figures

1.1. Electrochemical experiments

Table S1: Electrochemical data of the redox processes of porphyrin-fullerene conjugates **15-20a** (H_2P-n-*m*C_{60} and ZnP-n-*m*C_{60}, n=1-3) and **15-20b** (H_2P-n-*p*C_{60} and ZnP-n-*p*C_{60}, n=1-3) and C_{60} used a reference compound detected by CV. Potentials in V (half-wave potentials: $E_{1/2}$); Experimental conditions: scan rate 100 mV/s; glassy carbon working electrode; Ag-wire reference electrode; Pt-wire as a counter electrode; 0.1 M $TBAPF_6$ supporting electrolyte in free-water DCM; room temperature.

Comp.	$E^1_{1/2,red}$	$E^2_{1/2,red}$	$E^3_{1/2,red}$	$E^4_{1/2,red}$	$E^5_{1/2,red}$	$E^1_{1/2,ox}$	$E^2_{1/2,ox}$
C_{60}	-0.977	-1.369	-	-1.817	-2.253	-	-
**15a,** H_2P-1-*m*C_{60}	-1.122	-1.500	-1.698	-2.021	-	0.457	-
**15b,** H_2P-1-*p*C_{60}	-1.102	-1.477	-1.693	-2.001	-	0.447	-
**16a,** H_2P-2-*m*C_{60}	-1.119	-1.488	-1.687	-2.004	-	0.455	-
**16b,** H_2P-2-*p*C_{60}	-1.095	-1.469	-1.719	-2.011	-	0.443	-
**17a,** H_2P-3-*m*C_{60}	-1.125	-1.485	-1.674	-2.013	-	0.437	-
**17b,** H_2P-3-*p*C_{60}	-1.089	-1.474	-1.692	-2.005	-	0.445	-
**18a,** ZnP-1-*m*C_{60}	-1.104	-1.475	-1.821	-1.993	-2.209	0.306	0.628
**18b,** ZnP-1-*p*C_{60}	-1.086	-1.466	-1.831	-1.984	-2.224	0.277	0.645
**19a,** ZnP-2-*m*C_{60}	-1.109	-1.464	-1.856	-2.001	-2.253	0.292	0.639
**19b,** ZnP-2-*p*C_{60}	-1.090	-1.462	-1.877	-1.994	-2.259	0.283	0.677
**20a,** ZnP-3-*m*C_{60}	-1.109	-1.469	-1.823	-2.004	-2.152	0.271	0.627
**20b,** ZnP-3-*p*C_{60}	-1.086	-1.458	-1.845	-1.994	-2.187	0.271	0.692

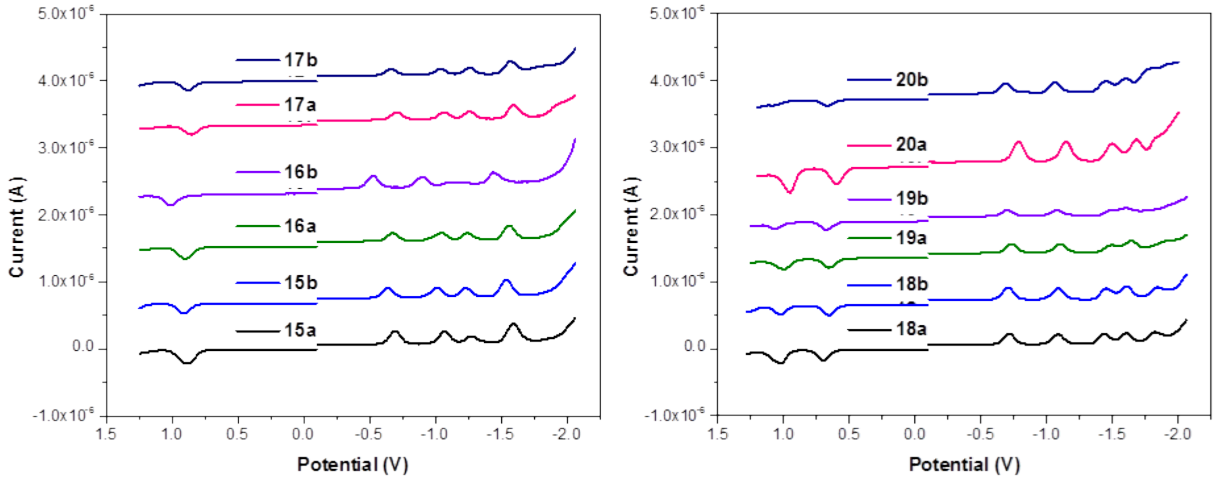

Figure S1. Differential pulse voltammograms of porphyrin-fullerene conjugates; Left: **15-17a,b** (H_2P-n-*m*C_{60} and H_2P-n-*p*C_{60}, n=1-3); Right: **18-20a,b** (ZnP-n-*m*C_{60} and ZnP-n-*p*C_{60}, n=1-3). Reductive scans between 0 and -2.05 V; oxidative scans between 0 and 1.25 V; Exp. conditions: scan rate 100 mV/s; glassy carbon, Ag-wire and Pt-wire as working, reference and counter electrodes; 0.1 M $TBAPF_6$ supporting electrolyte in free-water CH_2Cl_2.

1.2. Absorption Spectroscopy

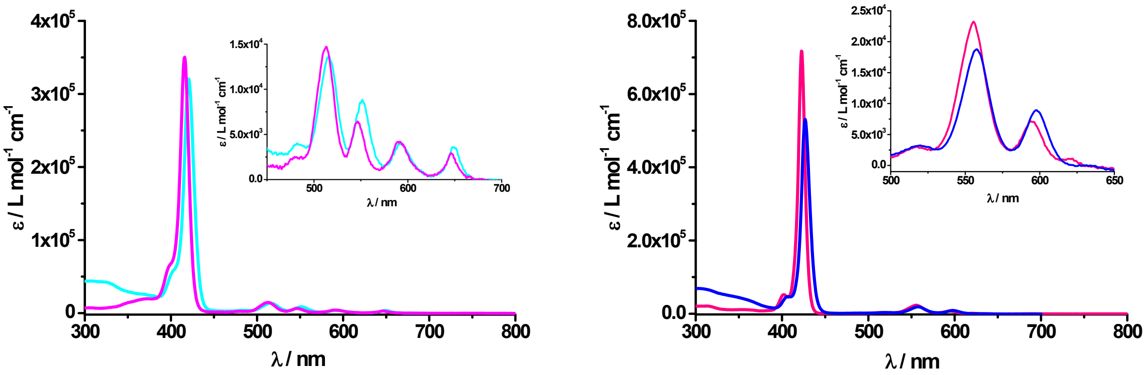

Figure S2. Left: UV-Vis absorption spectra of compounds H_2P-1-*m*C_{60} (cyan) and H_2TPP (magenta) in THF at room temperature; Inlet: Zoom into the Q bands; Right: UV-Vis absorption spectra of compounds ZnP-2-*m*C_{60} (blue) and ZnTPP (magenta) in THF at room temperature; Inlet: Zoom into the Q bands.

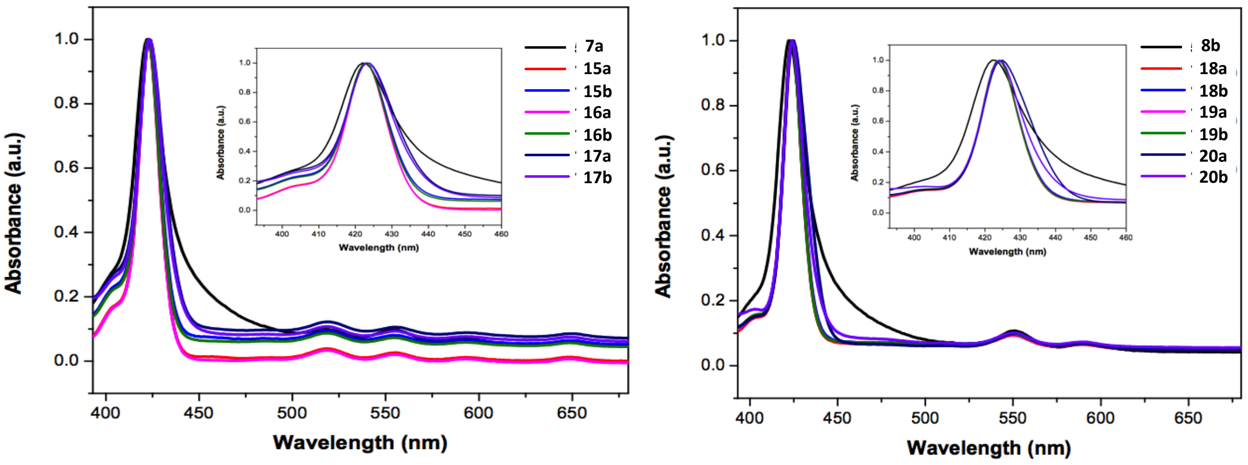

Figure S3. Left: Normalized UV-Vis absorption spectra of compounds **15-17a,b** (H_2P-n-*m*C_{60}, H_2P-n-*p*C_{60}) in CH_2Cl_2 diluted solutions at room temperature; Inlet: Zoom into the Soret bands; Right: Normalized UV-Vis absorption spectra of compounds **18-20a,b** (ZnP-n-*m*C_{60}, ZnP-n-*p*C_{60}) in CH_2Cl_2 diluted solutions at room temperature; Inlet: Zoom into the Soret bands.

1.3. Emission Spectroscopy

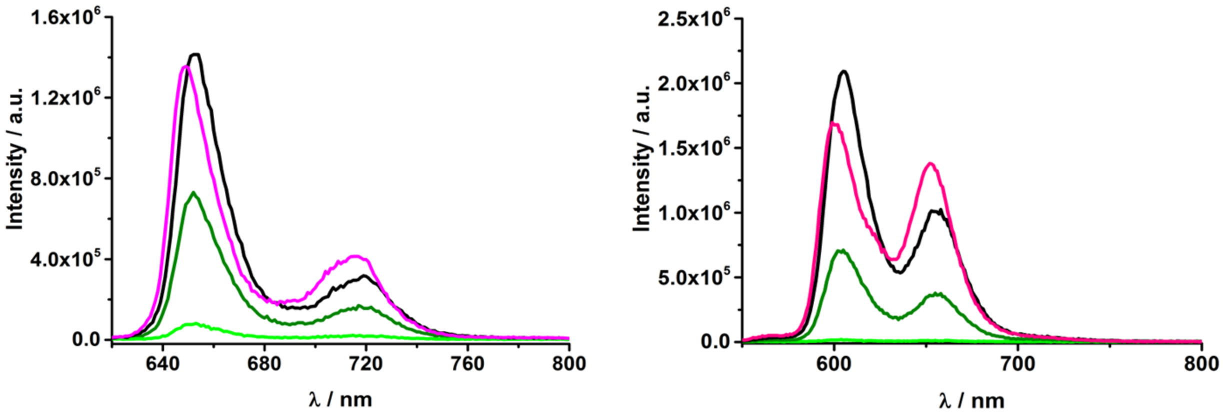

Figure S4. Left: emission spectra of compounds **(15b)** H_2P-1-*p*C_{60} (green); **(16b)** H_2P-2-*p*C_{60} (olive); **(17b)** H_2P-3-*p*C_{60} (black) and H_2TPP (magenta) (excitation at 420 nm; OD = 0.02) in THF at room temperature. Right: emission spectra of compounds **(18b)** ZnP-1-*p*C_{60} (green); **(19b)** ZnP-2-*p*C_{60} (olive); **(20b)** ZnP-3-*p*C_{60} (black) and ZnTPP (pink) (excitation at 420 nm; OD = 0.06) in THF at room temperature.

1.4. Transient Absorption Spectroscopy

Table S2. CS Lifetimes of Porphyrin-Fullerene Conjugates in Toluene and PhCN at 298 K.

	compound	Toluene	PhCN
5a	H_2P-1-*m*C_{60}	2.0 ns	630 ps
15b	H_2P-1-*p*C_{60}	2.8 ns	740 ps
16a	H_2P-2-*m*C_{60}	65 ns	4.1 ns
16b	H_2P-2-*p*C_{60}	115 ns	5.3 ns
17a	H_2P-3-*m*C_{60}	4.2 ns	850 ps
18a	ZnP-1-*m*C_{60}	1.5 ns	140 ps
18b	ZnP-1-*p*C_{60}	1.6 ns	130 ps
19a	ZnP-2-*m*C_{60}	145 ns	2.5 ns
19b	ZnP-2-*p*C_{60}	395 ns	2.5 ns
10a	ZnP-1-*m*C_{60}	1.5 ns	1.5 ns
20b	ZnP-3-*p*C_{60}	2.0 ns	1.4 ns

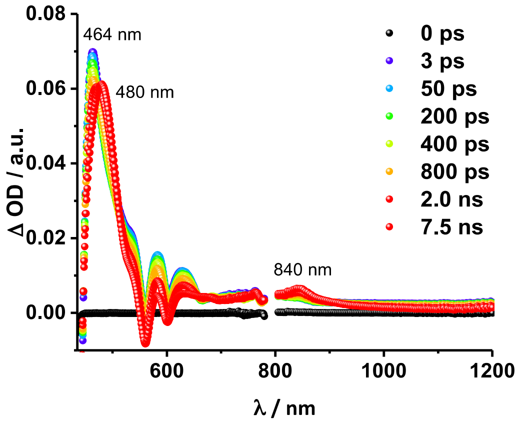

Figure S5. Differential absorption spectra (visible and near-infrared) registered upon femtosecond flash photolysis (420 nm, 150 nJ) with time delays of 0 ps to 7.5 ns at room temperature of compound **(12b)** ZnP-1-*p*-CHO in THF at room temperature.

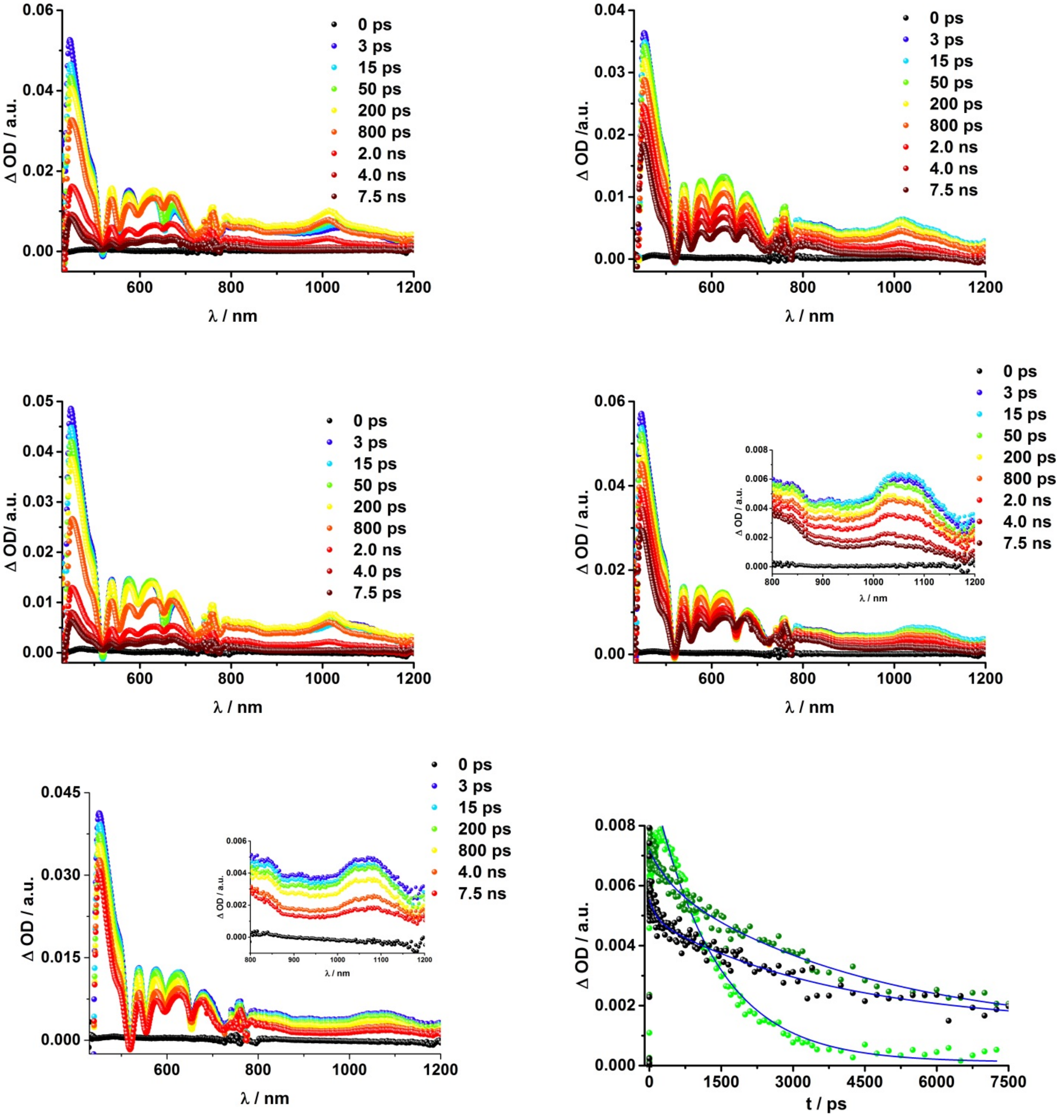

Figure S6. Differential absorption spectra (visible and near-infrared) registered upon femtosecond flash photolysis (387 nm, 200 nJ) with time delays of 0 ps to 7.5 ns at room temperature of compounds **(15a)** H_2P-1-*m*C_{60} (top left); **(17a)** H_2P-3-*m*C_{60} (top right); **(15b)** H_2P-1-*p*C_{60} (center left); **(16b)** H_2P-2-*p*C_{60} (center right) and **(17b)** H_2P-3-*p*C_{60} (bottom left). Bottom right: Time absorption profiles of **(15b)** H_2P-1-*p*C_{60} (green); **(16b)** H_2P-2-*p*C_{60} (olive) and **(17b)** H_2P-3-*p*C_{60} (black) at 1010 nm, monitoring the charge recombination process.

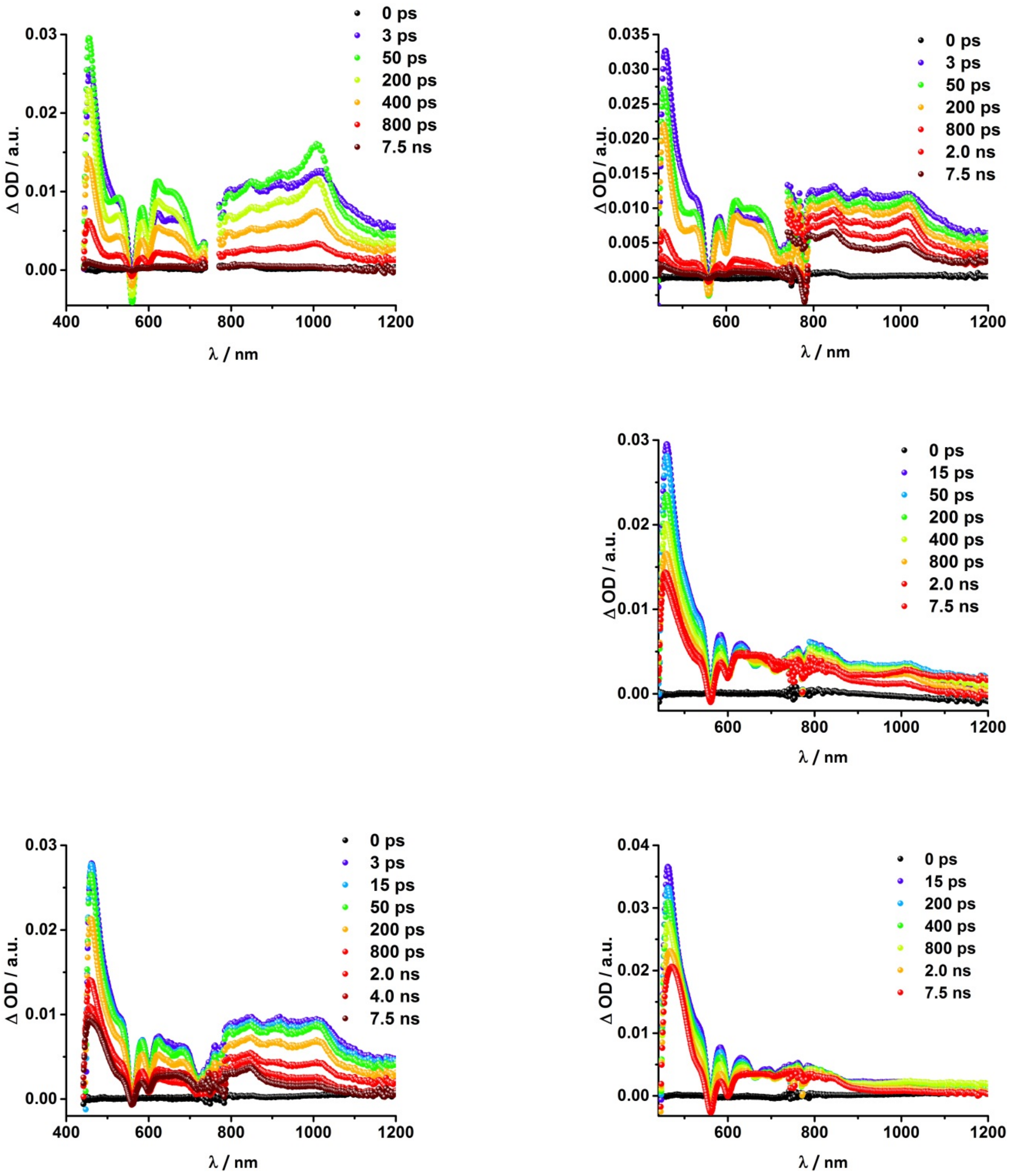

Figure S7: Differential absorption spectra (visible and near-infrared) registered upon femtosecond flash photolysis (387 nm, 200 nJ) with time delays of 0 ps to 7.5 ns at room temperature of compounds **(18a)** ZnP-1-*m*C_{60} (top left); **(18b)** ZnP-1-*p*C_{60} (top right); **(19b)** ZnP-2-*p*C_{60} (center right); **(20a)** ZnP-3-*m*C_{60} (bottom left) and **(20b)** ZnP-3-*p*C_{60} (bottom right).

1.5. Molecular Modeling

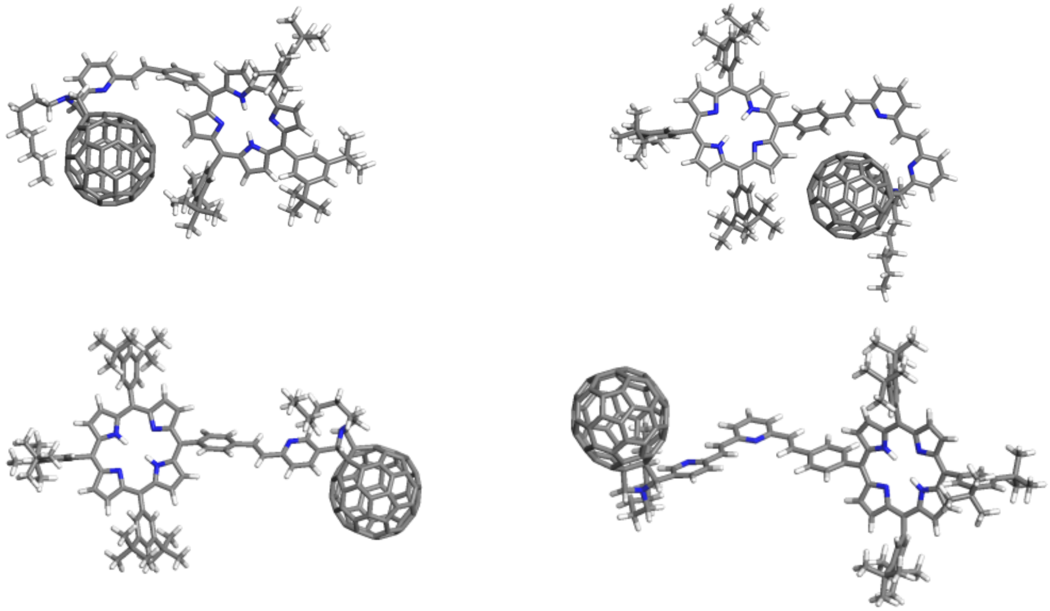

Figure S8. Lowest energy conformations of **H_2P-1-*m*C_{60}** (top left), **H_2P-2-*m*C_{60}** (top right), **H_2P-1-*p*C_{60}** (bottom left), **H_2P-2-*p*C_{60}** (bottom right).

Table S3. Computed energy levels of the frontier orbitals HOMO-LUMO of all calculated structures **15-20a** and **15-20b.** All energies are reported in eV.

Compound	HOMO	LUMO	Compound	HOMO	LUMO
**15a,** H_2P-1-*m*C_{60}	-4.34	-2.48	**18a,** ZnP-1-*m*C_{60}	-4.38	-2.48
**15b,** H_2P-1-*p*C_{60}	-4.34	-2.55	**18b,** ZnP-1-*p*C_{60}	-4.38	-2.55
**16a,** H_2P-2-*m*C_{60}	-4.34	-2.49	**19a,** ZnP-2-*m*C_{60}	-4.38	-2.48
**16b,** H_2P-2-*p*C_{60}	-4.34	-2.55	**19b,** ZnP-2-*p*C_{60}	-4.38	-2.55
**17a,** H_2P-3-*m*C_{60}	-4.33	-2.49	**20a,** ZnP-3-*m*C_{60}	-4.38	-2.49
**17b,** H_2P-3-*p*C_{60}	-4.35	-2.56	**20b,** ZnP-3-pC_{60}	-4.40	-2.56

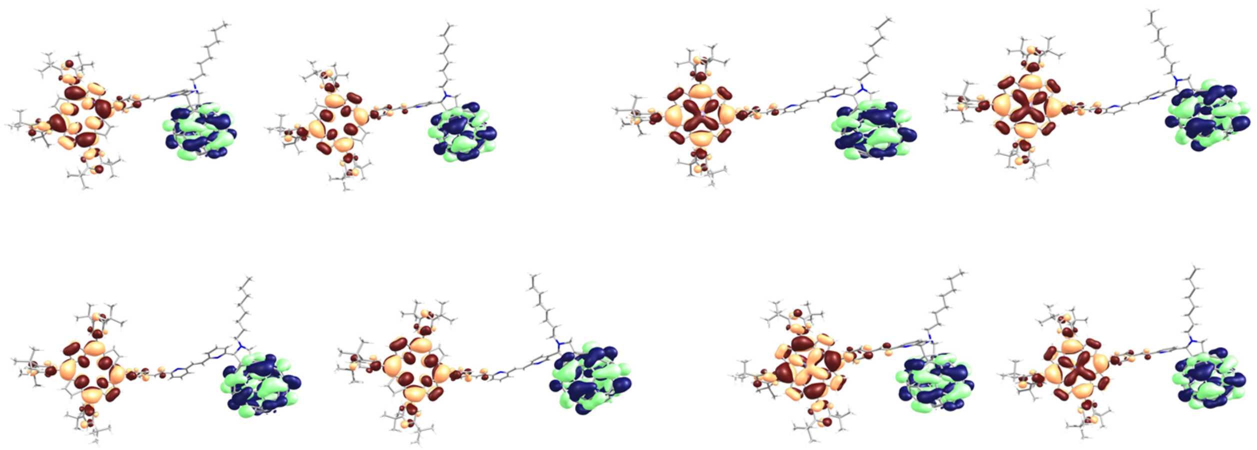

Figure S9. Frontier orbitals HOMO (red-orange) and LUMO (blue-green) of **(15a)** H_2P-1-*m*C_{60} and **(15b)** H_2P-1-*p*C_{60} (top left); **(19a)** ZnP-2-*m*C_{60} and **(19b)** ZnP-2-*p*C_{60} (top right); **(16a)** H_2P-2-*m*C_{60} and **(16b)** H_2P-2-*p*C_{60} (bottom left); **(18a)** ZnP-1-*m*C_{60} and **(18b)** ZnP-1-*p*C_{60} (bottom right).

1.6. EPR Spectroscopy

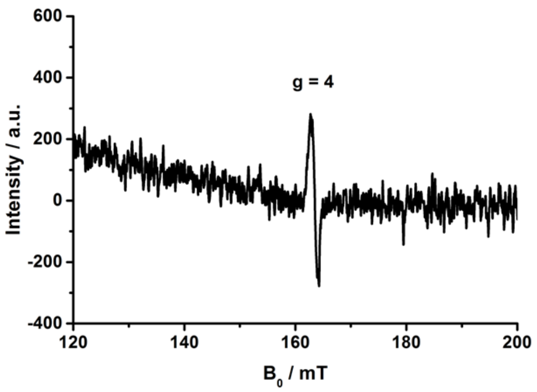

Figure S10. EPR signals observed under photoirradiation of **(17a)** H_2P-3-*m*C_{60} in PhCN at 77 K.

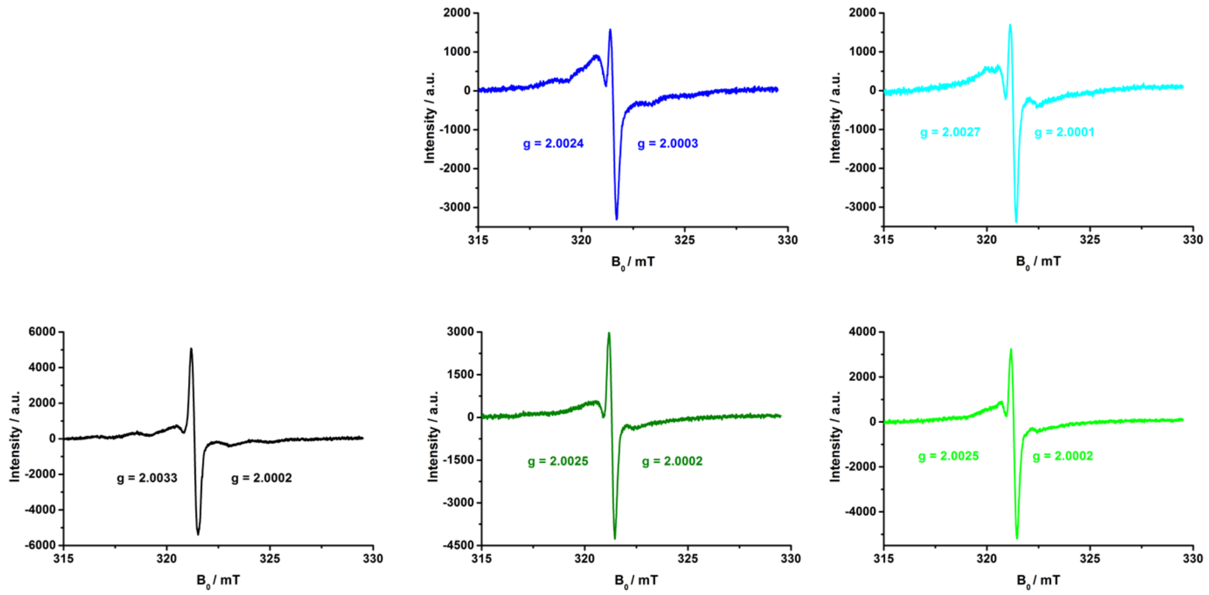

Figure S11. EPR signals observed under photoirradiation including g values of **(16a)** H_2P-2-*m*C_{60} (top center), **(17a)** H_2P-3-*m*C_{60} (top right), **(15b)** H_2P-1-*p*C_{60} (bottom left), **(16b)** H_2P-2-*p*C_{60} (bottom center) and **(17b)** H_2P-3-*p*C_{60} (bottom right) in PhCN at 77 K.

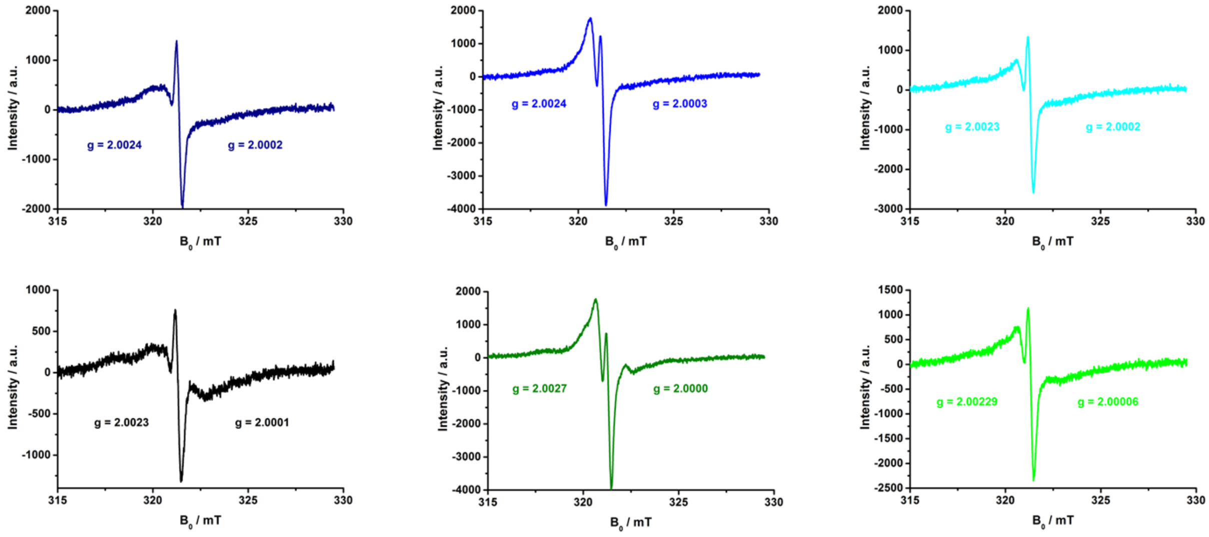

Figure S12. EPR signals observed under photoirradiation **(18a)** ZnP-1-*m*C_{60} (top left), **(19a)** ZnP-2-*m*C_{60} (top center), **(20a)** ZnP-3-*m*C_{60} (top right), **(18b)** ZnP-1-*p*C_{60} (bottom left), **(19b)** ZnP-2-*p*C_{60} (bottom center), **(20b)** ZnP-3-*p*C_{60} (bottom right) in PhCN at 77 K.

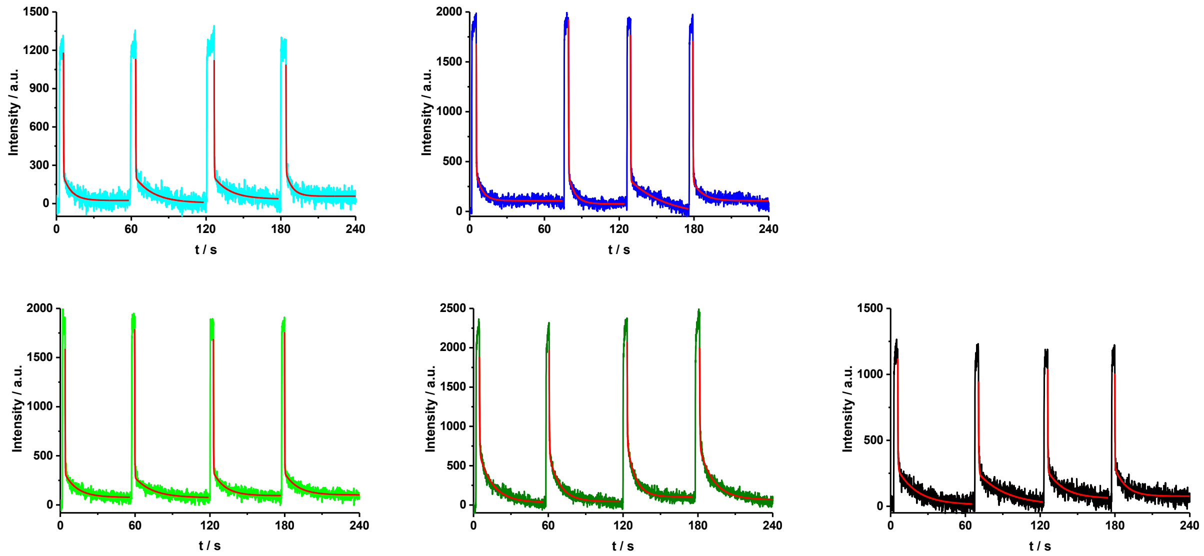

Figure S13. On-off switch of EPR signal due the CS state of **(15a)** H_2P-1-*m*C_{60} (top left), **(16a)** H_2P-2-*m*C_{60} (top center), **(15b)** H_2P-1-*p*C_{60} (bottom left), **(16b)** H_2P-2-*p*C_{60} (bottom center) and **(17b)** H_2P-3-*p*C_{60} (bottom right) in PhCN at 77 K by turning on and off the irradiation light with a mercury lamp.

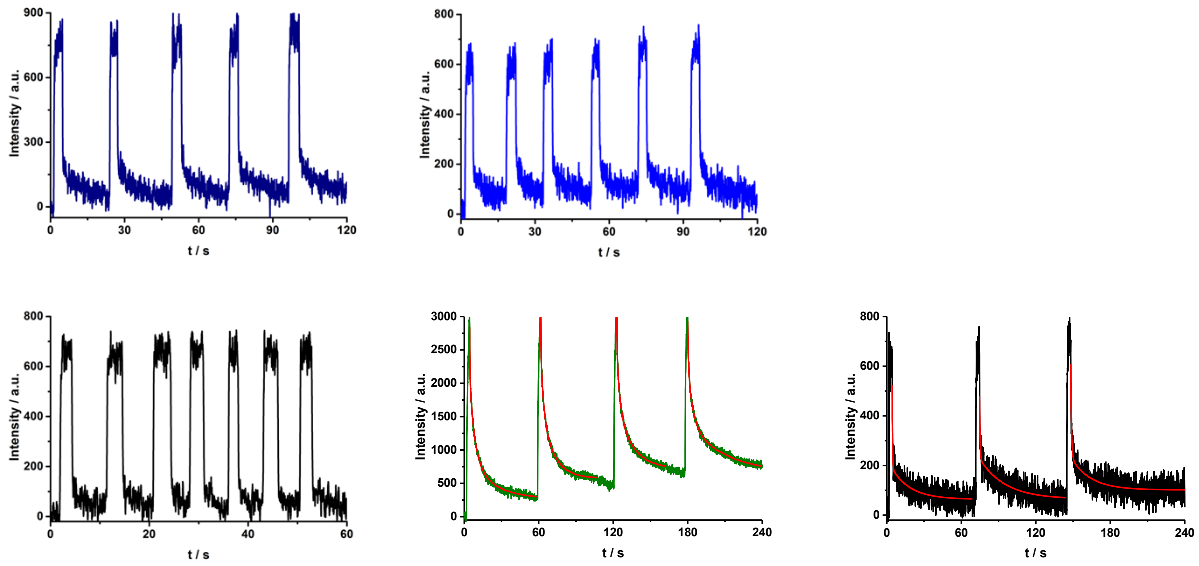

Figure S14. On-off switch of EPR signal due the CS state **(18a)** ZnP-1-*m*C_{60} (top left), **(19a)** ZnP-2-*m*C_{60} (top center), **(18b)** ZnP-1-*p*C_{60} (bottom left), **(19b)** ZnP-2-*p*C_{60} (bottom center), **(20b)** ZnP-3-*p*C_{60} (bottom right) in PhCN at 77 K by turning on and off the irradiation light with a mercury lamp.

2. Experimental Section

Materials and reagents: For all reactions and chemical procedures the reagents and solvents were purchased from different commercial sources and were used without any further purification. Both toluene and tetrahydrofurane (THF) were dried over metallic sodium using benzophenone as indicator system and freshly distilled before use. Tetrabutylammonium hexafluorophosphate ($TBAPF_6$) used as supporting electrolyte in the electrochemical measurements was recrystallized from absolute ethanol and dried for one day under vacuum before use.

Characterization: The ^{1}H-NMR and ^{13}C-NMR spectra were recorded on JEOL (600 MHz) and Brücker (400 MHz) UltraShield avance II NMR spectrometers at room temperature with tetramethylsilane as the internal standard. Matrix assisted laser desorption ionization (coupled to a time-of-flight analyzer) MALDI-TOF experiments were performed with a Bruker MicroFlex LRF mass spectrometer using 2,5-dihydroxybenzoic acid (DHB) as the matrix. The UV-Vis absorption spectra of the new compounds in diluted dichloromethane solutions were measured with a Varian Cary 5000 UV-Vis-NIR and Shimadzu PharmaSpec UV-1700 spectrophotometers. Emission spectra of the electroactive compounds in diluted dichloromethane and tetrahydrofurane solutions were measured with a Jasco FP-8500 spectrometer. The electrochemical measurements were determined at room temperature at scan rate of 100 mV/s with an autolab PGStat 30 electrochemical workstation by using a conventional three-electrode cell with a glassy carbon working electrode (1.0 mm), a platinum wire counter electrode (Aldrich, 1.0 mm), and a silver wire pseudoreference electrode (Aldrich, 1.0 mm); 0.1 M tetrabutylammonium hexafluorophosphate ($TBAPF_6$) was used as the supporting electrolyte in anhydrous dichloromethane (DCM, Sigma-Aldrich, anhydrous, 99%). For each measurement all the samples were degassed with a gentle flow of argon for 5 min prior to the scans. The ferrocene/ferrocenium (Fc/Fc^+) redox couple was used as an internal potential reference. The calculations of the molecular geometry and molecular orbitals HOMO-LUMO of all systems were carried out using the Gaussian-09 package at the density functional theory (DFT) level. M06 was the meta-hybrid functional employed, which includes the last dispersion approximation performed by Truhlar. The basis set chosen for all the atoms was the Pople 6-31G**.

3. Synthetic Details

3.1. Procedure for the preparation of the pyridine-vinylene linkers **2-6a** and **2-6b**.

The vinyl-pyridines oligomers were obtained by successive cross-coupling reactions catalyzed by palladium as the Stille and Heck type from 6-bromo-2-pyridinecarboxaldehyde, 6-bromo-3-pyridine-carboxaldehyde and 2,6-dibromopyridine as the main building blocks.

Compound 2a: A mixture of 6-bromo-2-pyridine-carboxaldehyde **1a** (1.0 g, 5.41 mmol), $Pd(PPh_3)_4$ (312 mg, 0.27 mmol) was dissolved in 30 mL of anhydrous toluene and argon was bubbled for 30 minutes, then was added drop to drop tributyl(vinyl)tin (2.05 mL, 7.03 mmol), the reaction mixture under inert atmosphere, was refluxed for 20 hours. The reaction mixture was allowed to reach room temperature and the solvent was removed to dryness and extracted with CH_2Cl_2 (3x40 mL), organic phase was dried over anhydrous magnesium sulfate, filtered and the solvent removed under reduced pressure. The residue was purified by column chromatography on silica gel in CH_2Cl_2/ CH_3OH (30/1) mixture to give the desired product **2a** as a yellow oil (82%). ^{1}H-NMR (400 MHz, $CDCl_3$) δ_H = 5.60 (dd, J_1 = 10.8 Hz, J_2 = 1.3 Hz, 1H), 6.33 (dd, J_1 = 17.4 Hz, J_2 = 1.1 Hz, 1H), 6.88 (dd, J_1 = 17.6 Hz, J_2 = 10.8 Hz, 1H), 7.53-7.55 (m, 1H), 7.75-7.81 (m, 2H), 10.05 (s, 1H) ppm; ^{13}C-NMR (100 MHz, $CDCl_3$) δ_C = 119.5, 124.8, 135.5, 137.1, 152.2, 155.9, 193.4 ppm; FTIR (KBr) υ = 2971, 2945, 2852, 1715, 1593, 1578, 1487, 1345 cm^{-1}; MS (EI): *m/z* 133 $[M]^+$.

Compound 2b: A mixture of 6-bromo-3-pyridine-carboxaldehyde **1b** (1.0 g, 5.41 mmol), $Pd(PPh_3)_4$ (312 mg, 0.27 mmol) was dissolved in 30 mL of anhydrous toluene and argon was bubbled for 30 minutes, then was added drop to drop tributyl(vinyl)tin (2.05 mL, 7.03 mmol), the reaction mixture under inert atmosphere, was refluxed for 22 hours. The reaction mixture was allowed to reach room temperature and the solvent was removed to dryness and extracted with CH_2Cl_2 (3x40 mL), organic phase was dried over anhydrous magnesium sulfate, filtered and the solvent removed under reduced pressure. The residue was purified by column chromatography on silica gel in $CHCl_3$/ CH_3OH (30/1) mixture to give the desired product **2b** as a yellow oil (86%). ^{1}H-NMR (400 MHz, $CDCl_3$) δ_H = 5.69 (d, J = 10.8 Hz, 1H), 6.41 (d, J = 17.3 Hz, 1H), 6.89 (dd, J_1 = 17.6 Hz, J_2 = 10.8 Hz, 1H), 7.49 (d, J = 8.0 Hz, 1H), 8.13 (dd, J_1 = 8.2 Hz, J_2 = 2.1 Hz, 1H), 9.02 (s, 1H); 10.09 (s, 1H) ppm; ^{13}C-RMN (100 MHz, $CDCl_3$) δ_C = 121.5, 128.1, 131.6, 132.5, 155.2, 158.7, 199.3 ppm; FTIR (KBr) υ = 2858, 2829, 2774, 1706, 1589, 1570, 1486, 1457, 1375 cm^{-1}; MS (EI): *m/z* 133 $[M]^+$.

Compound 4a: A mixture of 6-vinyl-2-pyridine-carboxaldehyde **2a** (250 mg, 1.88 mmol), 2,6-dibromopyridine **3** (441 mg, 1.88 mmol), $Pd(OAc)_2$ (40 mg, 0.18 mmol), tetrabutylammonium bromide Bu_4NBr (1.8 g, 5.64 mmol) and K_2CO_3 (778 mg, 5.64 mmol)

was dissolved in 30 mL of anhydrous DMF and argon was bubbled for 30 minutes, the reaction mixture under inert atmosphere, was refluxed for 24 hours. The reaction mixture was allowed to reach room temperature and the solvent was removed to dryness and extracted with CH_2Cl_2 (3x30 mL), the solvent was removed under reduced pressure and the residue was purified by column chromatography on silica gel in $CHCl_3$/ CH_3OH (40/1) mixture to give the desired product **4a** as a white solid (65%). ^{1}H-NMR (600 MHz, $CDCl_3$) δ_H = 7.40 (dd, J_1 = 7.9 Hz, J_2 = 2.4 Hz, 2H), 7.55 (t, J = 7.6 Hz, 1H), 7.63 (dd, J_1 = 6.9 Hz, J_2 = 2.1 Hz, 1H), 7.74-7.80 (m, 2H), 7.84-7.88 (m, 2H), 10.10 (s, 1H) ppm; ^{13}C-NMR (150 MHz, $CDCl_3$) δ_C = 120.6, 122.2, 127.3, 127.4, 131.6, 132.1, 137.9, 139.1, 142.5, 152.9, 155.2, 156.0, 193.8 ppm; FTIR (KBr) υ = 2920, 2868, 1718, 1578, 1544, 1451, 1428, 1324 cm^{-1}; MS (EI): *m/z* 289 $[M+2]^+$, 287 $[M]^+$.

Compound 4b: A mixture of 6-vinyl-3-pyridine-carboxaldehyde **2b** (250 mg, 1.88 mmol), 2,6-dibromopyridine **3** (441 mg, 1.88 mmol), $Pd(OAc)_2$ (40 mg, 0.18 mmol), tetrabutylammonium bromide Bu_4NBr (1.8 g, 5.64 mmol) and K_2CO_3 (778 mg, 5.64 mmol) was dissolved in 50 mL of anhydrous DMF and argon was bubbled for 30 minutes, the reaction mixture under inert atmosphere, was refluxed for 24 hours. The reaction mixture was allowed to reach room temperature and the solvent was removed to dryness and extracted with CH_2Cl_2 (3x30 mL), the solvent was removed under reduced pressure and the residue was purified by column chromatography on silica gel in CH_2Cl_2/ CH_3OH (20/1) mixture to give the desired product **4b** as a white solid (68%). ^{1}H-NMR (600 MHz, $CDCl_3$) δ_H = 7.37 (d, J = 7.6 Hz, 1H), 7.40 (d, J = 8.2 Hz, 1H), 7.55 (t, J = 8.3 Hz, 2H), 7.76-7.81 (m, 2H), 8.15-8.16 (m, 1H), 9.06 (s, 1H), 10.09 (s, 1H) ppm; ^{13}C-NMR (150 MHz, $CDCl_3$) δ_C = 122.9, 123.9, 127.7, 130.4, 132.1, 133.5, 136.9, 139.1, 142.6, 152.3, 155.6, 159.5, 190.2 ppm; FTIR (KBr) υ = 2931, 2871, 1690, 1588, 1550, 1481, 1432, 1366 cm^{-1}; MS (EI): *m/z* 289 $[M+2]^+$, 287 $[M]^+$.

Compound 5a: A mixture of dimer **4a** (144 mg, 0.50 mmol), $Pd(PPh_3)_4$ (29 mg, 0.03 mmol) was dissolved in 20 mL of anhydrous toluene and argon was bubbled for 30 minutes, then was added drop to drop tributyl(vinyl)tin (0.20 mL, 0.65 mmol), the reaction mixture under inert atmosphere, was refluxed for 20 hours. The reaction mixture was allowed to reach room temperature and the solvent was removed to dryness and extracted with CH_2Cl_2 (3x40 mL), organic phase was dried over anhydrous magnesium sulfate, filtered and the solvent removed under reduced pressure. The residue was purified by column chromatography on silica gel in $CHCl_3$/ CH_3OH (30/1) mixture to give the desired product **5a** as a yellow solid (75%). ^{1}H-NMR (400 MHz, $CDCl_3$) δ_H = 5.45 (d, J = 10.8 Hz, 1H), 6.25 (d, J = 17.3 Hz, 1H), 6.79 (dd, J_1 = 17.3 Hz, J_2 = 10.8 Hz, 1H), 7.19 (dd, J_1 = 8.0 Hz, J_2 = 2.4 Hz, 2H), 7.38 (t, J = 7.6 Hz, 1H), 7.48 (dd, J_1 = 6.9 Hz, J_2 = 2.1 Hz, 1H), 7.56-7.64 (m, 2H), 7.72-7.83 (m, 2H), 10.05 (s, 1H) ppm; ^{13}C-NMR (150 MHz, $CDCl_3$) δ_C = 118.7, 120.2, 120.6, 122.1, 126.7, 128.6, 130.7, 132.0, 133.6, 136.9, 137.6, 155.7, 155.9, 193.8 ppm; FTIR (KBr) υ = 2956, 2924, 2817, 1713, 1583, 1566, 1456, 1324 cm^{-1}; MS (EI): *m/z* 236 $[M]^+$.

Compound 5b: A mixture of dimer **4b** (144 mg, 0.50 mmol), $Pd(PPh_3)_4$ (29 mg, 0.03 mmol) was dissolved in 30 mL of anhydrous toluene and argon was bubbled for 30 minutes, then was added drop to drop tributyl(vinyl)tin (0.20 mL, 0.65 mmol), the reaction mixture under inert atmosphere, was refluxed for 20 hours. The reaction mixture was allowed to reach room temperature and the solvent was removed to dryness and extracted with CH_2Cl_2 (3x40 mL), organic phase was dried over anhydrous magnesium sulfate, filtered and the solvent removed under reduced pressure. The residue was purified by column chromatography on silica gel in CH_2Cl_2/ CH_3OH (20/1) mixture to give the desired product **5b** as a yellow solid (80%). ^{1}H-NMR (600 MHz, $CDCl_3$) δ_H = 5.50 (d, J = 11.0 Hz, 1H), 6.29 (d, J = 17.9 Hz, 1H), 6.82 (dd, J_1 = 17.5 Hz, J_2 = 10.7 Hz, 1H), 7.21 (d, J = 8.2 Hz, 1H), 7.29 (d, J = 8.2 Hz, 1H), 7.55 (d, J = 8.2 Hz, 1H), 7.63 (t, J = 7.6 Hz, 1H), 7.77-7.83 (m, 2H), 8.12 (dd, J_1 = 7.6 Hz, J_2 = 2.1 Hz, 1H), 9.02 (s, 1H), 10.05 (s, 1H) ppm; ^{13}C-NMR (150 MHz, $CDCl_3$) δ_C = 118.9, 121.1, 122.9, 123.3, 130.1, 130.8, 135.6, 136.6, 137.0, 137.2, 152.4, 153.8, 155.9, 160.3, 190.3 ppm; FTIR (KBr) υ = 2842, 2799, 2754, 1700, 1586, 1562, 1468, 1449, 1364, 1327 cm^{-1}; MS (EI): *m/z* 236 $[M]^+$.

Compound 6a: A mixture of 6-vinyl-2-pyridine-carboxaldehyde **2a** (250 mg, 1.88 mmol), 2,6-dibromopyridine **3** (148 mg, 0.63 mmol), $Pd(OAc)_2$ (40 mg, 0.18 mmol), tetrabutylammonium bromide Bu_4NBr (1.8 g, 5.64 mmol) and K_2CO_3 (778 mg, 5.64 mmol) was dissolved in 30 mL of anhydrous DMF and argon was bubbled for 30 minutes, the reaction mixture under inert atmosphere, was refluxed for 24 hours. The reaction mixture was allowed to reach room temperature and the solvent was removed to dryness and extracted with CH_2Cl_2 (3x30 mL), the solvent was removed under reduced pressure and the residue was purified by column chromatography on silica gel in CH_2Cl_2/CH_3OH (30/1) mixture to give the desired product **6a** as a yellow solid (62%). ^{1}H-NMR (600 MHz, $CDCl_3$) δ_H = 7.41 (d, J = 7.6 Hz, 2H), 7.42 (d, J = 7.6 Hz, 1H), 7.71-7.73 (m, 4H), 7.75 (d, J = 18.3 Hz, 1H), 7.84-7.88 (m, 2H), 7.89-7.91 (m, 2H), 7.93 (d, J = 6.9 Hz, 1H), 10.14 (s, 2H) ppm; ^{13}C-NMR (150 MHz, $CDCl_3$) δ_C = 120.4, 120.8, 122.8, 123.7, 126.8, 130.8, 131.2, 133.5, 153.9, 154.6, 155.9, 193.9 ppm; FTIR (KBr) υ = 2925, 2846, 1708, 1583, 1563, 1455, 1277 cm^{-1}; MS (EI): *m/z* 341 $[M]^+$.

Compound 6b: A mixture of 6-vinyl-3-pyridine-carboxaldehyde **2b** (250 mg, 1.88 mmol), 2,6-dibromopyridine **3** (148 mg, 0.63 mmol), $Pd(OAc)_2$ (40 mg, 0.18 mmol), tetrabutylammonium bromide Bu_4NBr (1.8 g, 5.64 mmol) and K_2CO_3 (778 mg, 5.64 mmol) was dissolved in 30 mL of anhydrous DMF and argon was bubbled for 30 minutes, the reaction mixture under inert atmosphere, was refluxed for 24 hours. The reaction mixture was allowed to reach room temperature and the solvent was removed to dryness and extracted with CH_2Cl_2 (3x30 mL), the solvent was removed under reduced pressure and the residue was purified by column

chromatography on silica gel in CH_2Cl_2/CH_3OH (20/1) mixture to give the desired product **6b** as a yellow solid (70%). ^{1}H-NMR (600 MHz, $CDCl_3$) δ_H = 7.40 (d, *J* = 8.2 Hz, 1H), 7.63-7.66 (m, 2H), 7.75 (t, *J* = 7.6 Hz, 1H), 7.86-7.95 (m, 5H), 8.18-8.19 (m, 2H), 9.08 (s, 2H), 10.11 (s, 2H) ppm; ^{13}C-NMR (150 MHz, $CDCl_3$) δ_C = 122.2, 124.7, 132.6, 136.7, 140.9, 143.1, 145.6, 148.4, 152.4, 160.3, 161.0, 189.3 ppm; FT-IR (KBr) υ = 2924, 2848, 1693, 1589, 1562, 1479, 1446, 1364 cm^{-1}; MS (EI): *m/z* 341 $[M]^+$.

3.2. General procedure for the preparation of the electroactive dyads derivatives **9-10a,b** (H_2P-n-*m*CHO, H_2P-n-*p*CHO) y **12-13a,b** (ZnP-n-*m*CHO, ZnP-n-*p*CHO).

The corresponding electroactive dyads **9-10a,b** y **12-13a,b** were obtained from iodotetraphenylphorpyrins **7a**, **7b** and the oligovinylpyridines **2a,b** and **5a,b** by palladium-catalyzed Heck cross coupling reactions, (Scheme SS1). A mixture of porphyrin **7a** or **7b** (1 eq), the corresponding vinylpyridine oligomer **2a,b** or **5a,b** (1.5 eq), palladium acetate $Pd(OAc)_2$ (0.1 eq), tetrabutylammonium bromide Bu_4NBr (3 eq) and K_2CO_3 (3 eq) in 20 mL of DMF was stirred vigorously by argon bubbled directly into the solution for 30 minutes. The reaction mixture was refluxed for 30 hours under inert atmosphere, cooled to room temperature and the solvent was removed, then water was added and extracted with CH_2Cl_2 (3x40 mL), the organic extracts were dried over anhydrous magnesium sulfate ($MgSO_4$) and the solvent was removed under vacuum. The crudes were purified by column chromatography (silica gel) using hexanes/toluene (7/3) and dichloromethane/hexanes (1/1) mixtures as eluents to give the desired products **9-10a,b** and **12-13a,b** in moderate yields.

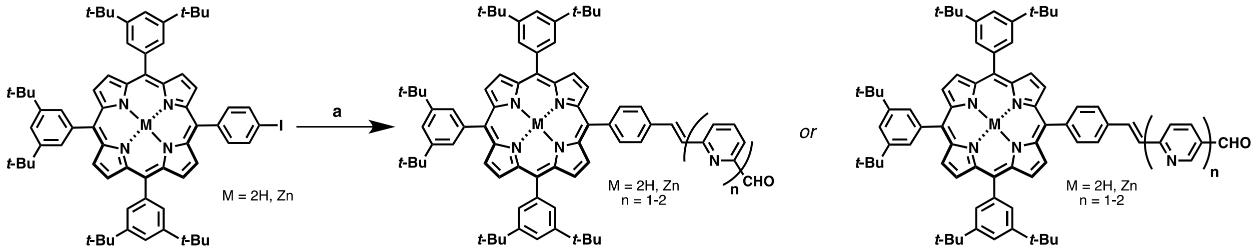

7a (H_2P) **7b** (ZnP)

9a (H_2P-1-*m*CHO) **12a** (ZnP-1-*m*CHO)
10a (H_2P-2-*m*CHO) **13a** (ZnP-2-*m*CHO)

9b (H_2P-1-*m*CHO) **12b** (ZnP-1-*m*CHO)
10b (H_2P-2-*m*CHO) **13b** (ZnP-2-*m*CHO)

Scheme S1. Synthetic route for the preparation of compounds **9-10a,b** (H_2P-n-*m*CHO, H_2P-n-*p*CHO) and **12-13a,b** (ZnP-n-*m*CHO, ZnP-n-*p*CHO). Reagents and conditions: a) **2a,b** or **5a,b,** $Pd(OAc)_2$, Bu_4NBr, K_2CO_3, DMF (anh), reflux, 30 h, yield = 50-65%.

3.3. General procedure for the preparation of the electroactive dyads derivatives **11a,b** (H_2P-3-*m*CHO, H_2P-3-*p*CHO) and **14a,b** (ZnP-3-*m*CHO, ZnP-3-*p*CHO).

The electroactive dyads **11a,b** and **14a,b** were obtained from phosphonatemethyltetraphenylphorpyrins **8a**, **8b** and the oligovinylpyridines **6a,b** by Horner-Emmons condensation reactions using tetrahydrofurane as solvent, (Scheme S2). To a solution of phosphonatetetraphenylporphyrin **8a** or **8b** (1eq) and the corresponding vinylpyridine oligomer **6a,b** (2 eq) in 20 mL of anhydrous tetrahydrofurane, at reflux and under argon atmosphere were added slowly potasium *tert*-butoxide (1.2 eq). The reaction mixture was allowed to reflux for 20 hours, then allowed to cool to room temperature and methanol and water was added, after phase separation, the aqueous phase was extracted with $CHCl_3$ (3x40 mL), the combined organic extracts were dried over anhydrous magnesium sulfate ($MgSO_4$) and the solvent was distilled under vacuum. The crudes were purified by column chromatography (silica gel) using hexanes and ethyl acetate (1/1) mixture as eluent to give the desired products **11a,b** and **14a,b** in moderate yields.

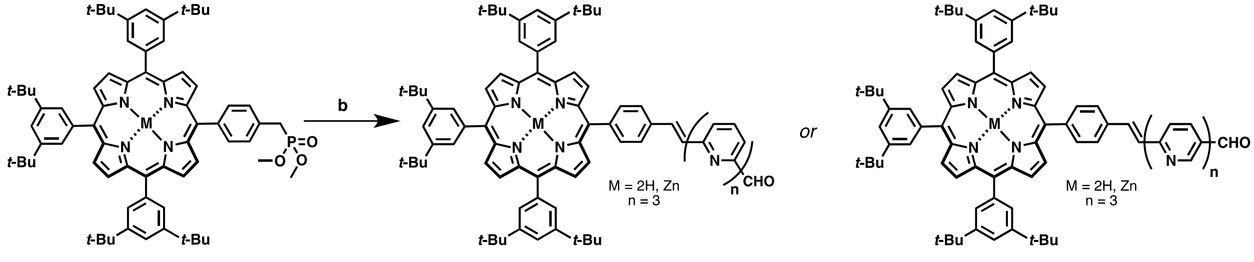

8a (H_2P) **8b** (ZnP)

11a (H_2P-3-*m*CHO) **14a** (ZnP-3-*m*CHO)

11b (H_2P-3-*m*CHO) **14b** (ZnP-3-*m*CHO)

Scheme S2. Synthetic route for the preparation of the compounds **11a,b** (H_2P-3-*m*CHO, H_2P-3-*p*CHO) and **14a,b** (ZnP-3-*m*CHO, ZnP-3-*p*CHO). Reagents and conditions: b) **6a** or **6b**, $Pd(OAc)_2$, Bu_4NBr, K_2CO_3, DMF (anh), reflux, 30 h, yield = 42-51%.

3.4 General procedure for the preparation and spectroscopic data for the new porphyrin-fullerene conjugates **15-20a** (H_2P-n-*m*C_{60}, ZnP-n-*m*C_{60}, n=1-3) and **15-20b** (H_2P-n-*p*C_{60}, ZnP-n-*p*C_{60}, n=1-3).

The corresponding electroactive dyads **9-14a,b** (1eq), fullerene C_{60} (2eq) and *N*-octylglycine (3eq) were dissolved in toluene anhydrous and the mixture was refluxed for 4-5 hours under inert atmosphere. The reaction mixture was allowed to reach room temperature and the solvent was removed under vacuum. The crude was purified by column chromatography on SiO_2 using as eluent CS_2, and a mixtures of toluene:CS_2 (1:1; 2:1; 3:1), respectively to obtained the desired compounds **15-20a,b** in moderate yields (30-45%).

Compound 15a (H_2P-1-*m*C_{60}): Yield (32 %). ^{1}H-NMR (600 MHz, $CDCl_3$) δ_H = -2.71 (s, 2H), 0.92 (t, *J* = 6.4 Hz, 3H), 1.25-1.54 (m, 64H), 1.86-2.01 (m, 2H), 2.65-268 (m, 1H), 3.24-3.28 (m, 1H), 3.99 (d, *J* = 10.1 Hz, 1H), 4.95 (d, *J* = 10.1 Hz, 1H), 5.27 (s, 1H), 7.15 (d, *J* = 7.3 Hz, 1H), 7.41-7.43 (m, 2H), 7.75-7.84 (m, 6H), 7.87 (d, *J* = 8.2 Hz, 2H), 8.07-8.09 (m, 6H), 8.18 (d, *J* = 7.3 Hz, 2H), 8.83-8.88 (m, 8H) ppm; ^{13}C-NMR (150 MHz, $CDCl_3$) δ_C = 14.1, 14.2, 21.5, 22.8, 27.7, 28.5, 29.4, 29.7, 30.9, 31.6, 31.8, 31.9, 32.0, 35.1, 53.6, 67.0, 68.0, 68.9, 75.6, 83.1, 119.2, 121.0, 121.2, 121.5, 121.7, 122.1 125.3, 125.5, 128.3, 128.6, 129.1, 129.8, 133.0, 134.9, 135.3, 135.9, 136.0, 136.2, 136.6, 137.1, 139.0, 139.3, 139.6, 139.7, 139.8, 141.0, 141.2, 141.3, 141.4, 141.5, 141.6, 141.7, 141.8, 141.9, 142.6, 142.7, 143.7, 143.9, 144.3, 144.4, 144.5, 144.9, 145.0, 145.1, 145.2, 145.3, 145.4, 145.7, 145.8, 146.3, 146.8, 148.7, 152.9, 153.6, 154.8, 155.1, 156.1, 158.1 ppm; FTIR (KBr) υ = 2959, 2922, 2854, 1586, 1445, 1261, 800, 527 cm^{-1}; MS (MALDI-TOF): *m/z* 1209 [M-C_{60}]$^+$.

Compound 15b (H_2P-1-*p*C_{60}): Yield (31 %). ^{1}H-NMR (600 MHz, $CDCl_3$) δ_H = -2.71 (s, 2H), 0.93 (t, *J* = 7.3 Hz, 3H), 1.25-1.54 (m, 64H), 1.84-1.96 (m, 2H), 2.50-2.53 (m, 1H), 3.11-3.15 (m, 1H), 3.86 (d, *J* = 8.2 Hz, 1H), 4.81 (s, 1H), 4.91 (d, *J* = 8.2 Hz, 1H), 7.49 (d, *J* = 16.5 Hz, 1H), 7.55 (d, *J* = 7.3 Hz, 1H), 7.78-7.79 (m, 3H), 7.96 (d, *J* = 7.3 Hz, 2H), 8.02 (d, *J* = 16.5 Hz, 1H), 8.07-8.09 (m, 6H), 8.16 (d, *J* = 8.2 Hz, 2H), 8.85-8.97 (m, 9H) ppm; ^{13}C-NMR (150 MHz, $CDCl_3$) δ_C = 14.1, 14.2, 22.7, 22.8, 23.2, 26.8, 27.6, 28.3, 29.0, 29.4, 29.7, 29.8, 30.1, 30.2, 30.9, 31.8, 32.0, 32.1, 33.5. 33.8, 35.1, 53.3, 53.4, 66.8, 68.5, 76.1, 79.8, 119.2, 121.0, 121.6, 121.7, 125.6, 128.2, 129.7, 129.8, 131.7, 133.1, 134.8, 134.9, 135.1, 135.8, 135.9, 136.8, 137.5, 139.0, 139.4, 139.5, 140.7, 140.8, 141.0, 141.1, 141.2, 141.4, 141.5, 141.6, 141.7, 142.2, 142.8, 143.2, 143.7, 144.1, 144.4, 144.6, 144.9, 145.0, 145.1, 145.2, 145.3, 145.4, 145.6, 146.2, 146.3, 148.8, 150.7, 152.0, 152.1, 153.1, 155.6, 155.9 ppm; FTIR (KBr) υ = 2958, 2922, 2851, 1591, 1464, 1362, 1247, 800, 527 cm^{-1}; MS (MALDI-TOF): *m/z* 1210.25 [M-C_{60}]$^+$.

Compound 16a (H_2P-2-*m*C_{60}): Yield (35 %). ^{1}H-NMR (600 MHz, $CDCl_3$) δ_H = -2.70 (s, 2H), 0.91 (t, *J* = 6.4 Hz, 3H), 1.25-1.54 (m, 64H), 1.87-2.03 (m, 2H), 2.66-2.71 (m, 1H), 3.24-3.29 (m, 1H), 4.11 (d, *J* = 9.1 Hz, 1H), 5.05 (d, *J* = 9.1 Hz, 1H), 5.33 (s, 1H), 7.29 (d, *J* = 8.2 Hz, 1H), 7.39 (d, *J* = 7.3 Hz, 1H), 7.45-7.48 (m, 2H), 7.65 (d, *J* = 15.6 Hz, 1H), 7.67-7.69 (m, 1H), 7.78-7.80 (m, 4H), 7.84 (d, *J* = 15.6 Hz, 1H), 7.91 (d, *J* = 7.3 Hz, 1H), 7.96 (d, *J* = 8.2 Hz, 1H), 8.03 (d, *J* = 16.5 Hz, 1H), 8.08-8.09 (m, 6H), 8.23 (d, *J* = 8.2 Hz, 2H), 8.88-8.89 (m, 8H) ppm; ^{13}C-NMR (150 MHz, $CDCl_3$) δ_C = 14.1, 14.2, 22.7, 28.5, 29.4, 29.7, 29.8, 30.9, 31.8, 32.0, 35.1, 53.6, 67.0, 69.0, 75.6, 83.1, 119.2, 121.0, 121.3, 121.5, 121.6, 122.1, 122.6, 125.5, 128.8, 129.7, 129.8, 132.1, 132.6, 132.9, 135.0, 135.4, 135.5, 136.3, 136.7, 137.1, 137.2, 139.1, 139.5, 139.8, 141.2, 141.3, 141.4, 141.5, 141.6, 141.7, 141.9, 142.0, 142.1, 142.2, 142.4, 142.6, 142.7, 143.8, 144.0, 144.1, 144.5, 144.6, 144.7, 144.8, 144.9, 145.0, 145.1, 145.2, 145.3, 145.4, 145.5, 145.6, 145.7, 145.8, 146.0, 146.6, 146.9, 148.8, 152.9, 153.7, 153.8, 154.7, 155.0, 155.6, 156.1, 158.1 ppm; FTIR (KBr) υ = 2958, 2924, 2854, 1640, 1590, 1452, 1362, 1247, 800, 527 cm^{-1}; MS (MALDI-TOF): *m/z* 1312 [M-C_{60}]$^+$.

Compound 16b (H_2P-2-*p*C_{60}): Yield (38 %). ^{1}H-NMR (600 MHz, $CDCl_3$) δ_H = -2.68 (s, 2H), 0.91 (t, *J* = 6.4 Hz, 3H), 1.26-1.52 (m, 64H), 1.82-1.97 (m, 2H), 2.51-2.55 (m, 1H), 3.11-3.16 (m, 1H), 3.87 (d, *J* = 10.1 Hz, 1H), 4.89 (d, *J* = 10.1 Hz, 1H), 4.90 (s, 1H), 7.32 (d, *J* = 8.2 Hz, 1H), 7.37 (d, *J* = 7.3 Hz, 1H), 7.48 (d, *J* = 16.5 Hz, 1H), 7.58 (d, *J* = 8.2 Hz, 1H), 7.69 (t, *J* = 7.3 Hz, 1H), 7.75-7.78 (m, 4H), 7.90 (d, *J* = 15.6 Hz, 1H), 7.98 (d, *J* = 7.3 Hz, 2H), 8.04-8.08 (m, 8H), 8.23 (d, *J* = 8.2 Hz, 2H), 8.88-8.97 (m, 9H) ppm; ^{13}C-NMR (150 MHz, $CDCl_3$) δ_C = 14.1, 14.2, 22.7, 26.8, 27.6, 28.3, 29.4, 29.7, 29.8, 31.8, 32.0, 35.1, 53.3, 59.0, 66.9, 68.6, 76.3, 79.0, 81.9, 119.2, 121.0, 121.4, 121.5, 121.7, 121.9, 125.5, 128.8, 129.7, 129.8, 131.8, 132.2, 132.6, 132.9, 135.0, 135.1, 135.6, 136.0, 136.1, 137.0, 137.1, 137.5, 139.3, 139.6, 139.7, 139.8, 141.0, 141.2, 141.3, 141.4, 141.5, 141.6, 141.7, 141.9, 142.0, 142.3, 142.6, 142.7, 143.7, 143.8, 144.1, 144.2, 144.5, 144.6, 144.9, 145.0, 145.1, 145.2, 145.3, 145.4, 145.5, 145.6, 145.8, 145.7, 145.8, 145.9, 146.6, 148.8, 150.7, 152.2, 152.5, 153.3, 154.8, 155.5, 155.6, 155.9 ppm; FTIR (KBr) υ = 2957, 2923, 2853, 1632, 1591, 1464, 1363, 1247, 800, 527 cm^{-1}; MS (MALDI-TOF): *m/z* 1313 [M-C_{60}]$^+$.

Compound 17a (H_2P-3-*m*C_{60}): Yield (30 %). ^{1}H-NMR (600 MHz, $CDCl_3$) δ_H = -2.74 (s, 2H), 0.84 (t, *J* = 6.4 Hz, 3H), 1.19-1.57 (m, 64H), 1.81-1.92 (m, 2H), 2.60-2.62 (m, 1H), 3.13-3.18 (m, 1H), 4.02 (d, *J* = 9.1 Hz, 1H), 4.95 (d, *J* = 10.1 Hz, 1H), 5.24 (s, 1H), 7.22 (d, *J* = 8.2 Hz, 1H), 7.33 (d, *J* = 8.2 Hz, 1H), 7.36-7.46 (m, 4H), 7.53 (d, *J* = 16.5 Hz, 1H), 7.61 (t, *J* = 7.3 Hz, 1H), 7.66-7.79 (m, 7H), 7.83 (d, *J*

= 8.26 Hz, 1H), 7.87 (d, *J* = 15.6 Hz, 1H), 7.95 (d, *J* = 7.3 Hz, 2H), 8.02-8.06 (m, 7H), 8.19 (d, *J* = 8.2 Hz, 2H), 8.84-8.86 (m, 8H) ppm; ^{13}C-NMR (150 MHz, $CDCl_3$) δ_C = 14.1, 14.2, 19.5, 22.7, 27.7, 28.5, 29.4, 29.7, 29.8, 31.8, 32.0, 35.1, 53.6, 66.9, 68.9, 75.5, 83.1, 83.2, 119.3, 119.4, 120.4, 120.5, 121.0, 121.2, 121.5, 121.6, 121.8, 121.9, 122.0, 122.1, 122.6, 122.7, 123.1, 123.2, 125.6, 125.7, 128.8, 129.0, 129.7, 129.8, 131.9, 132.3, 132.4, 132.5, 133.0, 135.0, 135.2, 135.3, 136.1, 136.6, 137.1, 137.2, 137.4, 139.0, 139.4, 139.5, 139.6, 140.9, 141.1, 141.3, 141.4, 141.7, 141.8, 141.9, 142.0, 142.5, 142.6, 142.7, 142.8, 143.3, 143.6, 143.9, 144.0, 144.2, 144.6, 144.8, 145.0, 145.1, 145.2, 145.5, 145.6, 146.2, 146.3, 146.7, 146.8, 148.7, 152.8, 153.5, 153.6, 153,7, 154.3, 154.5, 154.7, 154.9, 155.2, 155.6, 155.7, 156.0, 156.2, 158.1, 158.2 ppm; FTIR (KBr) υ = 2956, 2923, 2850, 1635, 1590, 1465, 1260, 1246, 800, 527 cm^{-1}; MS (MALDI-TOF): *m/z* 1415 [M-C_{60}]$^+$.

Compound 17b (H_2P-3-*p*C_{60}): Yield (37 %). ^{1}H-NMR (600 MHz, $CDCl_3$) δ_H = -2.69 (s, 2H), 0.92 (t, *J* = 6.4 Hz, 3H), 1.25-1.62 (m, 84H), 1.83-1.95 (m, 3H), 2.51-2.55 (m, 1H), 3.11-3.16 (m, 1H), 3.90 (d, *J* = 10.1 Hz, 1H), 4.89 (s, 1H), 4.94 (d, *J* = 8.2 Hz, 1H), 7.31 (d, *J* = 7.3 Hz, 1H), 7.35 (d, *J* = 7.3 Hz, 1H), 7.40 (d, *J* = 16.5 Hz, 1H), 7.51 (d, *J* = 16.5 Hz, 1H), 7.55-7.58 (m, 2H), 7.68 (t, *J* = 7.3 Hz, 1H), 7.73 (d, *J* = 15.6 Hz, 2H), 7.78-7.80 (m, 3H), 7.87 (d, *J* = 7.3 Hz, 2H), 7.91 (d, *J* = 8.2 Hz, 2H), 8.00 (d, *J* = 8.2 Hz, 1H), 8.07-8.08 (m, 7H), 8.22 (d, *J* = 7.3 Hz, 2H), 8.87-8.89 (m, 9H) ppm; ^{13}C-NMR (150 MHz, $CDCl_3$) δ_C = 14.1, 14.2, 22.7, 22.8, 27.6, 28.3, 29.4, 29.7, 29.8, 31.7, 31.8, 32.0, 35.1, 53.3, 66.9, 68.7, 76.3, 119.1, 121.0, 121.5, 121.7, 122.4, 122.5, 123.1, 125.1, 125.5, 129.7, 129.8, 130.5, 131.9, 132.0, 132.2, 132.3, 133.2, 135.0, 135.1, 135.6, 136.1, 137.0, 137.2, 137.5, 139.3, 139.5, 139.6, 139.8, 139.9, 141.1, 141.2, 141.4, 141.5, 141.6, 141.7, 141.8, 141.9, 142.0, 142.1, 142.4, 142.6, 142.7, 143.8, 143.9, 144.1, 144.2, 144.6, 144.7, 144.9, 145.0, 145.1, 145.3, 145.4, 145.5, 145.6, 145.7, 145.8, 145.9, 146.6, 148.8, 147.1, 148.7, 148.8, 149.0, 150.7, 150.8, 152.2, 152.5, 152.6, 153.4, 153.5, 154.3, 154.8, 155.0, 155.9 ppm; FTIR (KBr) υ = 2957, 2924, 2853, 1634, 1591, 1465, 1259, 1246, 800, 526 cm^{-1}; MS (MALDI-TOF): *m/z* 1416 [M-C_{60}]$^+$.

Compound 18a (ZnP-1-*m*C_{60}): Yield (38 %). ^{1}H-NMR (600 MHz, $CDCl_3$) δ_H = 0.93 (t, *J* = 6.42 Hz, 3H), 1.25-1.52 (m, 64H), 1.85-1.97 (m, 2H), 2.61-2.65 (m, 1H), 3.20-3.25 (m, 1H), 3.81 (d, *J* = 9.17 Hz, 1H), 4.81 (d, *J* = 10.1 Hz, 1H), 5.17 (s, 1H), 7.40 (d, *J* = 6.4 Hz, 1H), 7.41 (d, *J* = 16.5 Hz, 1H), 7.73-7.80 (m, 6H), 7.86 (d, *J* = 7.3 Hz, 2H), 8.07-8.09 (m, 6H), 8.18 (d, *J* = 8.26 Hz, 2H), 8.92-8.99 (m, 8H) ppm; ^{13}C-NMR (150 MHz, $CDCl_3$) δ_C = 14.1, 14.2, 22.8, 27.7, 28.5, 29.4, 29.7, 31.5, 31.8, 32.0, 35.1, 53.6, 66.8, 68.7, 75.4, 83.0, 120.2, 120.8, 120.9, 121.2, 122.0, 122.5, 122.7, 125.4, 128.5, 129.6, 129.7, 129.8, 131.6, 132.2, 132.3, 132.4, 133.1, 134.8, 134.9, 135.1, 135.8, 136.0, 136.5, 137.1, 138.8, 139.1, 139.2, 139.4, 140.7, 140.9, 141.0, 141.1, 141.2, 141.3, 141.4, 141.5, 141.6, 141.7, 141.8, 142.0, 142.1, 142.3, 143.1, 143.4, 143.5, 143.9, 144.0, 144.1, 144.3, 144.6, 144.7, 144.9, 145.0, 145.1, 145.3, 145.4, 145.5, 146.0, 146.1, 146.7, 148.6, 150.0, 150.4, 150.5, 150.6, 152.8, 153.4, 153.5, 155.1, 155.9, 158.0 ppm; FTIR (KBr) υ = 2957, 2924, 2855, 1589, 1455, 1361, 1247, 797, 527 cm^{-1}; MS (MALDI-TOF): *m/z* 1271 [M-C_{60}]$^+$.

Compound 18b (ZnP-1-*p*C_{60}): Yield (34 %). ^{1}H-NMR (600 MHz, $CDCl_3$) δ_H = 0.93 (t, *J* = 6.4 Hz, 3H), 1.25-1.62 (m, 64H), 1.80-1.95 (m, 2H), 2.46-2.51 (m, 1H), 3.08-3.12 (m, 1H), 3.70 (d, *J* = 8.2 Hz, 1H), 4.62 (s, 1H), 4.80 (d, *J* = 9.1 Hz, 1H), 7.48 (d, *J* = 16.5 Hz, 1H), 7.52 (d, *J* = 8.2 Hz, 1H), 7.78-7.79 (m, 3H), 7.96 (d, *J* = 8.2 Hz, 2H), 8.02 (d, *J* = 15.6 Hz, 1H), 8.06-8.09 (m, 6H), 8.15 (d, *J* = 8.2 Hz, 2H), 8.94-9.00 (m, 9H) ppm; ^{13}C-NMR (150 MHz, $CDCl_3$) δ_C = 14.1, 14.2, 22.8, 27.6, 28.3, 29.4, 29.7, 31.9, 32.0, 35.1, 53.2, 66.6, 68.3, 75.9, 79.6, 120.2, 120.8, 122.0, 122.6, 122.7, 125.5, 128.1, 129.6, 129.7, 131.5, 131.6, 132.2, 132.3, 132.4, 133.3, 134.4, 134.6, 134.8, 135.5, 135.7, 136.6, 137.4, 138.9, 139.1, 139.2, 140.5, 140.6, 140.7, 140.8, 140.9, 141.0, 141.1, 141.2, 141.3, 141.4, 141.5, 141.6, 141.8, 141.9, 142.0, 142.1, 142.9, 143.3, 143.4, 143.5, 143.7, 143.9, 144.0, 144.1, 144.2, 144.4, 144.5, 144.6, 144.7, 144.8, 144.9, 145.0, 145.1, 145.3, 145.4, 145.5, 146.1, 146.2, 148.6, 149.9, 150.4, 150.5, 150.6, 151.9, 152.0, 152.9, 155.5, 155.9, 156.0 ppm; FTIR (KBr) υ = 2957, 2925, 2858, 1590, 1466, 1426, 1361, 1246, 797, 527 cm^{-1}; MS (MALDI-TOF): *m/z* 1272 [M-C_{60}]$^+$.

Compound 19a (ZnP-2-*m*C_{60}): Yield (34 %). ^{1}H-NMR (600 MHz, $CDCl_3$) δ_H = 0.91 (t, *J* = 6.4 Hz, 3H), 1.25-1.62 (m, 64H), 1.86-2.00 (m, 2H), 2.63-2.67 (m, 1H), 3.21-3.25 (m, 1H), 3.95 (d, *J* = 8.2 Hz, 1H), 4.93 (d, *J* = 9.1 Hz, 1H), 5.22 (s, 1H), 7.27 (d, *J* = 7.3 Hz, 2H), 7.38 (d, *J* = 8.2 Hz, 1H), 7.44-7.46 (m, 2H), 7.62 (d, *J* = 15.6 Hz, 1H), 7.67 (t, *J* = 7.3 Hz, 1H), 7.75-7.78 (m, 4H), 7.83 (d, *J* = 15.6 Hz, 2H), 7.95 (d, *J* = 7.3 Hz, 2H), 8.02 (d, *J* = 16.5 Hz, 1H), 8.08-809 (m, 6H), 8.22 (d, *J* = 8.2 Hz, 2H), 8.98-8.99 (m, 8H) ppm; ^{13}C-NMR (150 MHz, $CDCl_3$) δ_C = 14.1, 14.2, 22.7, 22.8, 27.7, 28.5, 29.4, 29.7, 29.8, 31.8, 31.9, 32.0, 35.1, 53.6, 66.9, 68.9, 73.4, 75.6, 83.1, 120.3, 120.8, 120.9, 121.2, 122.1, 122.5, 122.6, 122.7, 125.4, 128.7, 129.6, 129.7, 131.6, 132.1, 132.2, 132.3, 132.4, 132.7, 133.0, 134.8, 135.3, 135.9, 136.2, 136.6, 137.1, 137.2, 139.0, 139.4, 139.6, 141.1, 141.2, 141.3, 141.4, 141.5, 141.6, 141.7, 141.8, 141.9, 142.0, 142.3, 142.5, 143.3, 143.7, 143.9, 144.0, 144.1, 144.4, 144.5, 144.9, 145.0, 145.1, 145.2, 145.3, 145.4, 145.5, 145.6, 145.7, 145.8, 145.9, 146.5, 146.6, 146.8, 148.6, 150.0, 150.4, 150.5, 150.6, 152.9, 153.7, 153.8, 154.7, 154.9, 155.7, 156.1, 158.0 ppm; FTIR (KBr) υ = 2957, 2923, 2853, 1589, 1452, 1426, 1361, 1247, 796, 527 cm^{-1}; MS (MALDI-TOF): *m/z* 1375 [M-C_{60}]$^+$.

Compound 19b (ZnP-2-*p*C_{60}): Yield (45 %). ^{1}H-NMR (600 MHz, $CDCl_3$) δ_H = 0.90 (t, *J* = 6.4 Hz, 3H), 1.24-1.62 (m, 64H), 1.83-1.95 (m, 2H), 2.50-2.54 (m, 1H), 3.10-3.14 (m, 1H), 3.79 (d, *J* = 9.1 Hz, 1H), 4.83 (d, *J* = 10.1 Hz, 1H), 4.84 (s, 1H), 7.32 (d, *J* = 7.3 Hz, 1H), 7.39 (d, *J* = 7.3 Hz, 1H), 7.48 (d, *J* = 15.6 Hz, 1H), 7.57 (d, *J* = 8.2 Hz, 1H), 7.69 (d, *J* = 7.3 Hz, 1H), 7.72 (d, *J* = 16.5 Hz, 1H), 7.76-7.77 (m, 4H), 7.88 (d, *J* = 15.6 Hz, 1H), 7.98 (d, *J* = 7.3 Hz, 2H), 8.05-8.06 (m, 8H), 8.22 (d, *J* = 8.2 Hz, 2H), 8.98-9.00 (m, 8H) ppm; ^{13}C-NMR (150 MHz, $CDCl_3$) δ_C = 14.1, 14.2, 22.7, 22.8, 23.4, 26.4, 27.6, 28.3, 29.4, 29.6, 29.7, 29.8, 31.4, 31.5, 31.6, 31.7, 31.8, 31.9, 32.0,

35.1, 53.2, 53.4, 66.7, 68.3, 76.1, 79.7, 79.8, 120.8, 121.4, 121.8, 122.5, 122.7, 122.8, 122.9, 123.8, 125.4, 128.7, 129.6, 129.7, 131.6, 131.7, 131.8, 131.9, 132.1, 132.2, 132.3, 132.4, 132.6, 133.0, 134.6, 134.7, 134.8, 135.2, 135.8, 135.9, 136.9, 137.1, 137.4, 139.0, 139.1, 139.3, 139.5, 140.6, 140.7, 140.9, 141.0, 141.1, 141.2, 141.3, 141.4, 141.5, 141.6, 141.7, 141.8, 142.0, 142.4, 143.4, 143.5, 143.9, 144.0, 144.2, 144.3, 144.4, 144.5, 144.6, 144.7, 144.9, 145.0, 145.1, 145.2, 145.3, 145.4, 145.5, 145.6, 145.7, 145.8, 146.3, 148.6, 148.8, 150.0, 150.3, 150.4, 150.5, 150.6, 150.7, 152.0, 152.2, 152.9, 154.7, 155.4, 155.5, 155.6, 155.7 ppm; FTIR (KBr) υ = 2957, 2923, 2853, 1591, 1474, 1361, 1247, 796, 527 cm^{-1}; MS (MALDI-TOF): *m/z* 1376 $[M-C_{60}]^+$.

Compound 20a (ZnP-3-*m*C_{60}): Yield (36 %). ^{1}H-NMR (600 MHz, $CDCl_3$) δ_H = 0.90 (t, *J* = 6.4 Hz, 3H), 1.24-1.52 (m, 64H), 1.84-2.04 (m, 2H), 2.63-2.65 (m, 1H), 3.16-3.21 (m, 1H), 3.97 (d, *J* = 9.1 Hz, 1H), 4.93 (d, *J* = 9.1 Hz, 1H), 5.24 (s, 1H), 7.27 (d, *J* = 7.3 Hz, 1H), 7.38 (d, *J* = 7.3 Hz, 1H), 7.42 (d, *J* = 7.3 Hz, 1H), 7.44 (d, *J* = 8.2 Hz, 1H), 7.47 (d, *J* = 7.3 Hz, 1H), 7.50 (d, *J* = 16.5 Hz, 1H), 7.57 (d, *J* = 15.6 Hz, 1H), 7.66 (t, *J* = 7.3 Hz, 1H), 7.72 (d, *J* = 7.3 Hz, 1H), 7.74 (d, *J* = 8.2 Hz, 1H), 7.76-7.78 (m, 4H), 7.81 (d, *J* = 7.3 Hz, 1H), 7.84 (d, *J* = 7.3 Hz, 1H), 7.92 (d, *J* = 16.5 Hz, 1H), 7.99 (d, *J* = 7.3 Hz, 2H), 8.07-8.11 (m, 7H), 8.24 (d, *J* = 7.3 Hz, 2H), 8.99-9.00 (m, 8H) ppm; ^{13}C-NMR (150 MHz, $CDCl_3$) δ_C = 14.1, 14.2, 22.7, 22.8, 23.2, 24.7, 26.0, 26.7, 27.6, 28.5, 29.0, 29.4, 29.5, 29.6, 29.7, 29.8, 30.1, 30.2, 31.7, 31.8, 31.9, 32.0, 33.5, 33.8, 34.4, 35.1, 36.3, 36.7, 37.1, 37.4, 53.5, 66.9, 68.9, 75.6, 83.2, 120.1, 120.4, 120.8, 121.2, 121.4, 121.6, 121.7, 121.8, 122.1, 122.6, 122.7, 122.8, 125.5, 128.6, 129.6, 129.7, 129.8, 131.7, 132.2, 132.3, 132.4, 132.6, 133.1, 134.8, 134.4, 135.9, 136.2, 136.7, 137.1, 137.2, 139.1, 139.4, 139.5, 139.7, 141.0, 141.3, 141.4, 141.5, 141.6, 141.7, 141.8, 141.9, 142.0, 142.7, 143.2, 143.3, 143.4, 143.8, 144.1, 144.2, 144.3, 144.4, 144.5, 144.6, 144.8, 144.9, 145.0, 145.1, 145.2, 145.3, 145.4, 145.5, 145.7, 145.8, 145.9, 146.5, 146.6, 146.7, 146.8, 148.6, 158.7, 150.0, 150.4, 150.5, 150.6, 152.9, 153.4, 153.6, 153.7, 154.8, 154.9, 155.2, 155.6, 156.1, 158.0 ppm; FTIR (KBr) υ = 2957, 2923, 2852, 1586, 1453, 1363, 1260, 797, 527 cm^{-1}; MS (MALDI-TOF): *m/z* 1478 $[M-C_{60}]^+$.

Compound 20b (ZnP-3-*p*C_{60}): Yield (42 %). ^{1}H-NMR (600 MHz, $CDCl_3$) δ_H = 0.92 (t, *J* = 6.4 Hz, 3H), 1.24-1.53 (m, 84H), 1.85-2.00 (m, 3H), 2.55-2.61 (m, 1H), 3.16-3.18 (m, 1H), 4.02 (d, *J* = 9.2 Hz, 1H), 5.02 (d, *J* = 10.1 Hz, 1H), 5.09 (s, 1H), 7.31 (d, *J* = 8.2 Hz, 1H), 7.34 (d, *J* = 7.3 Hz, 1H), 7.38-7.45 (m, 2H), 7.51 (d, *J* = 16.5 Hz, 1H), 7.54-7.60 (m, 2H), 7.73 (d, *J* = 15.6 Hz, 1H), 7.68 (t, *J* = 7.3 Hz, 1H), 7.77-7.78 (m, 3H), 7.82-7.86 (m, 2H), 7.92 (d, *J* = 8.2 Hz, 2H), 7.99 (d, *J* = 7.3 Hz, 1H), 8.08-8.09 (m, 6H), 8.23 (d, *J* = 7.3 Hz, 2H), 8.80-9.00 (m, 8H) ppm; ^{13}C-NMR (150 MHz, $CDCl_3$) δ_C = 14.1, 14.3, 22.7, 22.8, 23.8, 27.6, 27.7, 28.4, 28.5, 29.4, 29.8, 29.9, 31.4, 31.5, 31.6, 31.9, 32.0, 34.0, 35.1, 53.5, 66.4, 68.0, 74.2, 74.3, 100.0, 120.1, 120.2, 120.4, 120.8, 122.6, 122.7, 122.8, 122.9, 123.1, 125.0, 125.3, 129.5, 129.6, 129.7, 129.8, 129.9, 130.0, 130.1, 130.5, 130.6, 130.7, 131.6, 131.8, 131.9, 131.9, 132.0, 132.1, 132.3, 132.4, 132.5, 132.6, 132.7, 132.8, 132.9, 133.0, 133.5, 134.9, 137.1, 137.2, 137.5, 137.6, 139.7, 139.8, 140.0, 140.1, 141.1, 141.2, 141.3, 141.4, 141.5, 141.6, 141.7, 141.8, 141.9, 142.1, 142.2, 143.1, 143.8, 144.5, 144.8, 145.0, 145.2, 145.5, 145.6, 145.7, 146.5, 146.6, 146.7, 148.6, 148.7, 149.5, 149.6, 149.8, 150.0, 150.1, 150.4, 150.5, 150.6, 150.7, 150.8, 152.0, 152.1, 152.9, 153.4, 153.6, 154.7, 154.8, 155.2, 155.7, 156.1, 158.1 ppm; FTIR (KBr) υ = 2956, 2924, 2854, 1590, 1473, 1361, 1247, 796, 526 cm^{-1}; MS (MALDI-TOF): *m/z* 1479 $[M-C_{60}]^+$.

4. Characterization

4.1 ^{1}H-NMR spectra and spectroscopic data for the compounds **9-14a,b**

Compound 9a (H_2P-1-*m*CHO): Yield (58 %). ^{1}H-NMR (600 MHz, $CDCl_3$) δ_H = -2.68 (s, 2H), 1.52 (s, 54H), 7.51 (d, *J* = 16.5 Hz, 1H), 7.70 (d, *J* = 8.3 Hz, 1H), 7.78-7.80 (m, 3H), 7.87-7.91 (m, 2H), 7.99 (d, *J* = 7.3 Hz, 2H), 8.08-8.11 (m, 7H), 8.27 (d, *J* = 7.3 Hz, 2H), 8.87-8.91 (m, 8H), 10.21 (s, 1H) ppm; ^{13}C-NMR (150 MHz, $CDCl_3$) δ_C = 29.8, 31.8, 35.2, 119.0, 119.8, 119.9, 121.1, 121.6, 121.7, 125.7, 125.9, 127.5, 129.8, 129.9, 134.4, 135.1, 135.7, 137.6, 137.7, 141.4, 141.5, 143.2, 148.8, 148.9, 152.9, 156.5, 156.6, 193.9 ppm; FTIR (KBr) υ = 3309, 3057, 2960, 2906, 2827, 1705, 1586, 1474, 1362 cm^{-1}; MS (MALDI-TOF): *m/z* 1082 $[M+H]^+$.

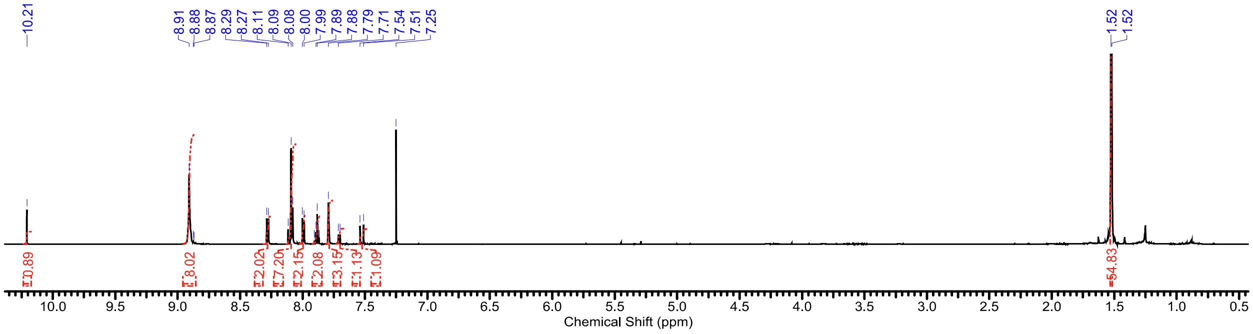

Figure S15. ^{1}H-NMR ($CDCl_3$, 600 MHz, 298 K) of compound **9a.**

Compound 9b (H_2P-1-*p*CHO): Yield (61 %). ^{1}H-NMR (600 MHz, $CDCl_3$) δ_H = -2.68 (s, 2H), 1.53 (s, 54H), 7.50 (d, *J* = 16.0, 1H), 7.59-7.61 (m, 1H), 7.79-7.80 (m, 3H), 8.00 (d, *J* = 8.3 Hz, 2H), 8.08-8.09 (m, 6H), 8.16-8.19 (m, 2H), 8.28 (d, *J* = 8.3 Hz, 2H), 8.89-8.91 (m, 8H), 9.12 (s, 1H), 10.12 (s, 1H) ppm; ^{13}C-NMR (150 MHz, $CDCl_3$) δ_C = 29.8, 31.8, 35.1, 118.8, 121.1, 121.6, 121.8, 122.5, 126.0, 127.5, 129.7, 129.8, 129.9, 135.2, 135.4, 136.5, 136.7, 141.3, 141.4, 143.8, 148.8, 148.9, 152.7, 160.8, 190.2 ppm; FTIR (KBr) υ = 3312, 3068, 2960, 2904, 2827, 1702, 1585, 1474, 1361 cm^{-1}; MS (MALDI-TOF): *m/z* 1082 [M+H]$^+$.

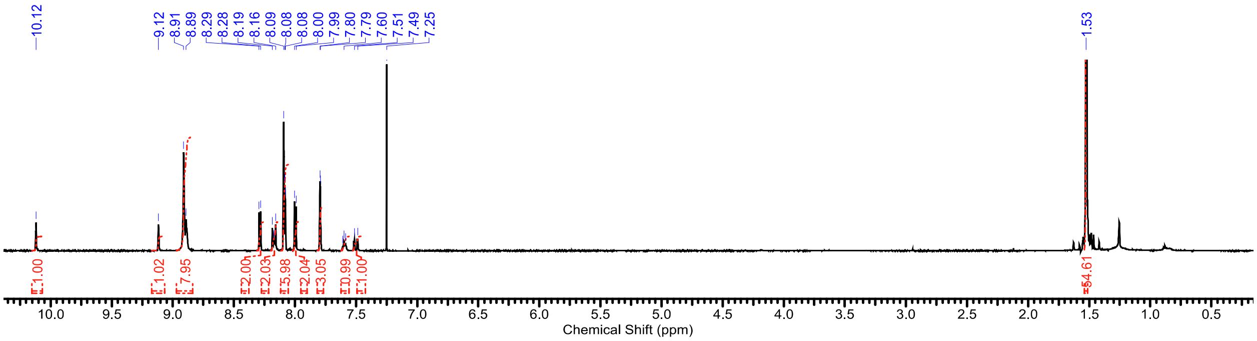

Figure S16. ^{1}H-NMR ($CDCl_3$, 600 MHz, 298 K) of compound **9b.**

Compound 10a (H_2P-2-*m*CHO): Yield (50 %). ^{1}H-NMR (600 MHz, $CDCl_3$) δ_H = -2.68 (s, 2H), 1.52 (s, 54H), 7.42 (d, *J* = 7.3 Hz, 1H), 7.48 (d, *J* = 7.3 Hz, 1H), 7.53 (d, *J* = 15.6 Hz, 1H), 7.75-7.76 (m, 1H), 7.77 (d, *J* = 7.34 Hz, 1H), 7.78-7.79 (m, 3H), 7.86-7.91 (m, 2H), 7.94 (d, *J* = 12.8 Hz, 1H), 8.01 (d, *J* = 8.3 Hz, 2H), 8.07-8.10 (m, 7H), 8.28 (d, *J* = 8.3 Hz, 2H), 8.87-9.02 (m, 8H), 10.17 (s, 1H) ppm; ^{13}C-NMR (150 MHz, $CDCl_3$) δ_C = 29.8, 31.8, 35.1, 114.6, 115.1, 119.2, 120.3, 121.1, 121.5, 121.7, 121.8, 122.0, 125.6, 126.8, 127.3, 128.8, 129.8, 129.9, 131.0, 133.2, 133.9, 135.1, 136.1, 137.3, 137.7, 141.4, 142.8, 148.8, 152.9, 154.6, 155.9, 156.1, 193.9 ppm; FTIR (KBr) υ = 3311, 2958, 2915, 2859, 1713, 1588, 1474, 1362 cm^{-1}; MS (MALDI-TOF): *m/z* 1185 [M+H]$^+$.

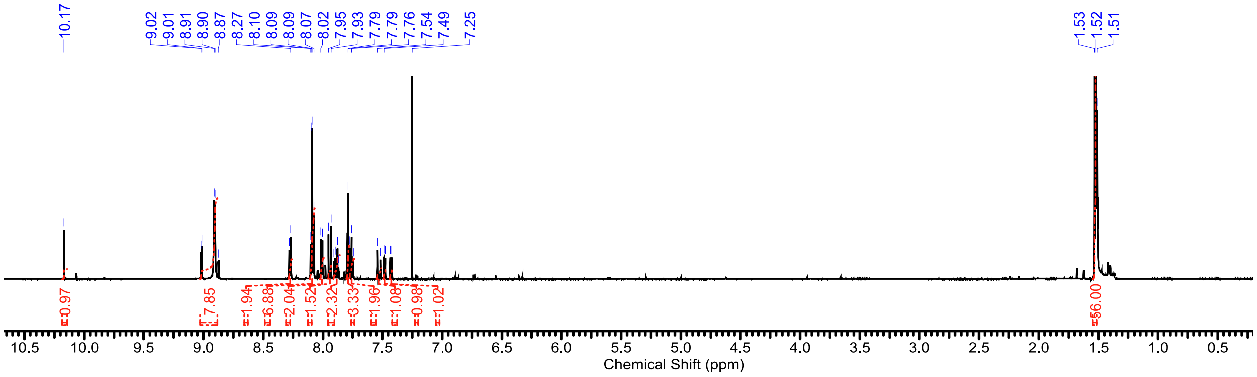

Figure S17. ^{1}H-NMR ($CDCl_3$, 600 MHz, 298 K) of compound **10a.**

Compound 10b (H_2P-2-*p*CHO): Yield (55 %). ^{1}H-NMR (600 MHz, $CDCl_3$) δ_H = -2.72 (s, 2H), 1.49 (s, 54H), 7.37 (d, *J* = 7.3 Hz, 1H), 7.45 (d, *J* = 7.3 Hz, 1H), 7.48 (d, *J* = 15.6 Hz, 1H), 7.65 (d, *J* = 8.3 Hz, 1H), 7.72-7.75 (m, 4H), 7.93 (d, *J* = 11.9 Hz, 2H), 7.97 (d, *J* = 8.3 Hz, 2H), 7.98-8.01 (m, 1H), 8.03-8.07 (m, 7H), 8.15 (d, *J* = 8.3 Hz, 1H), 8.23 (d, *J* = 8.3 Hz, 2H), 8.83- 8.87 (m, 8H), 9.06 (s, 1H), 10.08 (s, 1H) ppm; ^{13}C-NMR (150 MHz, $CDCl_3$) δ_C = 29.8, 31.8, 35.1, 119.2, 121.1, 121.2, 121.6, 121.7, 122.2, 122.7, 123.3, 125.6, 128.7, 129.3, 129.4, 129.8, 129.9, 130.2, 130.9, 133.3, 135.1, 135.8, 136.0, 136.7, 137.4, 141.3, 141.4, 142.8, 148.7, 148.8, 148.9, 152.5, 154.1, 155.9, 160.4, 190.3 ppm; FTIR (KBr) υ = 3310, 2959, 2915, 2861, 1715, 1595, 1467, 1362 cm^{-1}; MS (MALDI-TOF): *m/z* 1185 $[M+H]^+$.

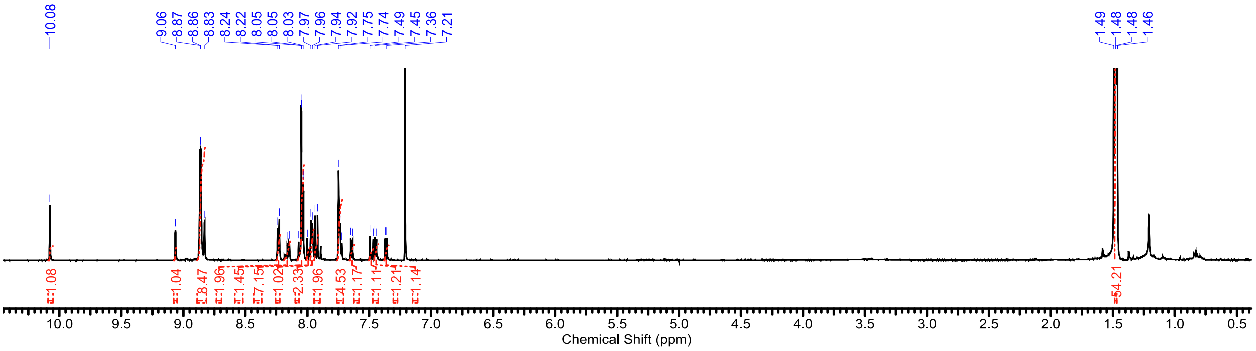

Figure F18. ^{1}H-NMR ($CDCl_3$, 600 MHz, 298 K) of compound **10b.**

Compound 11a (H_2P-3-*m*CHO): Yield (42 %). ^{1}H-NMR (600 MHz, $CDCl_3$) δ_H = -2.68 (s, 2H), 1.52 (s, 54H), 7.41 (d, *J* = 8.3 Hz, 1H), 7.46 (d, *J* = 7.33 Hz, 1H), 7.48 (d, *J* = 8.25 Hz, 1H), 7.49 (d, *J* = 8.26 Hz, 1H), 7.51-7.53 (m, 1H), 7.59 (d, *J* = 8.3 Hz, 1H), 7.73 (d, *J* = 6.4 Hz, 1H), 7.75-7.77 (m, 1H), 7.79-7.81 (m, 4H), 7.85-7.88 (m, 2H), 7.89-7.90 (m, 1H), 7.91-7.92 (m, 1H), 7.93-7.94 (m, 1H), 7.95-7.96 (m, 1H), 8.02 (d, *J* = 8.3 Hz, 1H), 8.08-8.11 (m, 7H), 8.28 (d, *J* = 7.34 Hz, 1H), 8.91-8.92 (m, 8H), 10.15 (s, 1H) ppm; ^{13}C-NMR (150 MHz, $CDCl_3$) δ_C = 29.8, 31.8, 35.1, 119.2, 120.3, 120.4, 120.9, 121.1, 121.3, 121.4, 121.5, 121.6, 121.7, 122.4, 122.5, 123.3, 123.6, 125.6, 126.7, 126.8, 129.0, 129.8, 129.9, 130.8, 131.0, 132.3, 132.4, 132.5, 132.8, 133.0, 133.8, 135.1, 136.2, 137.2, 137.3, 137.5, 141.4, 142.7, 148.8, 152.9, 153.9, 154.6, 155.1, 155.2, 155.4, 155.8, 156.0, 156.1, 156.3, 193.9 ppm; FTIR (KBr) υ = 3307, 2958, 2923, 2853, 1722, 1637, 1457, 1362 cm^{-1}; MS (MALDI-TOF): *m/z* 1288 $[M+H]^+$.

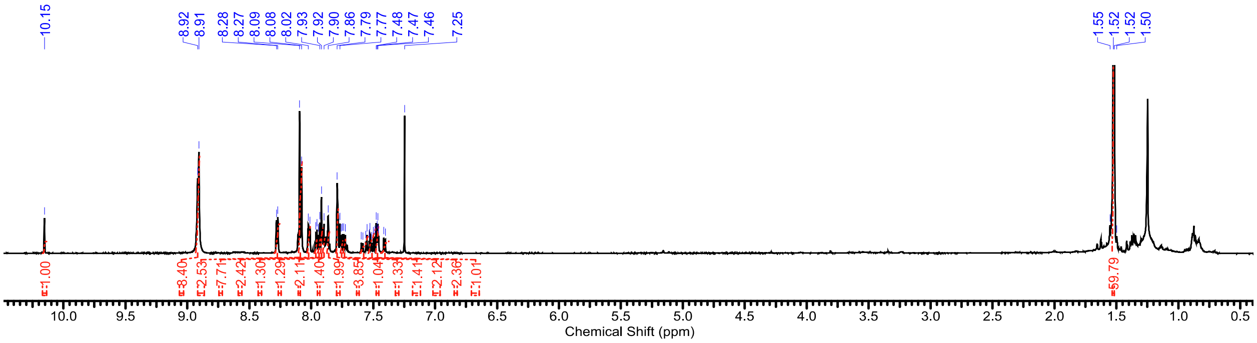

Figure S19. ^{1}H-NMR ($CDCl_3$, 600 MHz, 298 K) of compound **11a.**

Compound 11b (H_2P-3-*p*CHO): Yield (45 %). ^{1}H-NMR (600 MHz, $CDCl_3$) δ_H = -2.68 (s, 2H), 1.52 (s, 54H), 7.38 (d, *J* = 8.3 Hz, 1H), 7.41 (d, *J* = 7.3 Hz, 1H), 7.43 (d, *J* = 16.5 Hz, 1H), 7.50-7.56 (m, 2H), 7.62 (d, *J* = 8.3 Hz, 1H), 7.66 (d, *J* = 8.3 Hz, 1H), 7.73 (d, *J* = 7.3 Hz, 1H), 7.78-7.79 (m, 4H), 7.89-7.91 (m, 2H), 7.93-7.94 (m, 4H), 7.95-7.98 (m, 1H), 8.03-8.05 (dd, *J* = 8.3 Hz, *J* = 8.3 Hz, 1H), 8.08-8.09 (m, 8H), 8.18-8.20 (m, 1H), 8.27 (d, *J* = 8.3 Hz, 2H), 8.90 (s, 6H), 9.09 (s, 1H), 10.11 (s, 1H) ppm; ^{13}C-NMR (150 MHz, $CDCl_3$) δ_C = 29.8, 31.8, 32.0, 35.1, 119.0, 120.4, 120.5, 121.1, 121.5, 121.6, 121.7, 123.1, 123.3, 125.1, 125.5, 129.7, 129.9, 130.2, 130.7, 131.0, 131.8, 132.3, 132.4, 133.3, 135.1, 135.7, 136.1, 136.6, 137.4, 141.4, 141.5, 142.7, 148.8, 149.0, 152.4, 154.2, 154.3, 155.2, 160.3, 190.2 ppm; FTIR (KBr) υ = 3315, 2958, 2924, 2853, 1705, 1590, 1474, 1362 cm^{-1}; MS (MALDI-TOF): *m/z* 1288 $[M+H]^+$.

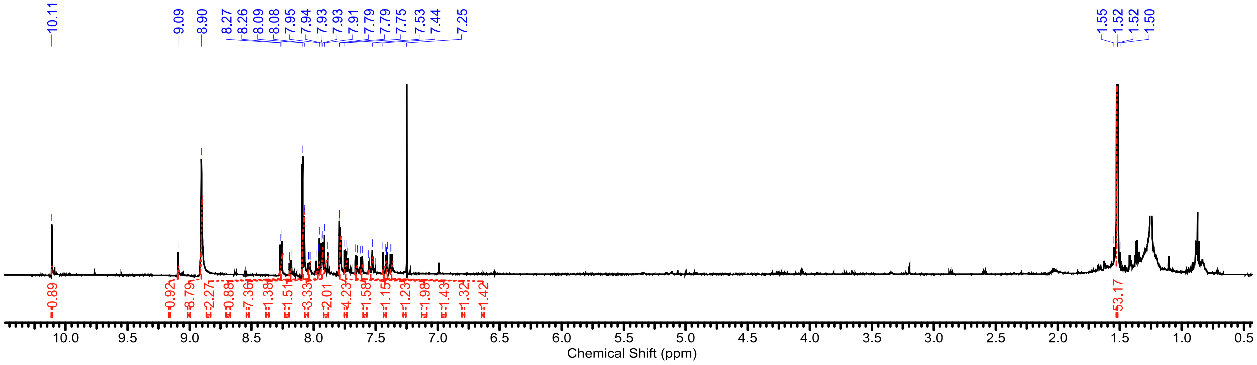

Figure S20. ^{1}H-NMR ($CDCl_3$, 600 MHz, 298 K) of compound **11b.**

Compound 12a (ZnP-1-*m*CHO): Yield (62 %). ^{1}H-NMR (600 MHz, $CDCl_3$) δ_H = 1.53 (s, 54H), 7.51 (d, *J* = 15.6 Hz, 1H), 7.70 (d, *J* = 7.3 Hz, 1H), 7.79-7.80 (m, 3H), 7.84-7.88 (m, 2H), 8.00 (d, *J* = 7.3 Hz, 2H), 8.09-8.11 (m, 7H), 8.29 (d, *J* = 7.3 Hz, 2H), 9.01-9.02 (m, 8H), 10.17 (s, 1H) ppm; ^{13}C-NMR (150 MHz, $CDCl_3$) δ_C = 29.8, 31.8, 35.1, 119.8, 120.1, 120.9, 122.6, 122.8, 125.6, 125.9, 127.4, 129.7, 129.8, 131.6, 132.3, 132.4, 132.5, 134.5, 135.0, 135.5, 137.6, 141.9, 143.8, 148.7, 150.0, 150.5, 150.6, 150.7, 152.9, 156.5, 193.9 ppm; FTIR (KBr) υ = 3009, 2958, 2924, 2859, 1707, 1590, 1461, 1363 cm^{-1}; MS (MALDI-TOF): *m/z* 1144 $[M+H]^+$.

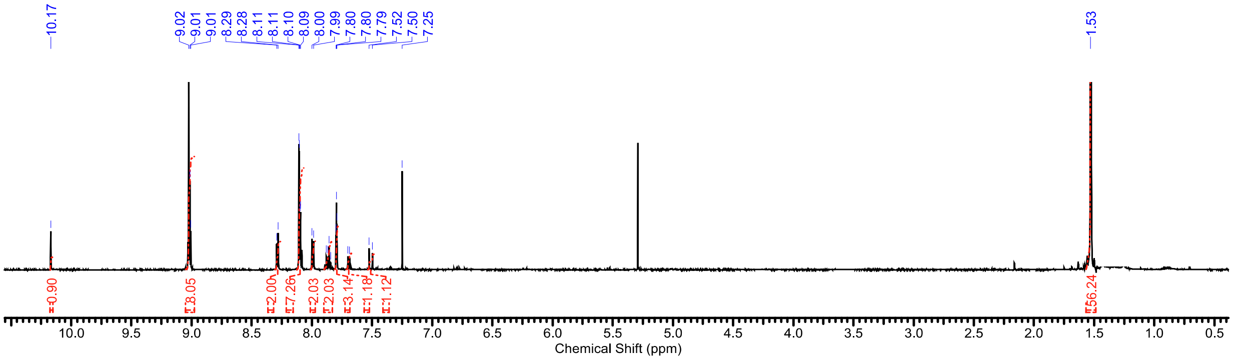

Figure S21. ^{1}H-NMR ($CDCl_3$, 600 MHz, 298 K) of compound **12a.**

Compound 12b (ZnP-1-*p*CHO): Yield (65 %). ^{1}H-NMR (600 MHz, $CDCl_3$) δ_H = 1.52 (s, 54H), 7.31-7.37 (m, 1H), 7.54-7.58 (m, 1H), 7.79 (s, 3H), 7.97 (d, *J* = 8.3 Hz, 2H), 8.09-8.15 (m, 8H), 8.28 (d, *J* = 8.3 Hz, 2H), 8.86-8.92 (m, 1H), 8.98-9.02 (m, 8H), 10.08 (s, 1H) ppm; ^{13}C-NMR (150 MHz, $CDCl_3$) δ_C = 29.8, 31.9, 35.2, 120.9, 122.4, 122.7, 122.9, 125.9, 127.3, 129.7, 129.8, 131.6, 132.3, 132.4, 132.5, 132.6, 135.1, 135.2, 136.5, 136.9, 141.9, 148.7, 148.8, 150.0, 150.5, 150.6, 150.7, 152.6, 190.2 ppm; FTIR (KBr) υ = 3008, 2957, 2905, 2867, 1704, 1588, 1475, 1361 cm^{-1}; MS (MALDI-TOF): *m/z* 1144 $[M+H]^+$.

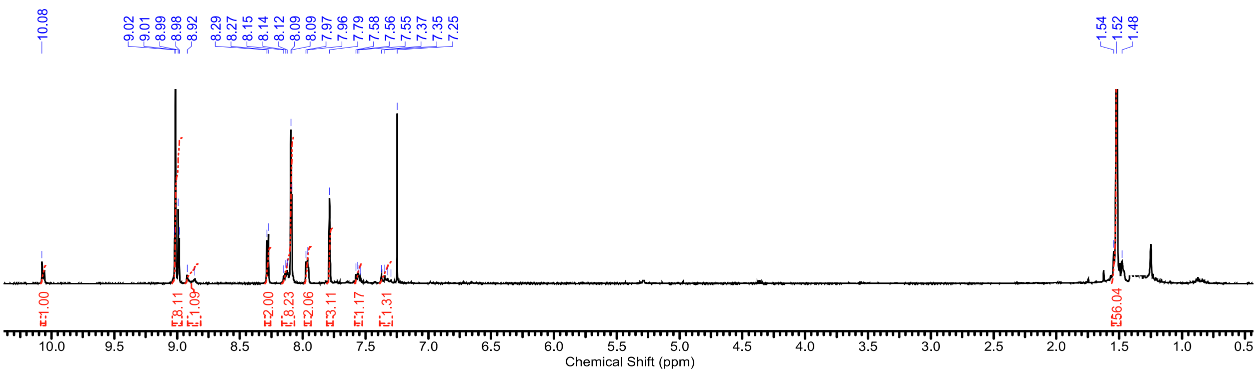

Figure S22. ^{1}H-NMR ($CDCl_3$, 600 MHz, 298 K) of compound **12b.**

Compound 13a (ZnP-2-*m*CHO): Yield (57 %). ^{1}H-NMR (600 MHz, $CDCl_3$) δ_H = 1.53 (s, 54H), 7.42 (d, *J* = 8.3 Hz, 1H), 7.48 (d, *J* = 7.3 Hz, 1H), 7.54 (d, *J* = 16.5 Hz, 1H), 7.74-7.75 (m, 1H), 7.76 (d, *J* = 7.3 Hz, 1H), 7.78-7.80 (m, 3H), 7.87-7.88 (m, 1H), 7.90 (m, 1H), 7.94 (d, *J* = 16.5 Hz, 1H), 8.01 (d, *J* = 8.3 Hz, 1H), 8.08-8.11 (m, 7H), 8.28 (d, *J* = 8.3 Hz, 1H), 9.01-9.02 (m, 8H), 10.15 (s, 1H) ppm; ^{13}C-NMR (150 MHz, $CDCl_3$) δ_C = 29.8, 31.8, 35.1, 115.6, 120.3, 120.9, 122.6, 122.7, 125.5, 126.8, 128.7, 129.7, 129.8, 131.7, 131.8, 132.3, 132.4, 133.3, 133.9, 134.9, 137.3, 137.7, 141.9, 148.6, 148.7, 148.9, 149.5, 150.1, 150.5, 150.6, 152.9, 154.5, 155.8, 156.2, 193.8 ppm; FTIR (KBr) υ = 3010, 2960, 2935, 2862, 1713, 1552, 1461, 1371 cm^{-1}; MS (MALDI-TOF): *m/z* 1247 $[M+H]^+$.

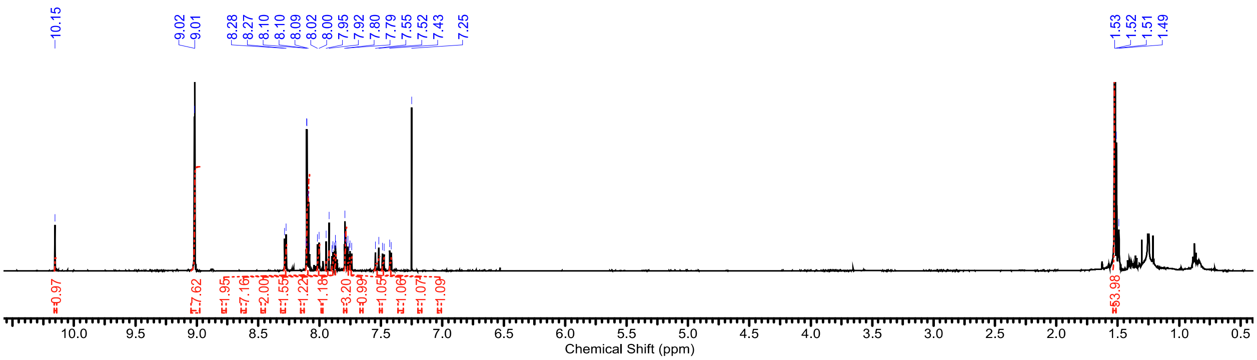

Figure S23. ^{1}H-NMR ($CDCl_3$, 600 MHz, 298 K) of compound **13a.**

Compound 13b (ZnP-2-*p*CHO): Yield (61 %). ^{1}H-NMR (600 MHz, $CDCl_3$) δ_H = 1.45-1.49 (m, 54H), 7.34 (d, *J* = 7.3 Hz, 1H), 7.44 (d, *J* = 7.3 Hz, 1H), 7.48 (d, *J* = 16.5 Hz, 1H), 7.60 (d, *J* = 8.3 Hz, 1H), 7.72 (d, *J* = 7.3 Hz, 1H), 7.73-7.75 (m, 4H), 7.85-7.87 (m, 2H), 7.97 (d, *J* = 8.3 Hz, 2H), 8.00 (d, *J* = 7.3 Hz, 2H), 8.01-8.02 (m, 1H), 8.05-8.06 (m, 7H), 8.10 (d, *J* = 5.5 Hz, 1H), 8.24 (d, *J* = 7.3 Hz, 2H), 8.84 (s, 1H), 8.84-8.98 (m, 8H), 10.02 (s, 1H) ppm; ^{13}C-NMR (150 MHz, $CDCl_3$) δ_C = 29.8, 31.8, 35.1, 120.2, 120.9, 121.1, 122.0, 122.6, 122.6, 122.7, 123.2, 125.5, 128.6, 129.4, 129.7, 129.8, 130.1, 130.8, 131.7, 132.2, 132.3, 132.4, 133.4, 134.6, 134.9, 135.9, 136.5, 137.3, 141.9, 143.4, 148.6, 148.7, 148.9, 150.1, 150.4, 150.5, 150.6, 152.4, 154.2, 156.0, 160.3, 190.2 ppm; FTIR (KBr) υ = 3008, 2959, 2924, 2853, 1708, 1546, 1461, 1383 cm^{-1}; MS (MALDI-TOF): *m/z* 1247 $[M+H]^+$.

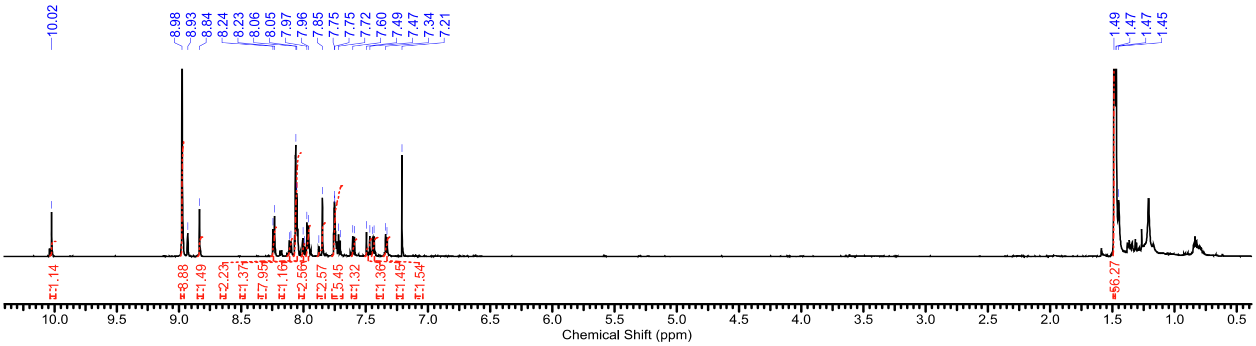

Figure S24. ^{1}H-NMR ($CDCl_3$, 600 MHz, 298 K) of compound **13b.**

Compound 14a (ZnP-3-*m*CHO): Yield (51 %). ^{1}H-NMR (600 MHz, $CDCl_3$) δ_H = 1.49-1.52 (m, 54H), 7.42 (d, *J* = 7.3 Hz, 1H), 7.47 (d, *J* = 7.3 Hz, 1H), 7.50 (d, *J* = 7.3 Hz, 2H), 7.54 (d, *J* = 16.5 Hz, 1H), 7.73-7.75 (m, 3H), 7.76-7.78 (m, 4H), 7.85-7.92 (m, 5H), 7.95 (d, *J* = 16.5 Hz, 1H), 8.02 (d, *J* = 7.3 Hz, 2H), 8.08-8.11 (m, 7H), 8.27 (d, *J* = 7.3 Hz, 2H), 9.01-9.03 (m, 8H), 10.15 (s, 1H) ppm; ^{13}C-NMR (150 MHz, $CDCl_3$) δ_C = 29.8, 31.8, 32.1, 35.1, 119.2, 120.3, 120.4, 120.9, 121.2, 121.3, 121.4, 121.5, 121.6, 121.7, 122.3, 122.5, 123.3, 123.9, 125.6, 126.8, 126.9, 129.1, 129.8, 129.9, 130.8, 131.5, 132.3, 132.4, 132.5, 132.7, 133.3, 133.8, 135.1, 136.8, 137.3, 137.6, 137.5, 141.5, 142.3, 148.9, 152.6, 153.4, 154.8, 155.2, 155.3, 155.4, 155.7, 156.0, 156.1, 156.2, 193.8 ppm; FTIR (KBr) υ = 3305, 2957, 2924, 2850, 1716, 1590, 1457, 1362 cm^{-1}; MS (MALDI-TOF): *m/z* 1350 $[M+H]^+$.

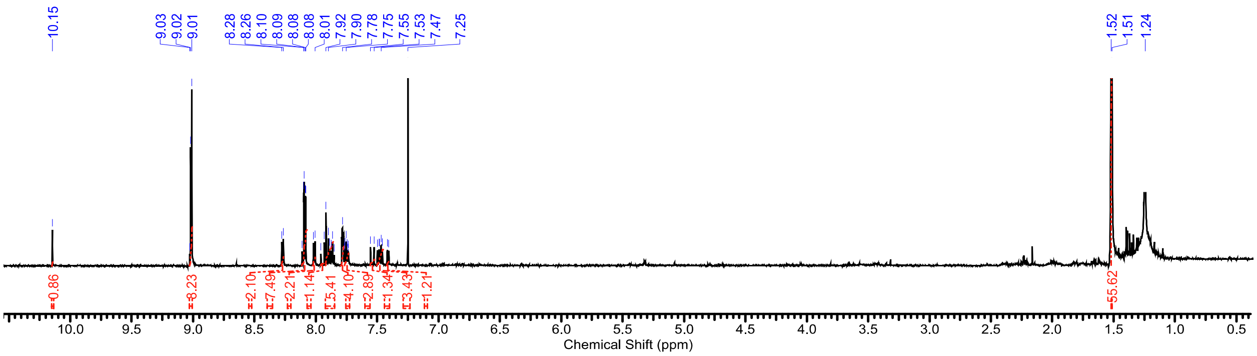

Figure S25. ^{1}H-NMR ($CDCl_3$, 600 MHz, 298 K) of compound **14a**

Compound 14b (ZnP-3-*p*CHO): Yield (50 %). ^{1}H-NMR (600 MHz, $CDCl_3$) δ_H = 1.51-1.54 (m, 54H), 7.38 (d, *J* = 7.3 Hz, 1H), 7.40 (d, *J* = 7.3 Hz, 1H), 7.43 (d, *J* = 16.5 Hz, 1H), 7.54 (d, *J* = 16.5 Hz, 2H), 7.61 (d, *J* = 8.3 Hz, 1H), 7.66 (d, *J* = 8.3 Hz, 1H), 7.72-7.73 (m, 2H), 7.75 (d, *J* = 7.3 Hz, 1H), 7.78-7.79 (m, 4H), 7.88 (d, *J* = 7.3 Hz, 1H), 7.90-7.96 (m, 5H), 8.03 (d, *J* = 5.5 Hz, 1H), 8.08-8.09 (m, 7H), 8.19 (d, *J* = 8.3 Hz, 1H), 8.27 (d, *J* = 8.3 Hz, 1H), 8.85 (s, 1H), 8.99-9.01 (m, 8H), 9.07 (s, 1H), 10.12 (s, 1H) ppm; ^{13}C-NMR (150 MHz, $CDCl_3$) δ_C = 29.8, 31.8, 35.1, 120.2, 120.9, 122.6, 122.8, 123.0, 123.1, 123.2, 125.0, 125.3, 127.6, 129.7, 129.8, 130.1, 130.7, 130.9, 131.6, 131.7, 132.3, 132.4, 132.5, 133.3, 135.0, 135.7, 135.9, 136.6, 137.4, 139.1, 142.0, 143.3, 148.7, 148.8, 150.0, 150.4, 150.5, 150.6, 152.4, 154.0, 154.1, 155.2, 160.2, 190.2 ppm; FTIR (KBr) υ = 3303, 2954, 2924, 2860, 1705, 1590, 1478, 1361, cm^{-1}; MS (MALDI-TOF): *m/z* 1350 $[M+H]^+$.

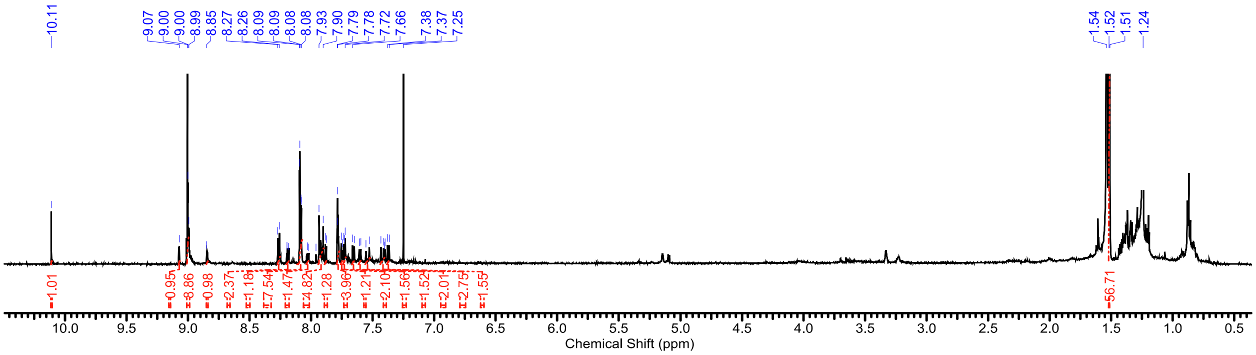

Figure S26. ^{1}H-NMR ($CDCl_3$, 600 MHz, 298 K) of compound **14b.**

4.2. ^{1}H-NMR and ^{13}C-NMR spectra for new porphyrin-fullerene conjugates **15-20a** (H_2P-n-*m*C_{60}, ZnP-n-*m*C_{60}, n=1-3) and **15-20b** (H_2P-n-*p*C_{60}, ZnP-n-pC_{60}, n=1-3).

Compound 15a (H_2P-1-*m*C_{60})

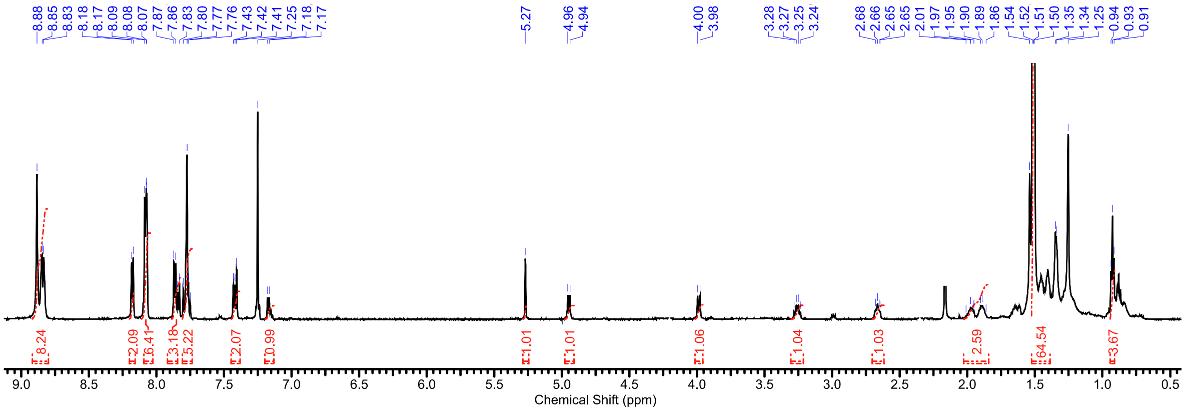

Figure S27. ^{1}H-NMR ($CDCl_3$, 600 MHz, 298 K) of **15a (H_2P-1-*m*C_{60}).**

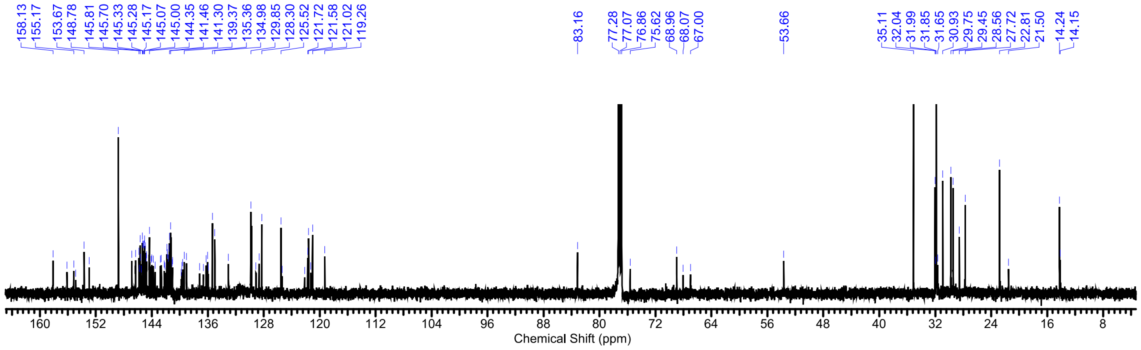

Figure S28. ^{13}C-NMR ($CDCl_3$, 150 MHz, 298 K) of **15a (H_2P-1-*m*C_{60}).**

Compound 15b (H_2P-1-*p*C_{60})

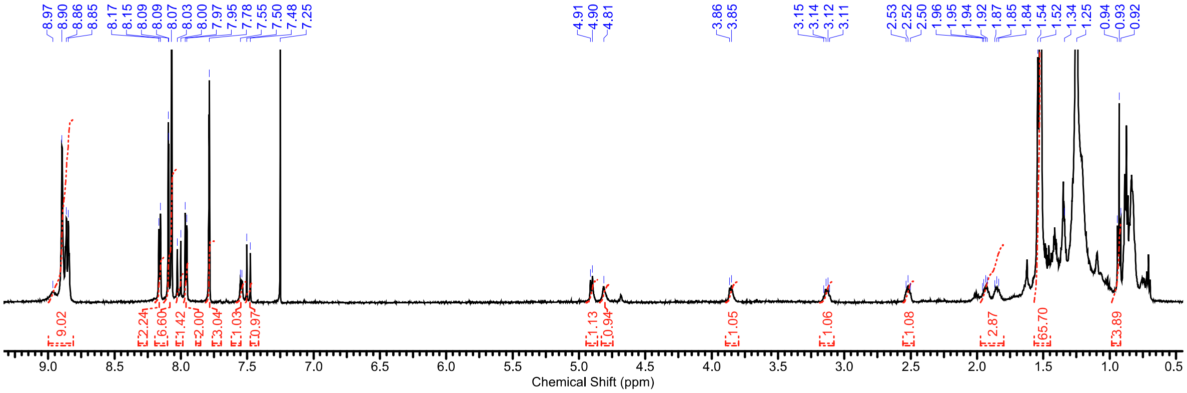

Figure S29. ^{1}H-NMR ($CDCl_3$, 600 MHz, 298 K) of **15b (H_2P-1-*p*C_{60}).**

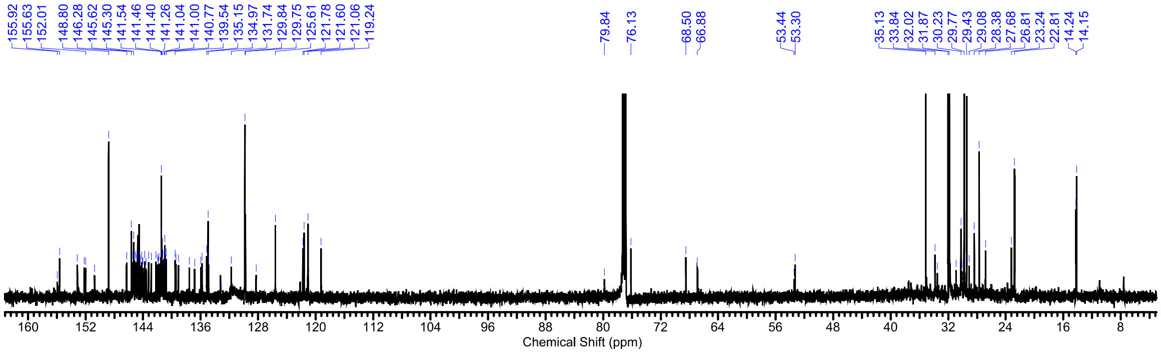

Figure S30. ^{13}C-NMR ($CDCl_3$, 150 MHz, 298 K) of **15b (H_2P-1-*p*C_{60}).**

Compound 16a (H_2P-2-*m*C_{60})

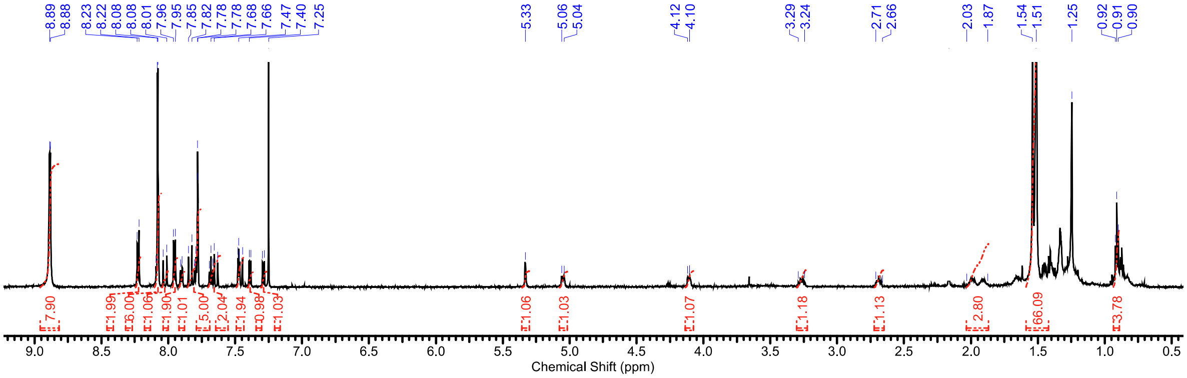

Figure S31. ^{1}H-NMR ($CDCl_3$, 600 MHz, 298 K) of **16a (H_2P-2-*m*C_{60}).**

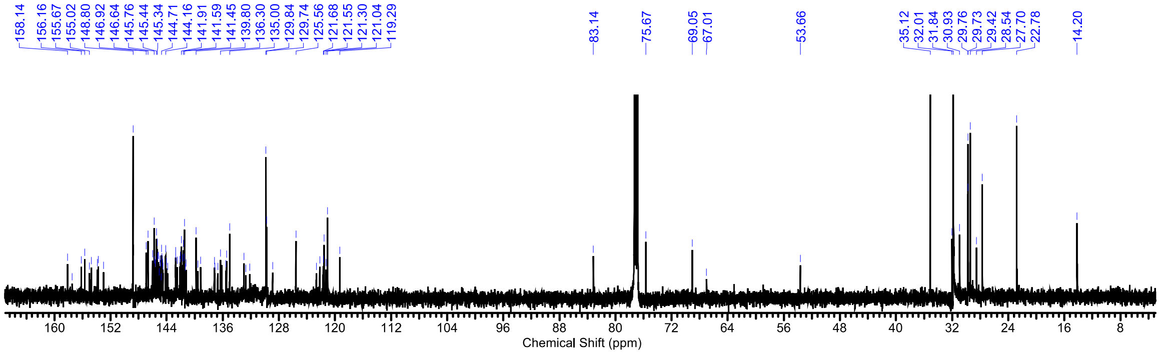

Figure S32. ^{13}C-NMR ($CDCl_3$, 150 MHz, 298 K) of **16a (H_2P-2-*m*C_{60}).**

Compound **16b** (H_2P-2-*p*C_{60})

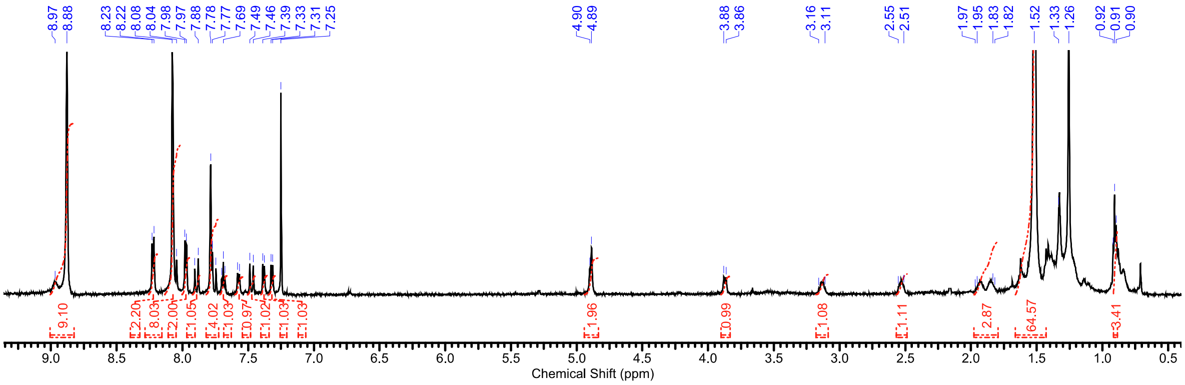

Figure S33. ^{1}H-NMR ($CDCl_3$, 600 MHz, 298 K) of **16b (H_2P-2-*p*C_{60}).**

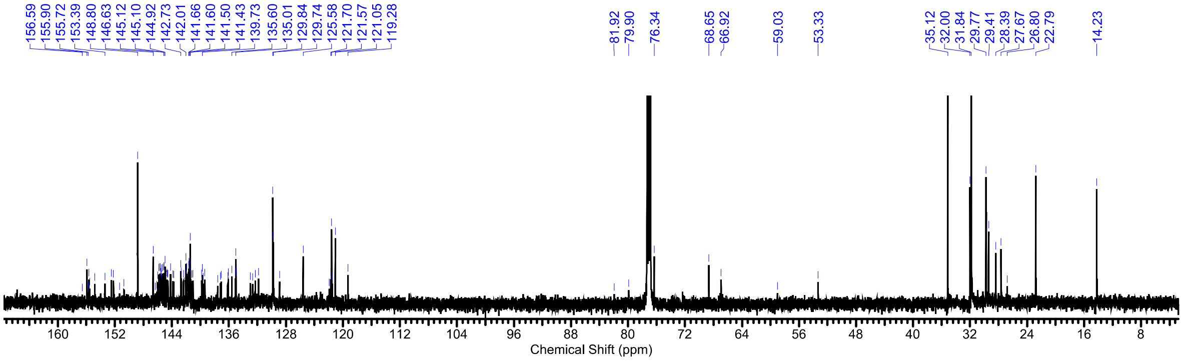

Figure S34. ^{13}C-NMR ($CDCl_3$, 150 MHz, 298 K) of **16b (H_2P-2-*p*C_{60}).**

Compound 17a (H_2P-3-*m*C_{60})

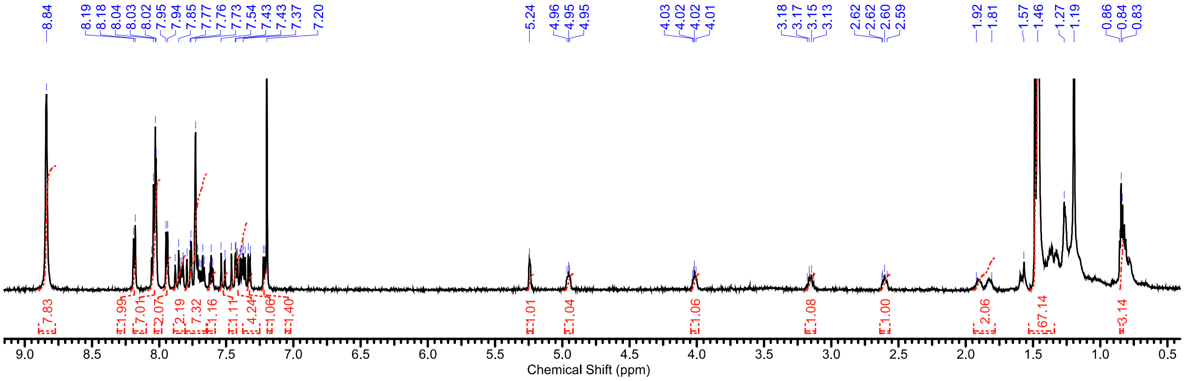

Figure S35. ^{1}H NMR ($CDCl_3$, 600 MHz, 298 K) of **17a (H_2P-3-*m*C_{60}).**

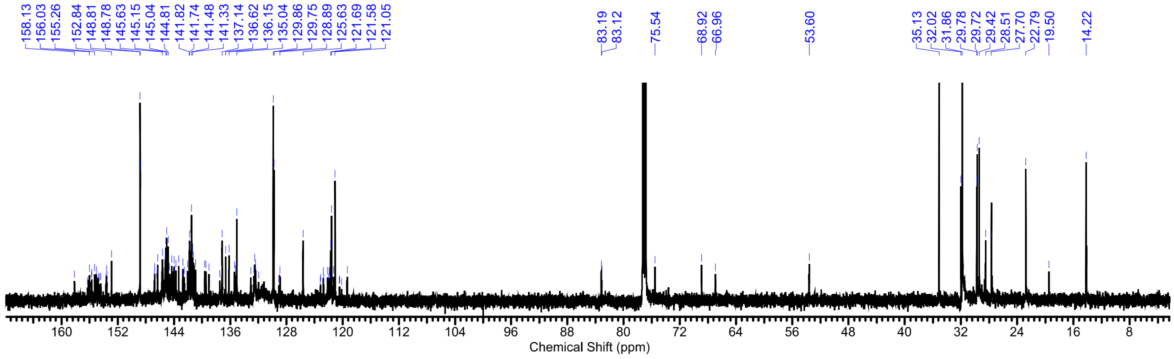

Figure S36. ^{13}C-NMR ($CDCl_3$, 150 MHz, 298 K) of **17a (H_2P-3-*m*C_{60}).**

Compound **17b** (H_2P-3-*p*C_{60})

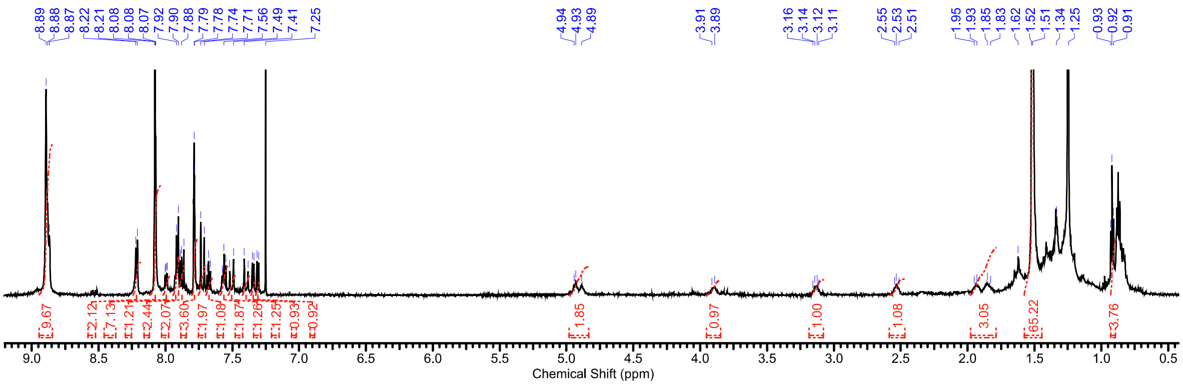

Figure S37. ^{1}H-NMR ($CDCl_3$, 600 MHz, 298 K) of **17b (H_2P-3-*p*C_{60}).**

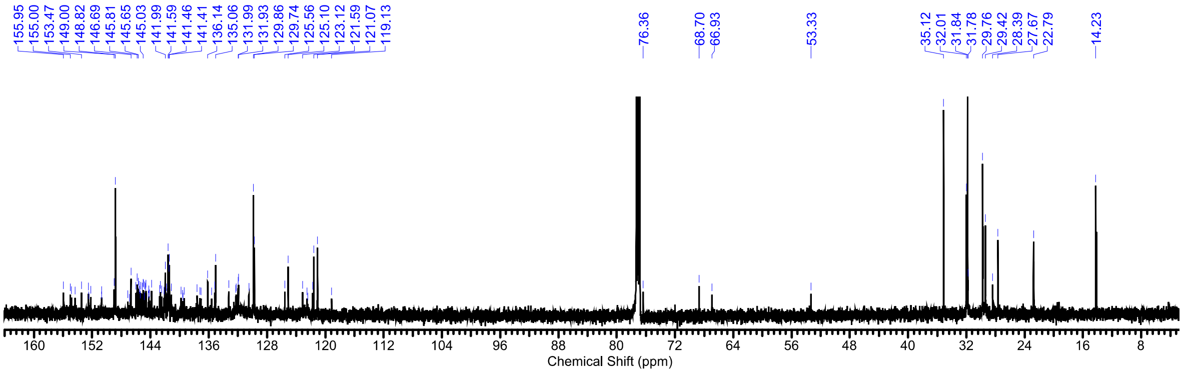

Figure S38. ^{13}C-NMR ($CDCl_3$, 150 MHz, 298 K) of **17b (H_2P-3-*p*C_{60}).**

Compound **18a** (ZnP-1-*m*C_{60})

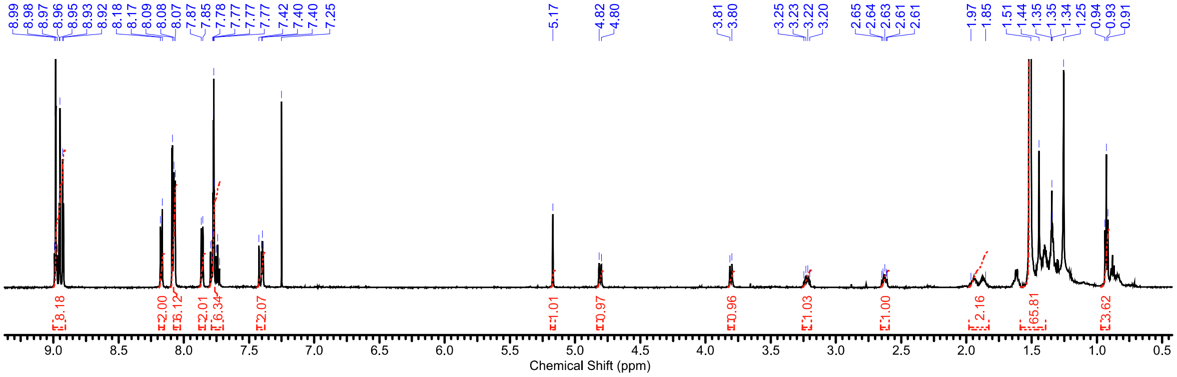

Figure S39. ^{1}H-NMR ($CDCl_3$, 600 MHz, 298 K) of **18a (ZnP-1-*m*C_{60}).**

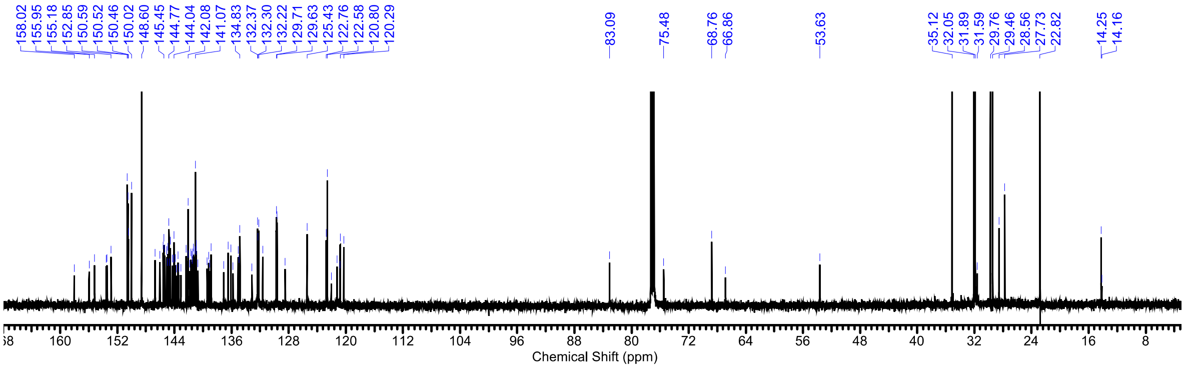

Figure S40. ^{13}C-NMR ($CDCl_3$, 150 MHz, 298 K) of **18a (ZnP-1-*m*C_{60}).**

Compound **18b** (ZnP-1-*p*C_{60})

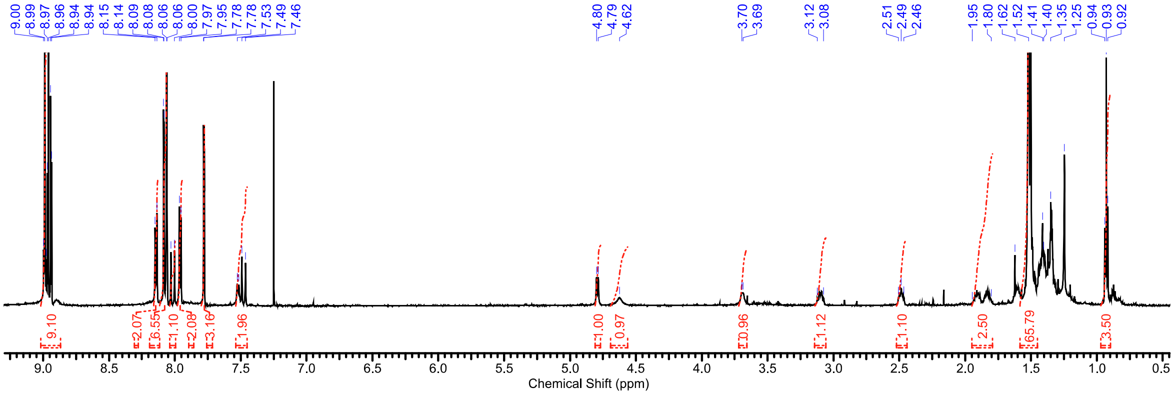

Figure S41. ^{1}H-NMR ($CDCl_3$, 600 MHz, 298 K) of **18b (ZnP-1-*p*C_{60}).**

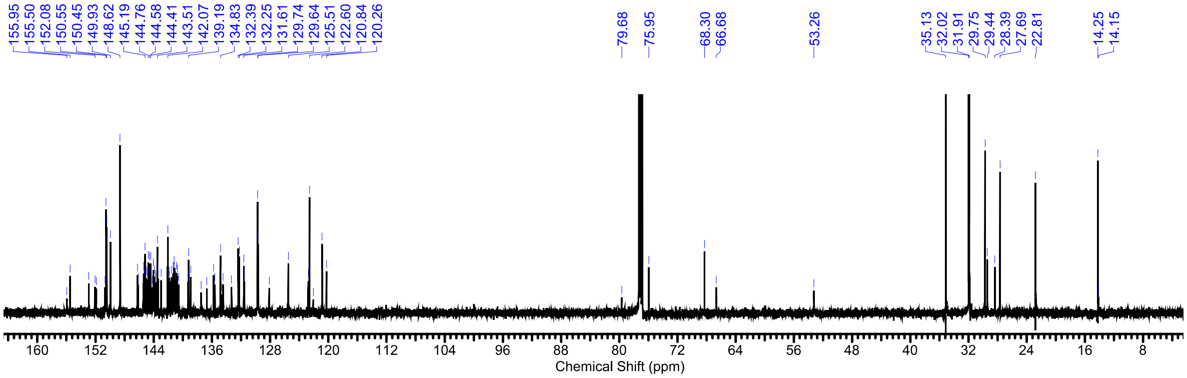

Figure S42. ^{13}C-NMR ($CDCl_3$, 150 MHz, 298 K) of **18b (ZnP-1-*p*C_{60}).**

Compound **19a** (ZnP-2-*m*C_{60}).

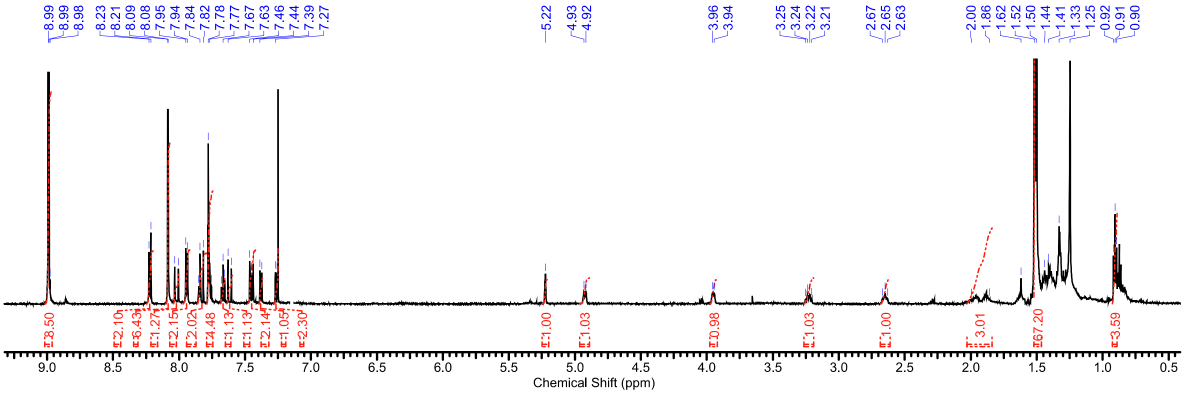

Figure S43. ^{1}H-NMR ($CDCl_3$, 600 MHz, 298 K) of **19a (ZnP-2-*m*C_{60}).**

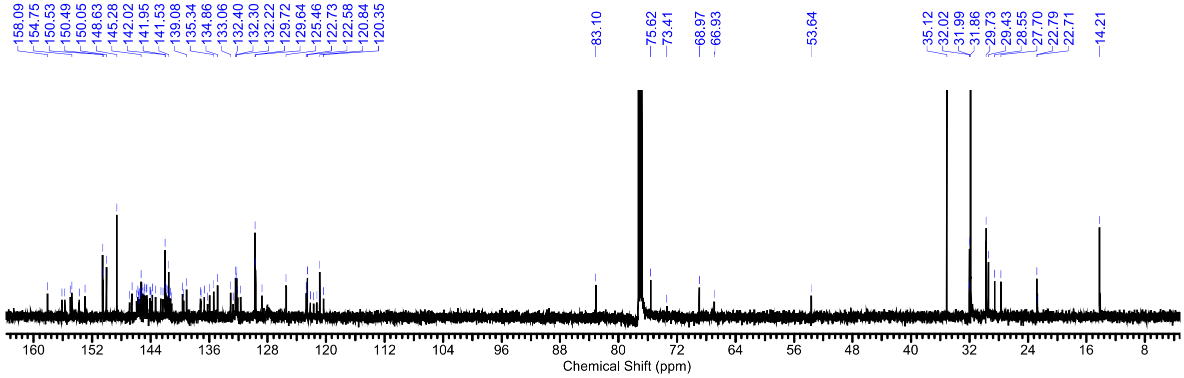

Figure S44. ^{13}C-NMR ($CDCl_3$, 150 MHz, 298 K) of **19a (ZnP-2-*m*C_{60}).**

Compound **19b** (ZnP-2-*p*C_{60})

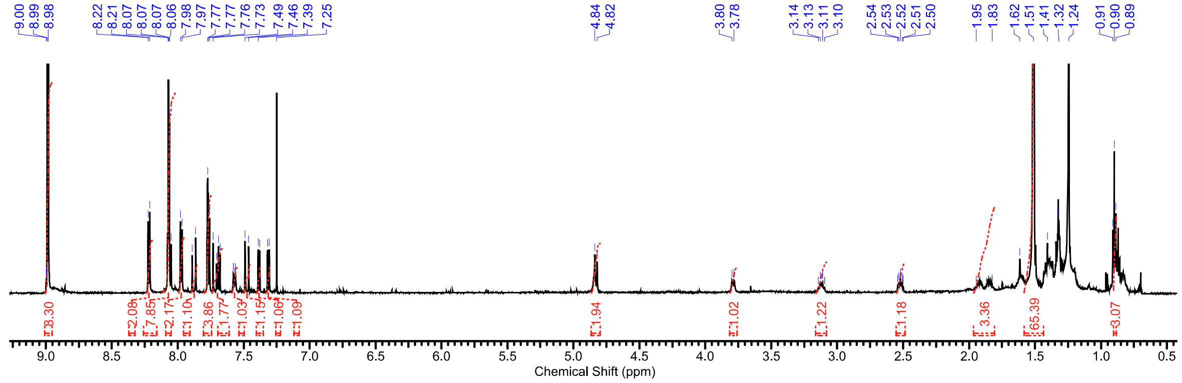

Figure S45. ^{1}H-NMR ($CDCl_3$, 600 MHz, 298 K) of **19b (ZnP-2-*p*C_{60}).**

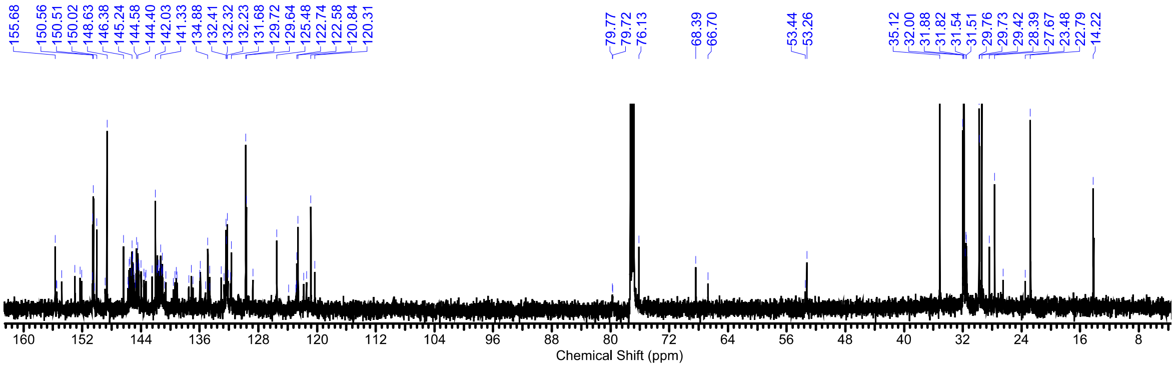

Figure S46. ^{13}C-NMR ($CDCl_3$, 150 MHz, 298 K) of **19b (ZnP-2-*p*C$_{60}$).**

Compound **20a** (ZnP-3-*m*C$_{60}$)

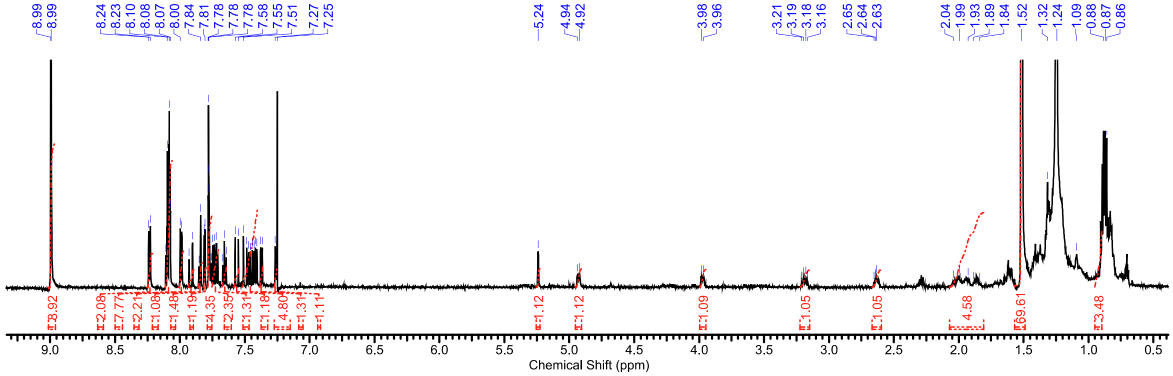

Figure S47. ^{1}H-NMR ($CDCl_3$, 600 MHz, 298 K) of compound **20a (ZnP-3-*m*C$_{60}$).**

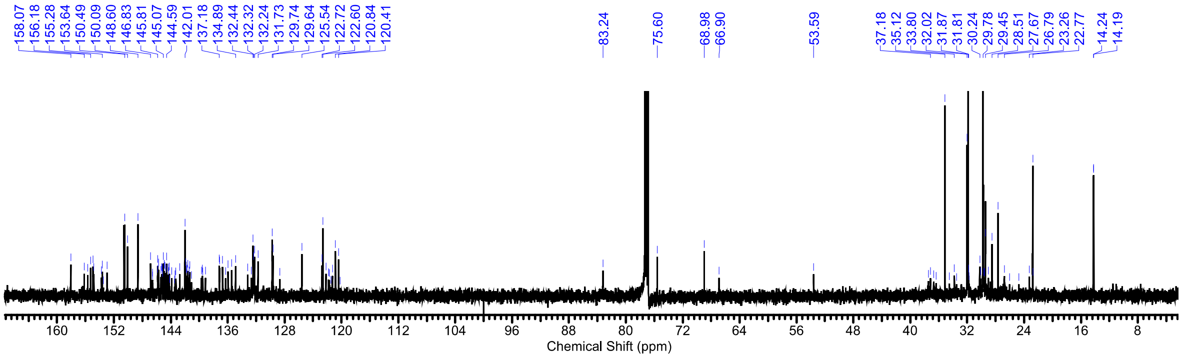

Figure S48. ^{13}C-NMR ($CDCl_3$, 150 MHz, 298 K) of **20a (ZnP-3-*m*C$_{60}$).**

Compound **20b** (ZnP-3-*p*C$_{60}$)

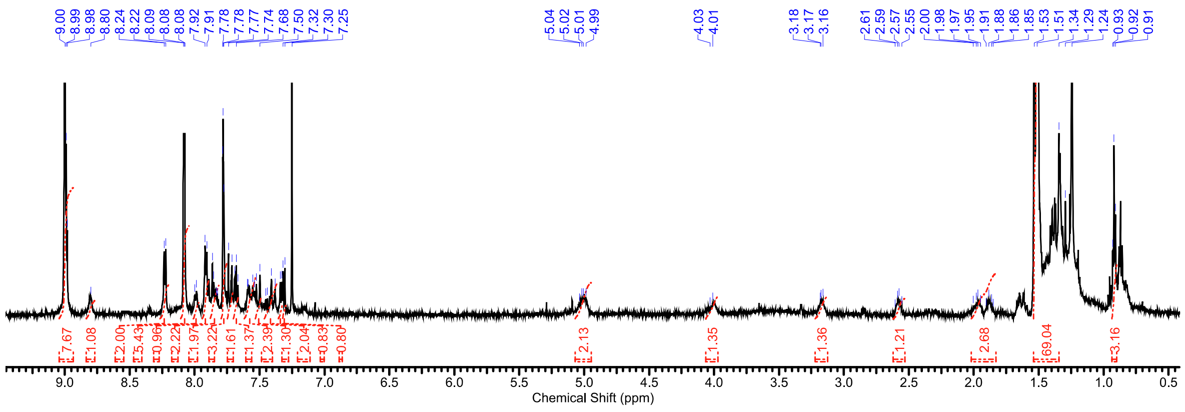

Figure S49. ^{1}H-NMR ($CDCl_3$, 600 MHz, 298 K) of compound **20b (ZnP-3-*p*C_{60})**.

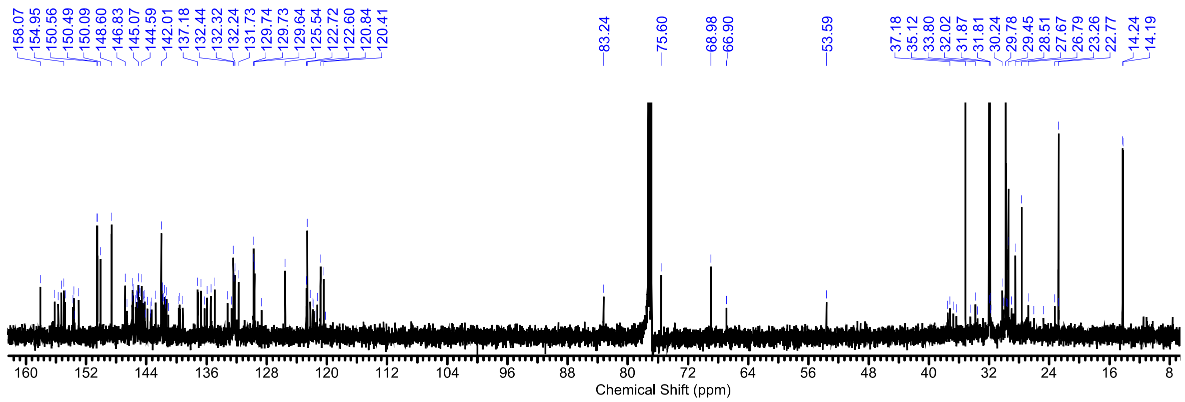

Figure S50. ^{13}C-NMR ($CDCl_3$, 150 MHz, 298 K) of **20b (ZnP-3-*p*C_{60})**.

4.3.- MALDI-TOF spectra for the new D-π-A electroactive conjugates **15-20a** and **15-20b**

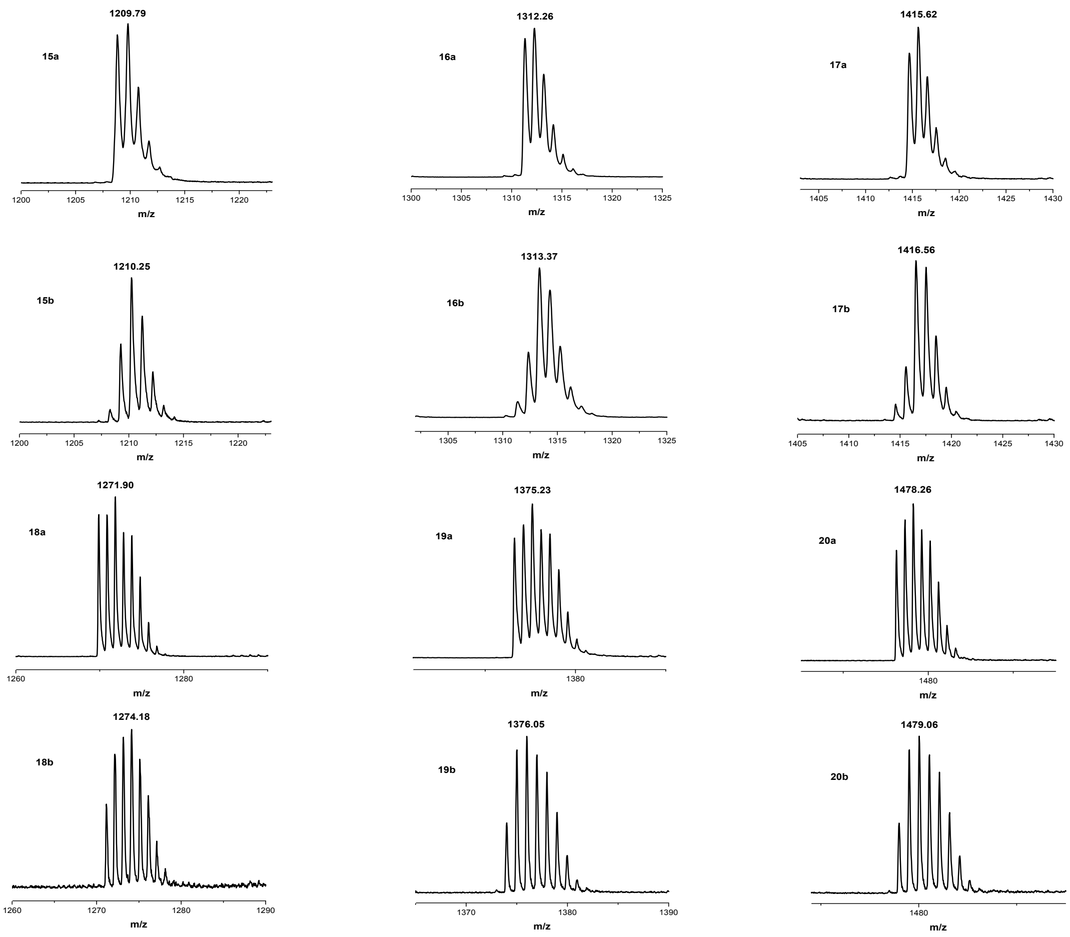

Figure S51. MALDI-TOF spectra of the new electroactive compounds **15-17a** (H_2P-n-*m*C_{60}), **15-17b** (H_2P-n-*p*C_{60}), **18-20a** (ZnP-n-*m*C_{60}) **y 18-20b** (ZnP-n-*p*C_{60}).
